# Educational weight loss interventions in obese and overweight adults with type 2 diabetes: a systematic review and meta‐analysis of randomized controlled trials

**DOI:** 10.1111/dme.14193

**Published:** 2019-12-22

**Authors:** A. Maula, J. Kai, A. K. Woolley, S. Weng, N. Dhalwani, F. E. Griffiths, K. Khunti, D. Kendrick

**Affiliations:** ^1^ Division of Primary Care School of Medicine The University of Nottingham Nottingham UK; ^2^ Leicester Diabetes Research Centre University of Leicester Leicester UK; ^3^ Real World Evidence Evidera London UK; ^4^ Division of Health Sciences Warwick Medical School University of Warwick Coventry UK; ^5^ School of Public Health University of Witwatersrand Johannesburg South Africa

## Abstract

**Aim:**

The worldwide prevalence of type 2 diabetes mellitus is increasing, with most individuals with the disease being overweight or obese. Weight loss can reduce disease‐related morbidity and mortality and weight losses of 10–15 kg have been shown to reverse type 2 diabetes. This review aimed to determine the effectiveness of community‐based educational interventions for weight loss in type 2 diabetes.

**Methods:**

This is a systematic review and meta‐analysis of randomized controlled trials (RCT) in obese or overweight adults, aged 18–75 years, with a diagnosis of type 2 diabetes. Primary outcomes were weight and/or BMI. CINAHL, MEDLINE, Embase, Scopus and the Cochrane Central Register of Controlled Trials (CENTRAL) were searched from inception to June 2019. Trials were classified into specified *a priori* comparisons according to intervention type. A pooled standardized mean difference (SMD) (from baseline to follow‐up) and 95% confidence intervals (95% CI) between trial groups (difference‐in‐difference) were estimated through random‐effects meta‐analyses using the inverse variance method. Heterogeneity was quantified using *I* ^2^ and publication bias was explored visually using funnel plots.

**Results:**

Some 7383 records were screened; 228 full‐text articles were assessed and 49 RCTs (*n* = 12 461 participants) were included in this review, with 44 being suitable for inclusion into the meta‐analysis. Pooled estimates of education combined with low‐calorie, low‐carbohydrate meal replacements (SMD = –2.48, 95% CI –3.59, –1.49, *I* ^2^ = 98%) or diets (SMD = –1.25, 95% CI –2.11, –0.39, *I* ^2^ = 95%) or low‐fat meal replacements (SMD = –1.15, 95%CI –2.05, –1.09, *I* ^2^ = 85%) appeared most effective.

**Conclusion:**

Low‐calorie, low‐carbohydrate meal replacements or diets combined with education appear the most promising interventions to achieve the largest weight and BMI reductions in people with type 2 diabetes.

## What's new?

- Weight loss improves outcomes for people with type 2 diabetes. There has been considerable expansion of related intervention research.
- This comprehensive review of 49 randomized controlled trials suggests multi‐component educational interventions in community settings are effective for weight loss in people type 2 diabetes. Education combined with low‐calorie, low‐carbohydrate or low‐fat meal replacements or diets appear to achieve the largest reduction weight and BMI.
- This study provides review‐level evidence on effective models for weight loss in people with type 2 diabetes. When specifying and delivering these interventions, education incorporating low‐calorie, low‐carbohydrate or low‐fat meal replacements should be considered.

**What's new?**

- Weight loss improves outcomes for people with type 2 diabetes. There has been considerable expansion of related intervention research.
- This comprehensive review of 49 randomized controlled trials suggests multi‐component educational interventions in community settings are effective for weight loss in people type 2 diabetes. Education combined with low‐calorie, low‐carbohydrate or low‐fat meal replacements or diets appear to achieve the largest reduction weight and BMI.
- This study provides review‐level evidence on effective models for weight loss in people with type 2 diabetes. When specifying and delivering these interventions, education incorporating low‐calorie, low‐carbohydrate or low‐fat meal replacements should be considered.

## Introduction

By 2030, it is estimated that approximately half of the world's adult population will be overweight (BMI 25–29.9 kg/m^2^) or obese (BMI > 30 kg/m^2^) 1. Obesity is associated with the development of type 2 diabetes, with figures estimating that 85–90% of people with type 2 diabetes are overweight or obese 2. Diabetes prevalence is predicted to rise to 693 million worldwide by 2045 3. As BMI increases, the associated medical costs of managing type 2 diabetes increase 4. With an estimated global economic burden of US $2.5 trillion or 2.2% of gross domestic product by 2030 5.

Weight loss in type 2 diabetes improves metabolic control and reduces the risk of complications 6, while significant weight losses of 10–15 kg have been seen to reverse diabetes 7. Traditionally, low‐carbohydrate diets have been advocated for weight loss in type 2 diabetes; however, evidence for the effects on long‐term health is conflicting 8, 9, 10. Weight loss is more difficult for people with diabetes, who lose approximately half the amount of weight compared to people without diabetes undergoing the same intervention 11; this is compounded by certain anti‐diabetic medications causing weight gain 12, 13. In people with type 2 diabetes, weight loss is often regained within 1 year of being lost 14, with a return to baseline weight frequently seen within 3–5 years 15. Bariatric surgery appears to be a cost‐effective intervention for obesity and is associated with weight losses of up to 30% at 1 year (reducing to 24% by 5 years) and reversal of type 2 diabetes 16. However, access to surgery is restricted 17, resource intensive, often requires lifelong supplementation and may not be a preferred choice for obese individuals 18. Lifestyle interventions which can achieve sustained weight loss in type 2 diabetes are therefore needed.

Previous systematic reviews of weight loss interventions in people with type 2 diabetes are now either outdated 19, 20, 21, included participants with a normal BMI 20, included only interventions of > 12 months' duration 19 or searched a limited number of databases 19. We have therefore undertaken a systematic review to determine the effectiveness of community‐based educational interventions, of any duration and follow‐up, in achieving weight loss in overweight and obese adults aged 18–75 years with type 2 diabetes. A secondary aim was to investigate whether weight loss was sustained after the intervention in trials with a maintenance component.

## Research design and methods

A detailed description of the study methods is provided in the registered protocol 22. Given the large number of studies and number of potential secondary outcomes, this review focused on the primary outcomes of weight and BMI change only.

### Information sources

MEDLINE (Ovid), Embase (Ovid), CINAHL (EBSCO), the Cochrane Central Register of Controlled Trials (CENTRAL) and Scopus were searched from inception to June 2019, with no language restrictions. The reference lists of included trials were also searched. The grey literature was not searched.

### Search strategy

The search strategy used comprised of type 2 diabetes mellitus or type 2 diabetes or t2dm AND overweight or obesity or obese AND education AND weight loss or weight reduction or lose weight. However, the search strategy varied for each database. The Cochrane Highly Sensitive Search Strategy for identifying randomized trials was used in MEDLINE: sensitivity‐ and precision‐maximizing version (2008 revision). The full search strategies are provided in Table S1.

### Study selection and data collection process

Participants had to have a diagnosis of type 2 diabetes, be aged 18–75 years with a BMI > 25 kg/m^2^, of any ethnicity, living in any country.

Educational interventions were defined as techniques using intellectual, physical and psychological methods resulting in empowerment of participants, by increasing type 2 diabetes‐related knowledge and self‐care behaviours for better disease management, with a focus on weight loss. The intervention was delivered in community settings and could be single component providing education alone or multi‐component targeting multiple health behaviours, where education was provided in addition to physical activity or diet or technology or behavioural support (counselling, coaching mindfulness), or a combination of these. Interventions were classified as counselling interventions if it was specified that components of the intervention were delivered by trained counsellors or staff with specific counselling training. The main intervention could not consist of weight loss surgery or medication.

Only randomized controlled trials (RCT) were included, with the control or comparator group receiving usual care. Weight and/or BMI was a main outcome of the trials and could be presented as a primary or secondary outcome in the trial findings. Study selection criteria are shown in Table 1.

**Table 1:** Study selection criteria

Inclusion criteria	Exclusion criteria
Study design	
Randomized controlled trials	All other study designs
Participants	
Adults aged 18–75 years with a diagnosis of type 2 diabetes and overweight (BMI 25–29.9 kg/m^2^) or obese (BMI > 30 kg/m^2^), living independently in the community (own home, warden‐controlled accommodation, extra‐care/sheltered housing, retirement communities).	Participants who are pregnant or breastfeeding, living in residential or nursing homes or inpatients in a secondary care setting.
Participants could be of any ethnicity living in any country.	
Articles written in English.	
Interventions	
Interventions provide diabetes related information with the aim of educating participants, increasing knowledge levels relating to diabetes.	Weight loss surgery or where the main intervention was weight loss medication.
Educational interventions targeting weight loss delivered in community settings, to individuals or in groups, either face to face, telephone, email, internet, post or via smart phone apps.	
Interventions may be supervised or unsupervised and individually tailored or not. Interventions may solely target weight loss or target multiple health behaviours and include dietary modification and/or physical activity promotion. Interventions may include behaviour change techniques such as motivational interviewing, counselling, goal setting, self‐monitoring or problem solving.	
Interventions providing weight loss medication in addition to education and other interventions were eligible.	
The comparators of interest include usual care or no intervention.	
Outcomes	
Objectively measured body weight in kg or lbs or BMI in kg/m^2^. This included between‐group differences and within group differences.	

Identified titles and abstracts were screened by two reviewers (AM and AW) with disagreements resolved through discussion of full‐text articles and referral to a third reviewer if necessary. Full‐text articles were obtained for all eligible trials. Multiple articles from the same trial were grouped to prevent duplication of data (Table S2), referencing the paper presenting the weight change results. Data were extracted from eligible trials by one reviewer (AM) and checked by a second (AW) using standard data extraction forms based on the TIDieR checklist 23. Extracted data related to participants and interventions (setting, procedures, materials used, single or multi‐component, duration, frequency, intensity, tailoring or modifications, delivery, follow‐up, maintenance periods and outcomes). Where not provided, weight, BMI and percentage change in weight/BMI from baseline were calculated from the data provided if possible. Risk of bias was assessed by two independent reviewers (AM and AW) using the Revised Cochrane Collaboration's risk of bias tool version 2 24, 25. Disagreements were referred to a third reviewer.

### Data synthesis

Trials were classified into specified *a priori* comparisons according to intervention type by two reviewers, due to the heterogeneity between studies. It was also decided *a priori* by study authors to not report an overall effect size from all studies due to clear methodological differences between study design and intervention types.

We estimated a pooled standardized mean difference and 95% confidence intervals (95% CI) between trial groups from baseline to follow‐up BMI and weight (difference‐in‐difference) using the inverse variance method, with random‐effects terms to account for expected methodological heterogeneity between studies. Statistical heterogeneity was assessed by *I* ^2^, given by the formula [(*Q* − df)/*Q*] × 100%, where *Q* is the statistic and df is the degrees of freedom 26. Significant statistical heterogeneity was indicated by *I* ^2^ > 70% and a $\chi^{2}$ result of *P* < 0.01 27 *.*

Where available, sample sizes, mean differences in BMI and weight change from baseline to follow‐up and standard deviations of the differences were extracted directly for each trial group from the reported findings. If mean differences were not reported, we calculated mean differences based on the raw figures reported in the studies, and standard deviation of the differences or reported confidence intervals and standard errors of the difference where possible.

All meta‐analyses were performed using Review Manager software and presented in a forest plot; funnel plots were provided to visually inspect evidence of asymmetry due to small‐study effects (evidence of publication bias). Where trials had multiple intervention groups, the main intervention group is presented in the main forest plot with additional intervention groups presented in the Supporting Information.

## Results

### Study selection

**Figure 1:**
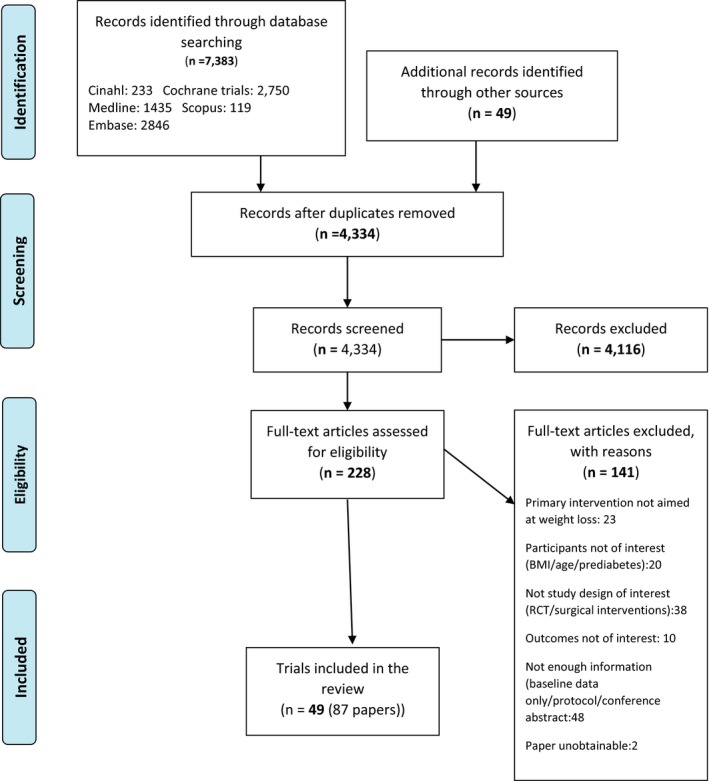
Selection of studies for inclusion into the review.

The study selection process is shown in Fig. 1. Electronic database searching identified 7383 records with an additional 49 identified through hand‐searching. After removing duplicates, 4334 records were screened for inclusion and 228 full‐text articles were reviewed for eligibility. Forty‐nine trials (reported in 87 papers, Table S3) were included in the systematic review, with 44 trials included for meta‐analysis.

### Study characteristics

The characteristics of included trials are described in Table S3. Twenty‐four trials were conducted in the USA [28–50, S1] (Doc. S1); four in Germany [S2–S5] and Australia [S6–S9]; three each in the UK [7,S10,S11] and Sweden [S12–S14]; two each in Belgium [S15,S16], Canada [S17,S18] and Finland [S19,S20]; and one each in India [S21], Italy [S22], Kazakhstan [S23], New Zealand [S24] and Spain [S25]. The 49 trials included a total of 12 461 participants. Sample sizes ranged from 27 37 to 5145 39. Mean participant age ranged from 45.7 years 44 to 66.7 years 22 and the proportion of female participants ranged from 0% to 100% 42. Twenty‐five trials [7,28–32,34–38,40–50,S1,S10,S24] described the proportion of minority ethnic participants, which ranged from 2% 7 to 100% in two trials 30, 46 with exclusive Hispanic or African‐American populations.

Mean baseline BMI was reported in 46 trials, ranging from 27 kg/m^2^ [S21] to 39.1 kg/m^2^ [S18]. Two trials reported mean baseline weight in pounds 33, 45. One trial reported a BMI range of 27–50 kg/m^2^ 38.

Five trials [31,32,36,39,S18] selected participants after a run‐in phase lasting between 3 days 36 and 6 months [S18] during which time participants confirmed their commitment by activities such as completing food and exercise diaries, or attending regular diabetes education sessions.

### Intervention description

Intervention duration ranged from 2 months 28 to 2 years [49,S9,S14,S24], most commonly lasting 12 months (*n* = 14 trials), with a median of 8 months. Trials were grouped into nine intervention types (Table 2), with education and low‐calorie diets, and education and meal replacements subdivided further. Nine trials comprised education alone, the remaining 40 were multi‐component.

**Table 2:** Characteristics of the interventions in the included trials

Intervention type	Number of trials [Refs]
Education alone	9 [33, 43, 44, 46,S6,S10,S12,S21]
Education and counselling	3 28, 30, 38
Education and low‐calorie diet	
Low carbohydrate	9 [31, 47, 48, 49,S4,S9,S11,S14,S17]
Low fat	3 [S18,S20,S24]
Counselling and physical activity	3 [36,S7,S22]
Education and modified fasting protocol	2 [S3,S23]
Education and meal replacements	
Low fat	7 [7, 29, 32, 34, 39, 50,S8]
Low carbohydrate	3 [40,S5,S25]
Education and physical activity	4 [35,S2,S13,S16]
Education and motivational interviewing	1 42
Education and mindfulness	2 [37,S1]
Education and coaching	3 [41, 45,S15]
Intervention deliverer	Number of trials
Dietician	25
Nurse	17
Physician	14
Diabetes educator	6
Exercise physiologist	4
Nutritionist	3
Counsellor	2
Community health worker	1
Physiotherapist	1
Psychologist	2
Peer Coach	1
Not stated	5
Intervention delivered by one professional	11 (psychologist [S16] physician 30, dietician, [43, 47, 49,S17,S24] peer coach 41 and diabetes coach/educator [45,S1,s5])
Intervention delivered by a team	37
Unclear who delivered intervention	1 [S23]
Duration of intervention (months)	Number of interventions and frequency of contacts
2	1 intervention with 2 contacts 28
3	10 interventions, contacts ranged from 2 [S2] to 90 45
4	4 interventions, contacts ranged from 2 [S13] to 17 [S25]
5	2 interventions, contacts ranged from 19 32 to 20 46
6	7 interventions, contacts ranged from 1 [S18] to 47 42
8	1 intervention with 20 contacts 29
10 months	3 interventions, contacts ranged from 1 [S19] to 30 48
12 months	17 interventions, contacts ranged from 3 30 to 54 38
18 months	1 intervention with 27 contacts [S7]
24 months	3 interventions with contacts ranging from 4 contacts [S14] to 21 [S9]
Tailoring of intervention	
Personalized feedback	Number of trials
	4 provided feedback on diet 30, 32, 39, 42
	4 provided feedback on physical activity [28,30,42,S19]
	3 provided feedback on weight, BMI or waist circumference [54,S15,S19]
	2 provided feedback on accelerometers step count [28,S4]
	1 provided feedback on step count [S19]
	4 provided feedback on glucose levels [28, 45,S15,S19]
Additional resources for individuals not achieving weight loss targets	Offered exercise and cooking classes, exercise equipment, food coupons or meal replacements and weight loss medication 39
	Meal replacements, individualized dietary advice and weight loss medication 7
	Extended weight loss phase if weight loss goal not achieved. [S23,S25] or calorie prescription adjusted if desired weight loss not being achieved 47
Additional contacts with intervention deliverers	5 [38, 40, 45,S14,S19]

Participant contacts were quantified in 46 trials, ranging from one contact over 6 months [S18] to 107 contacts over 12 months 38. Thirty trials described duration of each contact, which ranged from 5 min 41 to 150 min [35,37,S12]. The intervention was delivered by one educator (*n* = 11 trials) or a team of educators (*n* = 37 trials), most commonly dieticians, nurses and physicians. Fifteen trials tailored the intervention by providing personalized feedback, additional resources for those not achieving weight loss targets, additional intervention contacts or individual tailoring of the weight loss phase, which could include extending the duration of weight loss or adjustments in calorie prescription dependent on weight loss. Six trials had multiple intervention groups [33,36,40,45,S6,S8]. Education was delivered on a one‐to‐one basis (*n* = 21 trials), in group sessions (*n* = 10 trials) or in individual and group sessions (*n* = 17 trials); this was not specified in one trial [S21]. Education was delivered using a single mode in 35 trials; in 30 trials education was face‐to‐face and in five it was via telephone. Thirteen trials used multiple modes including face‐to‐face, telephone, e‐mail, post, texts, online messaging and smart phone apps. One trial did not state mode of delivery [S21]. Additional materials were provided in 24 trials, 19 provided educational information, food menus, a portion‐controlled plate, nutrition guides or a waist circumference tape measure, and 22 provided pedometers, accelerometers, games consoles, food scales, exercise videos, smart phone apps, glucose meters or meditation CDs.

### Description of maintenance component

A maintenance intervention was delivered in seven trials, consisting of two contacts over 3 months reviewing key principles, participant progress and barriers to change 37. Two contacts over 8 months, with facilitated group work addressing facilitators and barriers [S10], and monthly education sessions for 12 months 29, 42. Monthly face‐to‐face contacts over 17 months with meal replacements dependent on the amount of weight regained; if weight regain was > 4 kg, full diet replacement was given for 4 weeks, weight loss medication was available 7, and at least 84 contacts over 7 years, including monthly face‐to‐face contact or telephone/e‐mail contact, reviewing physical activity goals, meal replacement vouchers (one/day) and competitions to lose weight 39. One trial delivered counselling focused on barriers to change over 3 months but did not specify the number of contacts 33.

#### Risk of bias of included trials

The number of trials considered to be high risk or of some concern for risk of bias varied across the risk of bias domains: randomization process, *n* = 32; deviations from the intended interventions, *n* = 37; missing outcome data, *n* = 19; measurement of the outcome, *n* = 5; and selection of reported result, *n* = 39. Overall, all trials were found to be at high risk or some concern of risk of bias. However, it would not have been possible to blind participants, intervention deliverers or outcome assessors to treatment group allocation in most behaviour change trials and this has had a negative impact on the assessment of overall trial quality (Table S4 and Fig. S1).

### Measurements of treatment effect

Outcome data were reported at the end of the intervention in 20 trials; the remaining trials reported data at multiple time points (Table S5).

Forty‐one trials reported weight or change in weight from baseline, 26 trials reported BMI or change in BMI from baseline, and 18 trials reported both weight and BMI or changes from baseline. Outcome reporting varied between trials, including within‐group change from baseline, between‐group differences in weight/BMI or between‐group differences in change from baseline. Details on primary and secondary outcomes for each trial are given in Table S3.

### Weight and BMI

**Figure 2:**
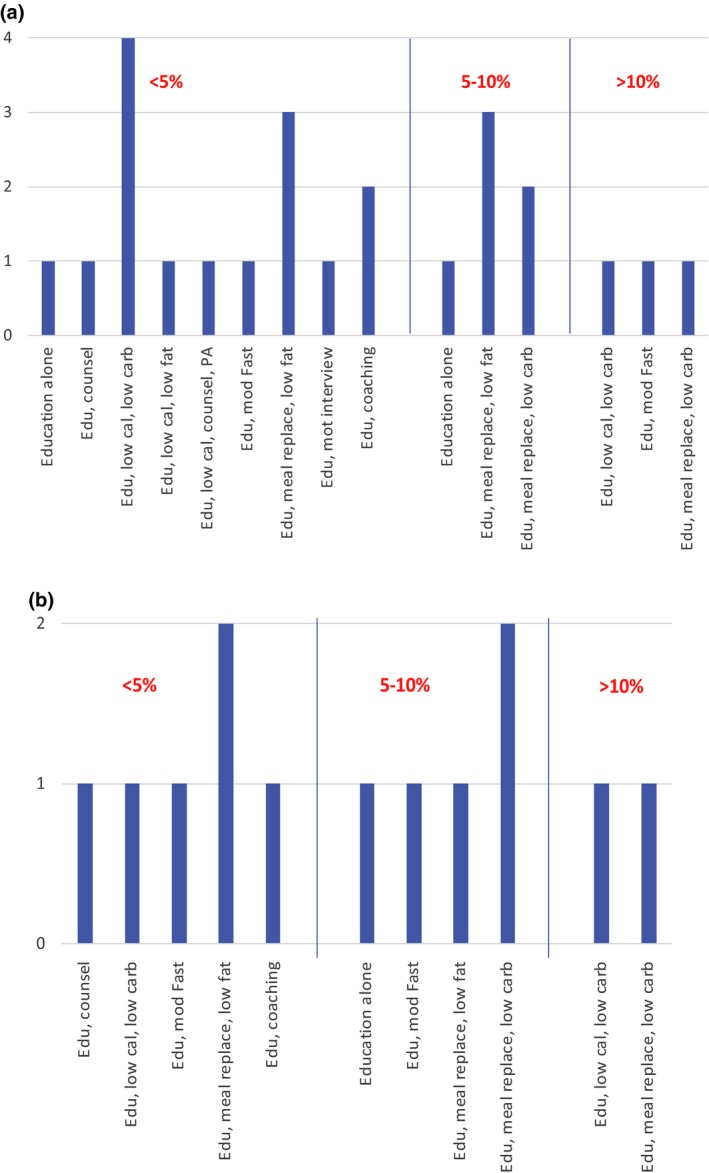
Trials reporting significant between‐group differences in (a) weight loss and (b) BMI reduction. Edu: education, PA: physical activity, low cal: low calorie, low carb: low carbohydrate, counsel: counselling, mod fast: modified fasting, meal replace: meal replacements, mot interview: motivation interviewing

Fifteen trials saw a < 5% between‐group difference for weight loss favouring the intervention group at the end of the trial, six achieved a 5–10% difference and three achieved > 10% weight loss difference between groups from baseline favouring the intervention group (Fig. 2a). Those achieving the greatest weight loss difference between groups were two low‐carbohydrate trials (education, low‐calorie and education, meal replacements) and a trial with a modified fasting regime.

Six trials saw a < 5% difference between groups for BMI reduction favouring the intervention group, five trials saw a 5–10% difference and two saw a >10% difference in BMI at the end of the intervention favouring the intervention group (Fig. 2b). Those achieving the greatest BMI differences between groups favouring the intervention group of > 10% were both carbohydrate‐restricted interventions, one was an education, low‐calorie, low‐carbohydrate diet and another utilized a low‐carbohydrate meal replacement.

All five trials pre‐selecting participants showed significant effects on weight/BMI [31,34,36,39,S18]. All five trials providing extra contacts with intervention staff, if required, saw significant effects on weight [38,40,45,S14,S19]. Eight of the nine trials providing personalized feedback showed significant effects on weight/BMI [28,32,39,42,45,S4,S15,S19].

### Trials reporting no significant effects on weight or BMI

Thirteen trials found no significant between‐ or within‐group difference in weight and/or BMI or change in weight and/or BMI from baseline [30,35,41,44,47–49,S1,S6,S9,S12,S16,S24]. These trials typically provided minimal contact time with interventionists, with five trials having on average fewer than one contact per month [30,44,S6,S12,S24]. Four trials with predominantly minority ethnic populations (87% 44, 94% 41 and 100% [30,S1]) failed to find significant effects on weight/BMI favouring the intervention group.

### Trials with prolonged follow‐up but no maintenance intervention

Ten trials [32,47,S5,S7,S8,S15,S16,S20,S23,S24] had a prolonged follow‐up period in which no maintenance intervention was delivered. This ranged from 6 months [32,S7,S16,S23] to 18 months [S8].

Two trials showed significant between‐group differences at the end of the intervention [S7,S15]; however, at 6‐ and 12‐month follow‐up the results became non‐significant. One trial continued to show significant between‐group difference in change in weight from baseline at the end of the follow‐up at 12 months 32 and one trial continued to show significant change in weight and BMI from baseline at 12 months [S5]. One of the six trials [S16] did not find a significant reduction in weight/BMI. Two trials did not present data for the end of the follow‐up period [47,S23], and one combined intervention and control group data at the end of the intervention and follow‐up periods [S8].

### Meta‐analyses of trials

All meta‐analyses are reported in Figs S2 and S3 grouped into 12 *a priori* comparisons of standardized mean differences (SMD) (BMI reported in kg/m^2^, weight in kg) between baseline and follow‐up between intervention and control groups (difference‐in‐difference).

#### Education alone

There were nine trials of education alone, eight of which could be synthesized in the meta‐analyses. The ninth trial lacked control group data [S21]. Four trials were conducted in the USA 33, 43, 44, 46, with one each in Australia [S6], Finland [S19], Sweden [S12] and the UK [S10]. Sample sizes ranged from 48 [S19] to 241 [S6]. Duration of interventions ranged from 3 months 33 to 12 months [44,43,S6,S12,S19].

Pooled results show education alone reduced weight significantly (SMD –0.63, 95% CI –1.00 to –0.26, *n* = 5 studies; *I* ^2^ = 60%), but not BMI (SMD –0.87, 95% CI –1.83 to 0.09, *n* = 4 studies; *I* ^2^ = 86%).

#### Education and counselling

Three trials of education and counselling were included within the meta‐analysis; all were conducted in the USA 28, 30, 38. Sample size ranged from 52 28 to 563 38. Duration of intervention was 2 months 28 or 12 months 30, 38.

Pooled results show that education and counselling did not significantly reduce weight (SMD –0.73, 95% CI –1.89 to 0.42, *n* = 3 studies; *I* ^2^ = 98%). In a single trial, education and counselling also did not significantly reduce BMI (SMD –0.66, 95% CI –1.32 to 0.00, *n* = 1 study; *I* ^2^ = not available).

#### Education and a low‐calorie, low‐carbohydrate diet

Nine trials had interventions consisting of education and a low‐calorie, low‐carbohydrate diet, eight of which were included in the meta‐analyses. One trial 49 did not report standard deviations.

Three trials were from the USA 31, 47, 48, with one each from Australia [59], Canada [S17], Germany [S4], Sweden [S14] and the UK [S11]. Sample size ranged from 61 [S14] to 115 [S9]. Duration of intervention ranged from 3 months [S11] to 24 months [S9,S14].

Pooled results showed education and a low‐calorie, low‐carbohydrate diet significantly reduced weight (SMD –1.25, 95% CI –2.11 to –0.39, *n* = 8 studies; *I* ^2^ = 96%) and BMI (SMD –1.32, 95% CI –3.71 to 1.08, *n* = 3 studies; *I* ^2^ = 95%).

#### Education and a low‐calorie, low‐fat diet

Three trials were included; one each in Canada [S18], Finland [S20] and New Zealand [S24]. Sample sizes ranged from 86 [S20] to 419 [S24]. Duration of intervention ranged from 6 months [S18] to 24 months [S24].

Pooled results show education, low‐calorie and a low‐fat diet significantly reduced weight (SMD –0.44, 95% CI –0.61 to –0.27, *n* = 2 studies; *I* ^2^ = 0%) and BMI in a single trial (SMD –1.00, 95% CI –1.46 to –0.54, *n* = 1 study; *I* ^2^ = not available).

#### Education, low‐calorie diet, counselling and physical activity

A single trial from Italy [S22] with 30 participants, and intervention length of 3 months was included. Other trials lacked change from baseline data [S7] and lack of standard deviation data in the control group 36. The results show education, low‐calorie diet, counselling and physical activity did not significantly reduce weight (SMD –0.34, 95% CI –1.06 to 0.39, *n* = 1 study; *I* ^2^ = not available) in this single study.

#### Education and modified fasting

Two trials were included within the meta‐analysis, one from Germany with 46 participants over 4 months [S3] and one from Kazakhstan [S23] with 272 participants over 6 months. The latter showed much greater reductions in weight and BMI in the intervention group than in the control group, most likely accounting for the large SMD values.

The pooled results showed that education and modified fasting did not significantly reduce weight (SMD –3.54, 95% CI –9.85 to 2.78, *n* = 2 studies; *I* ^2^ = 100%) or BMI (SMD –1.86, 95% CI –4.14 to 0.43, *n* = 2 studies; *I* ^2^ = 92%).

#### Education and low‐calorie, low‐fat meal replacements

There were seven trials. Six from the USA 29, 32, 34, 39, 40, 50 and one from the UK 7. Sample sizes ranged from 49 7 to 5145 39. Intervention length ranged from 3 months 34 to 24 months 7. One trial was excluded as it lacked control group data from baseline. [S8]

Pooled results show that education and low‐calorie, low‐fat meal replacements significantly reduced weight (SMD –1.15, 95% CI –1.41 to –0.89, *n* = 7 studies; *I* ^2^ = 82%) and BMI (SMD –1.57, 95% CI –2.05 to –1.09, *n* = 3 studies; *I* ^2^ = 85%).

#### Education and low‐calorie, low‐carbohydrate meal replacements

Three trials were included in the meta‐analysis; one from each of Germany [S5], Spain [S25] and the USA 40. Sample size ranged from 89 [S25] to 227 40 with intervention duration ranging from 3 months [S5] to 12 months 40.

Pooled results show education and low‐calorie, low‐carbohydrate meal replacements significantly reduced weight (SMD –2.48, 95% CI –3.79 to –1.16, *n* = 3 studies; *I* ^2^ = 96%) and BMI (SMD –2.54, 95% CI –3.59 to –1.49, *n* = 3 studies; *I* ^2^ = 98%).

#### Education and physical activity

Four trials were included; one from each of Belgium [S16], Germany [S2], Sweden [S13] and the USA 35. Sample size ranged from 50 [S13] to 220 [S2]. Intervention duration ranged from 3 months [35,S2] to 6 months [S16].

Pooled results show that education and physical activity did not significantly reduce weight (SMD –0.14, 95% CI –0.35 to 0.07, *n* = 3 studies; *I* ^2^ = 0%) or BMI (SMD –0.14, 95% CI –0.34 to 0.05, *n* = 4 studies; *I* ^2^ = 0%).

#### Education and motivational interviewing

There was one trial from the USA 42 with 217 participants conducted over 6 months. In this trial, education and motivational interviewing reduced weight significantly (SMD –0.33, 95% CI –0.60 to –0.06, *n* = 1 study; *I* ^2^ = not available).

#### Education and mindfulness

Two trials were included, both from the USA. One had 52 participants with an intervention over 3 months 37 and the other had 111 participants conducted over 12 months [S1].

Pooled results showed that education and mindfulness did not significantly reduce weight (SMD 0.99, 95% CI –2.16 to 4.13, *n* = 2 studies; *I* ^2^ = 98%). In one trial, the intervention actually increased BMI significantly (SMD 0.51, 95% CI 0.40 to 0.62, *n* = 1 study; *I* ^2^ = not available).

#### Education and coaching

There were three trials on education and coaching. One from Belgium [S15] and two from the USA 41, 45. Sample size ranged from 221 45 to 574 [S15], with intervention length ranging from 3 months 45 to 10 months 41.

Pooled results showed that education and coaching did not significantly reduce weight (SMD –0.19, 95% CI –0.61 to 0.23, *n* = 2 studies; *I* ^2^ = 82%) or BMI (SMD –0.12, 95% CI –0.76 to 0.52, *n* = 2 studies; *I* ^2^ = 85%).

#### Publication bias

Funnel plots are visually symmetrical showing limited effects of publication bias (Fig. S4).

### Maintenance

Seven trials [7,29,33,37,39,42,S10] contained a maintenance component. Three reported significant between‐group effects in weight/BMI at the end of the intervention and maintenance periods favouring the intervention group 7, 39, 42. Two trials reported significant change in weight from baseline at the end of both the intervention and maintenance periods in the intervention groups 29, 37; one of the two trials 37 also showed a significant change from baseline in the control group at the end of the maintenance period that was larger than that in the intervention group. All five trials reported some regain of weight/BMI during the maintenance period, with change in weight from baseline reducing from 10% to 7.2% 7, 8.6% to 4.7% 39, 4.8% to 3.6% 42, 7.1% to 5.5% 29 and 1.7% to 1.4% 37 at the end of the intervention and maintenance periods, respectively.

## Discussion

### Main findings

To our knowledge, this is the largest contemporary review assessing the evidence for educational weight loss interventions in obese or overweight individuals with type 2 diabetes.

This meta‐analysis has shown that 6 of 12 intervention categories were significantly effective for weight loss. The most effective interventions were: education, low‐calorie, low‐carbohydrate meal replacements, –2.48 [–3.79, –1.16]; followed by education, low‐calorie low‐carbohydrate diet, –1.25 [–2.11, –0.39]; education, low‐calorie, low‐fat meal replacements, –1.15 [–1.41, –0.89]; education alone, –0.63 [–1.00, –0.26]; education with a low‐calorie and low‐fat diet, –0.44 [–0.61, –0.27]; and finally education and motivational interviewing, –0.33 [–0.60, –0.06].

For BMI reduction, 3 of 12 intervention categories were significantly effective. Education, low‐calorie, low‐carbohydrate meal replacement was most effective, –2.54 [–3.59, –1.49], with education, low‐calorie, low‐fat meal replacements the second most effective, –1.57 [–2.05, –1.09], followed by education and a low‐calorie, low fat diet, –1.00 [–1.46, –0.54].

Meal replacements, specifically low‐carbohydrate followed by low‐fat varieties, and then low‐calore, low‐carbohydrate diets appeared the most effective tools for reducing weight, a low‐fat diet in meal replacements or alone as a low‐calorie diet appeared to be less effective when compared with a low‐carbohydrate comparison.

Non‐significant groups of interventions within the meta‐analysis may have been due to significant heterogeneity, small sample sizes, small number of trials within each category and low‐quality studies.

Five trials with a maintenance intervention (3 months to 7 years) showed significant weight reduction or change in weight/BMI at both the end of the intervention and maintenance periods, with weight regain during the maintenance phases.

### Comparison with existing literature and explanations

Our findings suggest that trials containing low‐calorie meal replacements or diets in combination with education had the greatest effect, consistent with previous reviews, showing multi‐component intensive interventions containing low‐calorie diets were the most effective for weight loss 20. Meal replacements have also been found to achieve greater weight loss in people without diabetes compared with portion‐ or calorie‐restricted diets [S26–S29]. Obese individuals may underestimate calorie consumption when consuming conventional foods [34,S28] even when given a daily calorie limit, and meal replacements avoid this. Recent trials show remission of diabetes in 58% of participants on a low‐carbohydrate diet [S30]. A recent review and meta‐analysis found ‘little to no difference’ between low‐carbohydrate and low‐fat diets on metabolic control [S31]. A second recent meta‐analysis found that compared with high‐carbohydrate interventions, low‐carbohydrate diets had the greatest effect on metabolic control within the first year of the intervention with no effect on weight [S32].

We found that interventions containing personalized feedback (individualized advice to each participant based on diet, physical activity, weight/BMI, step counts and glucose levels) were associated with significant weight loss. In people without diabetes, short‐term individualized feedback on fitness and health may influence an individual's awareness of their behaviour [S33]. Another review suggested a positive impact on weight loss for internet‐delivered interventions of < 12 months' duration providing personalized feedback in overweight and obese individuals [S34]. We note that trials containing a pre‐selection [32,36,39,S29] component (where participants had to confirm commitment to the intervention by completing self‐monitoring activities lasting 3–28 days, or attending regular educational sessions over a 6‐month period) all showed significant weight reductions. These trials may be recruiting those who are ready and motivated to change, and their findings may be less generalizable to the wider population of adults with type 2 diabetes.

### Strengths and limitations

We have identified more trials than preceding systematic reviews in this field, yielding data for a greater number of participants We also identified more trials showing greater mean weight loss favouring the intervention group than in previous reviews 19, 20, 21. Although 22 trials in this review reported significant between‐group changes, only seven provided actual values for between‐group differences.

The majority of included trials had predominantly Caucasian populations, who were mostly middle‐aged, range 45.7 years 44 to 66.7 years [S22], and were conducted in high‐income countries. This may limit generalizability to other ethnic groups, low‐ or middle‐income countries, and the younger or older. Indeed, those trials including the highest proportions of Black and minority ethnic communities failed to find significant effects on weight/BMI. We may have missed some weight loss trials where overweight was defined as a BMI > 23 kg/m^2^, as seen in Indian and Asian populations. We also note that 18 trials had fewer than 100 participants, so may have had limited power to detect smaller, but clinically important, weight/BMI reductions.

The larger mean baseline BMI in the most successful trials may have led to an overestimation of the effect of the intervention as they comprised participants with potential for greater weight loss. There were limited trials available in those who were severely obese, with no trial having a mean weight of participants > 40 kg/m^2^, limiting generalizability in this context. Trial findings were not always well reported, with some not presenting data at all time points 36 and others not reporting statistical significance of all findings [S21]. In addition, it is difficult to disentangle the effect that intervention participants current antidiabetic medications may have on weight loss.

The interventions were analysed according to intervention type; however, the provision of education varied within individual trials with some delivered one‐to‐one, others to groups, face‐to‐face or via internet or telephone contact. A range of different healthcare professionals were used to deliver the education. This may all have an impact on diabetes self‐care behaviours and adherence to the intervention affecting the outcome measure.

The meta‐analysis has several limitations including a diverse range of types of educational interventions and small numbers of trials for each type of intervention. In addition, many trials had a small number of participants affecting power. We also included trials of any duration in our review. This may have an effect on short‐term results of the intervention on weight/BMI loss, as it is known that weight loss maintenance is problematic for the majority of people after participating in weight loss programmes. Twenty‐eight of the 49 trials identified (57%) had interventions lasting less than 12 months. The results of these interventions maybe of limited significance to clinical practice as the long‐term success of these interventions to weight and BMI change in uncertain.

There was substantial heterogeneity among the included trials, with weight ranging from *I* ^2^ = 0% to *I* ^2^ = 100% and BMI ranging from *I* ^2^ = 0% to *I* ^2^ = 99%, this means that the findings should be interpreted with caution. This heterogeneity was predicted based on the varying trial designs, sociodemographic characteristic of participants, culture and geography of the interventions affecting healthcare provision as well as variations in outcome measures and methods of analysis used (between‐ or within‐group comparisons).

There was a risk of performance and ascertainment bias in nearly all the trials, however, this is difficult to mitigate due to the nature of the behaviour change intervention. It is possible that future trials may alter the conclusions drawn from this review.

### Implications for research, policy and practice

Our review highlights the importance of diet in type 2 diabetes for weight loss. Educational weight loss interventions incorporating low‐calorie meal replacements appear to be the most effective weight loss tools. The trials in our review used meal replacements ranging from once daily to three times daily over 4–52 weeks. Recent evidence suggests prolonged or repeated use of meal replacements may have negative effects on gut microbial diversity, which may be associated with, but not limited to immune‐mediated diseases including inflammatory bowel diseases, irritable bowel syndrome and colorectal cancers [S35–S37]. Our findings provide review‐level evidence to support the design of community‐based programmes using meal replacements, which are increasingly the focus of national initiatives such as that in the UK of a community‐based weight loss programme comprising 3 months of meal replacements and behavioural support for people with type 2 diabetes [7,S38,S39].

Our review also showed that maintenance interventions can be effective in helping prevent weight regain and these need to be incorporated into any weight loss intervention. However, there remains a need to improve lifestyle maintenance interventions.

Further trials are needed in Black and minority ethnic populations [S40,S41], particularly given the greater prevalence of, and poorest outcomes from type 2 diabetes in these groups [S40,S42]. Finally, trials that provide a better understanding of why specific interventions do, or do not work for particular population groups are also required.

## Funding sources

AM is funded by a National Institute for Health Research (NIHR) In Practice Fellowship. The views expressed are those of the author(s) and not necessarily those of the National Health Service (NHS), the NIHR or the Department of Health and Social Care. KK Acknowledges support from NIHR CLAHRC East Midlands and the NIHR Leicester BRC.

## Competing interests

KK was Chair of the NICE PH38 Guidelines on Early Detection and Prevention of Diabetes. He is on the External Reference Group of the National Diabetes Prevention Programme. SW is a member of the Clinical Practice Research Datalink (CPRD) independent scientific advisory committee, statistical advisor to *PLoS Medicine*, academic advisor to Road to Health Ltd, and reports honorarium from AMGEN. There are no other competing interests to declare.

## Supporting information


**Doc. S1.**Additional references.
**Figure S1.**Graphical representation of number of trials within each risk of bias category.
**Figure S2.**Forest plots for (a) weight change and (b) BMI reduction from baseline between groups at the end of the intervention by categories.
**Figure S3.**Forest plots for (a) weight change and (b, c) BMI reduction from baseline between groups at the end of the intervention by categories.
**Figure S4.**Funnel plots for the publication bias in (a) weight change and (b) BMI reduction in the included trials.
**Table S1.**Search strategies.
**Table S2.**Trials with multiple articles identified during review.
**Table S3.**Summary of included trials.
**Table S4.**Risk of bias assessment for included trials.
**Table S5.**Intervention and control group weight and BMI outcomes of all included trials.
