# Supplementary material for: Educational weight loss interventions in obese and overweight adults with type 2 diabetes: a systematic review and meta‐analysis of randomized controlled trials
Source: Diabet Med. 2019 Dec 22;37(4):623–35. doi: 10.1111/dme.14193 (PMC7154644; doi:10.1111/dme.14193)
Supplement: Supplementary file 1 — Doc. S1.Additional references. Figure S1.Graphical representation of number of trials within each risk of bias category. Figure S2.Forest plots for (a) weight change and (b) BMI reduction from baseline between groups at the end of the intervention by categories. Figure S3.Forest plots for (a) weight change and (b, c) BMI reduction from baseline between groups at the end of the intervention by categories. Figure S4.Funnel plots for the publication bias in (a) weight change and (b) BMI reduction in the included trials. Table S1.Search strategies. Table S2.Trials with multiple articles identified during review. Table S3.Summary of included trials. Table S4.Risk of bias assessment for included trials. Table S5.Intervention and control group weight and BMI outcomes of all included trials. [file DME-37-623-s001.zip › dme14193-sup-0009-Supinfo.docx]

**Educational weight loss interventions in obese and overweight adults with type 2 diabetes: a systematic review and meta-analysis of randomized controlled trials**

A. Maula, J. Kai, A. K. Woolley, S. Weng, N. Dhalwani, F. Griffiths, K. Khunti and D. Kendrick

**Doc. S1: Additional references**

| S1. Moncrieft AE, Llabre MM, McCalla JR, Gutt M, Mendez AJ, Gellman MD et al. Effects of a Multicomponent Life-Style Intervention on Weight, Glycemic Control, Depressive Symptoms, and Renal Function in Low-Income, Minority Patients With Type 2 Diabetes: results of the Community Approach to Lifestyle Modification for Diabetes Randomized Controlled Trial. Psychosomatic medicine. 2016;78(7):851‐60. doi:10.1097/PSY.0000000000000348.  S2. Kempf K, Martin S. Autonomous exercise game use improves metabolic control and quality of life in type 2 diabetes patients-a randomized controlled trial. BMC endocrine disorders. 2013;13(1):57.  S3. Li C, Sadraie B, Steckhan N, Kessler C, Stange R, Jeitler M et al. Effects of a one-week fasting therapy in patients with type-2 diabetes mellitus and metabolic syndrome–A randomized controlled explorative study. Experimental and Clinical Endocrinology & Diabetes. 2017;125(09):618-24.  S4. Luley C, Blaik A, Reschke K, Klose S, Westphal S. Weight loss in obese patients with type 2 diabetes: Effects of telemonitoring plus a diet combination–The Active Body Control (ABC) Program. Diabetes research and clinical practice. 2011;91(3):286-92.  S5. Kempf K, Altpeter B, Berger J, Reus O, Fuchs M, Schneider M et al. Efficacy of the Telemedical Lifestyle intervention Program TeLiPro in Advanced Stages of Type 2 Diabetes: A Randomized Controlled Trial. Diabetes Care. 2017;40(7):863-71. doi:https://dx.doi.org/10.2337/dc17-0303.  S6. Campbell EM, Redman S, Moffitt P, Sanson-Fisher RW. The relative effectiveness of educational and behavioral instruction programs for patients with NIDDM: a randomized trial. The Diabetes Educator. 1996;22(4):379-86.  S7. Eakin EG, Reeves MM, Marshall AL, Dunstan DW, Graves N, Healy GN et al. Living Well with Diabetes: a randomized controlled trial of a telephone-delivered intervention for maintenance of weight loss, physical activity and glycaemic control in adults with type 2 diabetes. BMC public health. 2010;10(1):452.  S8. Ash S, Reeves MM, Yeo S, Morrison G, Carey D, Capra S. Effect of intensive dietetic interventions on weight and glycaemic control in overweight men with Type II diabetes: a randomised trial. International journal of obesity and related metabolic disorders. 2003;27(7):797‐802. doi:10.1038/sj.ijo.0802295.  S9. Tay J, Thompson CH, Luscombe-Marsh ND, Wycherley TP, Noakes M, Buckley JD et al. Effects of an energy-restricted low-carbohydrate, high unsaturated fat/low saturated fat diet versus a high-carbohydrate, low-fat diet in type 2 diabetes: a 2-year randomized clinical trial. Diabetes, obesity & metabolism. 2018;20(4):858‐71. doi:10.1111/dom.13164.  S10. Cheyette C. Weight No More: a randomised controlled trial for people with type 2 diabetes on insulin therapy. Practical Diabetes. 2007;24(9):450-6.  S11. Daly ME, Paisey R, Millward B, Eccles C, Williams K, Hammersley S et al. Short‐term effects of severe dietary carbohydrate‐restriction advice in Type 2 diabetes—a randomized controlled trial. Diabetic Medicine. 2006;23(1):15-20.  S12. Adolfsson ET, Walker-Engström M-L, Smide B, Wikblad K. Patient education in type 2 diabetes—a randomized controlled 1-year follow-up study. Diabetes research and clinical practice. 2007;76(3):341-50.  S13. Fritz T, Caidahl K, Krook A, Lundström P, Mashili F, Osler M et al. Effects of Nordic walking on cardiovascular risk factors in overweight individuals with type 2 diabetes, impaired or normal glucose tolerance. Diabetes/metabolism research and reviews. 2013;29(1):25-32.  S14. Guldbrand H, Dizdar B, Bunjaku B, Lindström T, Bachrach-Lindström M, Fredrikson M et al. In type 2 diabetes, randomisation to advice to follow a low-carbohydrate diet transiently improves glycaemic control compared with advice to follow a low-fat diet producing a similar weight loss. Diabetologia. 2012;55(8):2118-27.  S15. Odnoletkova I, Goderis G, Nobels F, Fieuws S, Aertgeerts B, Annemans L et al. Optimizing diabetes control in people with Type 2 diabetes through nurse‐led telecoaching. Diabetic Medicine. 2016;33(6):777-85.  S16. Van Dyck D, De Greef K, Deforche B, Ruige J, Bouckaert J, Tudor-Locke CE et al. The relationship between changes in steps/day and health outcomes after a pedometer-based physical activity intervention with telephone support in type 2 diabetes patients. Health education research. 2013;28(3):539-45.  S17. Vuksan V, Jenkins A, Brissette C, Choleva L, Jovanovski E, Gibbs A et al. Salba-chia (Salvia hispanica L.) in the treatment of overweight and obese patients with type 2 diabetes: A double-blind randomized controlled trial. Nutrition, Metabolism and Cardiovascular Diseases. 2017;27(2):138-46.  S18. Pedersen et al. A portion-control plate was effective for weight loss in obese patients with type 2 diabetes mellitus. ACP Journal Club. 2007;147(3):68-.  S19. Orsama A-L, Lähteenmäki J, Harno K, Kulju M, Wintergerst E, Schachner H et al. Active assistance technology reduces glycosylated hemoglobin and weight in individuals with type 2 diabetes: results of a theory-based randomized trial. Diabetes technology & therapeutics. 2013;15(8):662-9.  S20. Uusitupa M, Laitinen J, Siitonen O, Vanninen E, Pyörälä K. The maintenance of improved metabolic control after intensified diet therapy in recent type 2 diabetes. Diabetes research and clinical practice. 1993;19(3):227-38.  S21. Mitra A, Dewanjee D, Dey B. Mechanistic studies of lifestyle interventions in type 2 diabetes. World journal of diabetes. 2012;3(12):201.  S22. Emerenziani GP, Gallotta MC, Meucci M, Di Luigi L, Migliaccio S, Donini LM et al. Effects of aerobic exercise based upon heart rate at aerobic threshold in obese elderly subjects with type 2 diabetes. International journal of endocrinology. 2015;2015.  S23. Oshakbayev K, Dukenbayeva B, Togizbayeva G, Durmanova A, Gazaliyeva M, Sabir A et al. Weight loss technology for people with treated type 2 diabetes: a randomized controlled trial. Nutrition & metabolism. 2017;14(1):1‐9. doi:10.1186/s12986-017-0163-9.  S24. Krebs JD, Elley CR, Parry-Strong A, Lunt H, Drury PL, Bell DA et al. The Diabetes Excess Weight Loss (DEWL) Trial: a randomised controlled trial of high-protein versus high-carbohydrate diets over 2 years in type 2 diabetes. Diabetologia. 2012;55(4):905‐14. doi:10.1007/s00125-012-2461-0.  S25. Goday A, Bellido D, Sajoux I, Crujeiras A, Burguera B, García-Luna PP et al. Short-term safety, tolerability and efficacy of a very low-calorie-ketogenic diet interventional weight loss program versus hypocaloric diet in patients with type 2 diabetes mellitus. Nutrition & diabetes. 2016;6(9):e230.  S26. Ditschuneit HH, Flechtner-Mors M, Johnson TD, Adler G. Metabolic and weight-loss effects of a long-term dietary intervention in obese patients–. The American journal of clinical nutrition. 1999;69(2):198-204.  S27. Hannum SM, Carson L, Evans EM, Canene KA, Petr EL, Bui L et al. Use of portion‐controlled entrees enhances weight loss in women. Obesity research. 2004;12(3):538-46.  S28. Heymsfield S, Van Mierlo C, Van der Knaap H, Heo M, Frier H. Weight management using a meal replacement strategy: meta and pooling analysis from six studies. International journal of obesity. 2003;27(5):537.  S29. Davis LM, Coleman C, Kiel J, Rampolla J, Hutchisen T, Ford L et al. Efficacy of a meal replacement diet plan compared to a food-based diet plan after a period of weight loss and weight maintenance: a randomized controlled trial. Nutrition Journal. 2010;9(1):11.  S30. McKenzie AL, Hallberg SJ, Creighton BC, Volk BM, Link TM, Abner MK et al. A novel intervention including individualized nutritional recommendations reduces hemoglobin A1c level, medication use, and weight in type 2 diabetes. JMIR diabetes. 2017;2(1):e5.  S31. van Zuuren EJ, Fedorowicz Z, Kuijpers T, Pijl H. Effects of low-carbohydrate-compared with low-fat-diet interventions on metabolic control in people with type 2 diabetes: a systematic review including GRADE assessments. The American journal of clinical nutrition. 2018;108(2):300-31.  S32. Snorgaard O, Poulsen GM, Andersen HK, Astrup A. Systematic review and meta-analysis of dietary carbohydrate restriction in patients with type 2 diabetes. BMJ Open Diabetes Research and Care. 2017;5(1):e000354.  S33. Proper K, Van der Beek A, Hildebrandt V, Twisk J, Van Mechelen W. Short term effect of feedback on fitness and health measurements on self reported appraisal of the stage of change. British journal of sports medicine. 2003;37(6):529-34.  S34. Sherrington A, Newham J, Bell R, Adamson A, McColl E, Araujo‐Soares V. Systematic review and meta‐analysis of internet‐delivered interventions providing personalized feedback for weight loss in overweight and obese adults. obesity reviews. 2016;17(6):541-51.  S35. Zitvogel L, Galluzzi L, Viaud S, Vétizou M, Daillère R, Merad M et al. Cancer and the gut microbiota: an unexpected link. Science translational medicine. 2015;7(271):271ps1-ps1.  S36. Heiman ML, Greenway FL. A healthy gastrointestinal microbiome is dependent on dietary diversity. Molecular metabolism. 2016;5(5):317-20.  S37. Zhang Z, Li D. Thermal processing of food reduces gut microbiota diversity of the host and triggers adaptation of the microbiota: evidence from two vertebrates. Microbiome. 2018;6(1):99.  S38. NHS England. Very low calorie diets part of NHS action to tackle growing obesity and Type 2 diabetes epidemic. 2018. https://www.england.nhs.uk/2018/11/very-low-calorie-diets-part-of-nhs-action-to-tackle-growing-obesity-and-type-2-diabetes-epidemic/. Accessed 20/01/2019.  S39. Astbury NM, Aveyard P, Nickless A, Hood K, Corfield K, Lowe R et al. Doctor Referral of Overweight People to Low Energy total diet replacement Treatment (DROPLET): pragmatic randomised controlled trial. bmj. 2018;362:k3760.  S40. Forouhi N, Merrick D, Goyder E, Ferguson B, Abbas J, Lachowycz K et al. Diabetes prevalence in England, 2001—estimates from an epidemiological model. Diabetic Medicine. 2006;23(2):189-97.  S41. Control CfD, Prevention. National diabetes statistics report: estimates of diabetes and its burden in the United States, 2014. Atlanta, GA: US Department of Health and Human Services. 2014;2014.  S42. Imkampe AK, Gulliford MC. Increasing socio-economic inequality in type 2 diabetes prevalence—repeated cross-sectional surveys in England 1994–2006. The European Journal of Public Health. 2010;21(4):484-90. |
| --- |

**Table S1: Search Strategies**

| **SCOPUS** |
| --- |
| ( ( TITLE-ABS ( "type 2 diabet*" ) ) )  AND  ( ( TITLE-ABS ( obese  OR  obesity  OR  overweight ) ) )  AND  ( ( TITLE-ABS ( educat*  OR  teach*  OR  train*  OR  advice  OR  taught  OR  coach*  OR  learn*  OR  health  AND promot*  OR  knowledge  OR  life  AND style  OR  lifestyle  OR  video  OR  dvd  OR  cd  OR  internet  OR  web  OR  telephon*  OR  phone*  OR  printed  OR  written  OR  material*  OR  booklet*  OR  pamphlet*  OR  leaflet* ) ) )  AND  ( ( TITLE-ABS ( weight  OR  slimming  OR  bmi ) ) )  AND  ( ( TITLE-ABS ( random*  OR  trial ) ) ) |
| **Embase** |
| 1. exp Diabetes Mellitus, Type 2/  2. (MODY or NIDDM or T2DM or T2D).ti,ab.  3. (non insulin$ depend$ or noninsulin$ depend$ or noninsulin?depend$ or non insulin?depend$).ti,ab.  4. ((typ? 2 or typ? II or typ?2 or typ?II) adj3 diabet$).ti,ab.  5. (((late or adult$ or matur$ or slow or stabl$) adj3 onset) and diabet$).ti,ab.  6. 1 or 2 or 3 or 4 or 5  7. exp Obesity/  8. (Obes$ or overweight$).ti,ab.  9. 7 or 8  10. Education/  11. exp Health Promotion/  12. exp Self Care/  13. exp Life Style/  14. exp Consumer Health Information/  15. exp Patient Education as Topic/  16. exp Health Education/  17. (educat$ or instruct$ or teach$ or train$ information$ or program$ or session$ or intervention$ or advice or taught or coach$ or learn$ or behav$ or self monitor$ or self manag$ or self car$ or health promot$ or knowledge or life style or life?style or lifestyle or video or DVD or CD or internet or web or telephon$ or phone$ or printed or written or material$ or booklet$ or pamphlet$ or leaflet$ or poster or social support or self help or counsel$).ti,ab.  18. or/10-17  19. body weight changes/ or weight loss/  20. weight.ti,ab.  21. slimming.ti,ab.  22. body mass index/  23. (body mass index or BMI).ti,ab.  24. 19 or 20 or 21 or 22 or 23  25. 18 and 24  26. crossover procedure.sh.  27. double-blind procedure.sh.  28. single-blind procedure.sh.  29. (crossover$ or cross over$).tw.  30. placebo$.tw.  31. (doubl$ adj blind$).tw.  32. allocat$.tw.  33. trial.ti.  34. randomized controlled trial.sh.  35. random$.tw.  36. or/26-35  37. exp animal/ or exp invertebrate/ or animal experiment/ or animal model/ or animal tissue/ or animal cell/ or nonhuman/  38. human/ or normal human/  39. 37 and 38  40. 37 not 39  41. 36 not 40  42. 6 and 9 and 25 and 41 |
| **CINAHL** |
| type 2 diabetes or type 2 diabetes mellitus or t2dm  AND overweight or obesity or obese  AND education  AND weight loss or weight reduction or lose weight |
| **The Cochrane Central Register of Controlled Trials (CENTRAL)** |
| #1 MeSH descriptor: [Diabetes Mellitus, Type 2] explode all trees  #2 (MODY or NIDDM or T2DM or T2D):ti,ab,kw  #3 (non insulin* depend$ or noninsulin* depend* or non insulin*depend*):ti,ab,kw  #4 ((typ* 2 or typ* II) and diabet*):ti,ab,kw  #5 (((late or adult* or matur* or slow or stabl*) and onset) and diabet*):ti,ab,kw  #6 {OR #1-#5}  #7 MeSH descriptor: [Obesity] explode all trees  #8 (Obes* or overweight*):ti,ab,kw  #9 {OR #7-#8}  #10 (educat* or instruct* or teach* or counsel* or train* information* or program* or session* or intervention* or advice or taught or coach* or learn* or behav* or self monitor* or self manag* or self car* or health promot* or knowledge or life style or life?style or lifestyle or video or DVD or CD or internet or web or telephon* or phone* or printed or written or material* or booklet* or pamphlet* or leaflet*):ti,ab,kw  #11 MeSH descriptor: [Education] this term only  #12 MeSH descriptor: [Health Promotion] explode all trees  #13 MeSH descriptor: [Self Care] explode all trees  #14 MeSH descriptor: [Life Style] explode all trees  #15 MeSH descriptor: [Consumer Health Information] explode all trees  #16 MeSH descriptor: [Patient Education as Topic] explode all trees  #17 MeSH descriptor: [Patient Education Handout] explode all trees  #18 MeSH descriptor: [Health Education] explode all trees  #19 {OR #10-#18}  #20 MeSH descriptor: [Body Weight Changes] explode all trees  #21 MeSH descriptor: [Weight Loss] explode all trees  #22 (weight or slimming):ti,ab,kw  #23 MeSH descriptor: [Body Mass Index] explode all trees  #24 (body mass index or bmi):ti,ab,kw  #25  #26 #6 and #9 and #19 and #25 |
| **MEDLINE** |
| 1. exp Diabetes Mellitus, Type 2/  2. (MODY or NIDDM or T2DM or T2D).ti,ab.  3. (non insulin$ depend$ or noninsulin$ depend$ or noninsulin?depend$ or non insulin?depend$).ti,ab.  4. ((typ? 2 or typ? II or typ?2 or typ?II) adj3 diabet$).ti,ab.  5. (((late or adult$ or matur$ or slow or stabl$) adj3 onset) and diabet$).ti,ab.  6. 1 or 2 or 3 or 4 or 5  7. exp Obesity/  8. (Obes$ or overweight$).ti,ab.  9. 7 or 8  10. Education/  11. exp Health Promotion/  12. exp Self Care/  13. exp Life Style/  14. exp Consumer Health Information/  15. exp Patient Education as Topic/  16. exp Patient Education Handout/  17. exp Health Education/  18. (educat$ or instruct$ or teach$ or train$ information$ or program$ or session$ or intervention$ or advice or taught or coach$ or learn$ or behav$ or self monitor$ or self manag$ or self car$ or health promot$ or knowledge or life style or life?style or lifestyle or video or DVD or CD or internet or web or telephon$ or phone$ or printed or written or material$ or booklet$ or pamphlet$ or leaflet$ or poster or social support or self help or counsel$).ti,ab.  19. or/10-18  20. body weight changes/ or weight loss/  21. weight.ti,ab.  22. slimming.ti,ab.  23. body mass index/  24. (body mass index or BMI).ti,ab.  25. 20 or 21 or 22 or 23 or 24  26. 19 and 25  27. randomized controlled trial.pt.  28. controlled clinical trial.pt.  29. randomized.ab.  30. placebo.ab.  31. clinical trials as topic.sh.  32. randomly.ab.  33. trial.ti.  34. 27 or 28 or 29 or 30 or 31 or 32 or 33  35. exp animals/ not humans.sh.  36. 34 not 35  37. 6 and 9 and 26 and 36 |

**Table S2: Trials with multiple articles identified during review**

| **Look Ahead trial papers** |
| --- |
| 1. Association of Weight Loss Maintenance and Weight Regain on 4-Year Changes in CVD Risk Factors: the Action for Health in Diabetes (Look AHEAD) Clinical Trial. Diabetes Care. 2016;39(8):1345-55. doi:10.2337/dc16-0509.  2. Albu JB, Heilbronn LK, Kelley DE, Smith SR, Azuma K, Berk ES et al. Metabolic changes follo Association of Weight Loss Maintenance and Weight Regain on 4-Year Changes in CVD Risk Factors: the Action for Health in Diabetes (Look AHEAD) Clinical Trial a 1-year diet and exercise intervention in patients with type 2 diabetes. Diabetes 2010 doi:10.2337/db09-1239.  3. Espeland MA, Glick HA, Bertoni A, Brancati FL, Bray GA, Clark JM et al. Impact of an intensive lifestyle intervention on use and cost of medical services among overweight and obese adults with type 2 diabetes: The action for health in diabetes. Diabetes Care. 2014;37(9):2548-56. doi:http://dx.doi.org/10.2337/dc14-0093.  4. Gallagher D, Heshka S, Kelley DE, Thornton J, Boxt L, Pi-Sunyer FX et al. Changes in adipose tissue depots and metabolic markers following a 1-year diet and exercise intervention in overweight and obese patients with type 2 diabetes. Diabetes care 2014 doi:10.2337/dc14-1585.  5. Neiberg RH, Wing RR, Bray GA, Reboussin DM, Rickman AD, Johnson KC et al. Patterns of weight change associated with long-term weight change and cardiovascular disease risk factors in the Look AHEAD Study. Obesity (19307381). 2012;20(10):2048-56. doi:10.1038/oby.2012.33.  6. Padwal R. An intensive lifestyle intervention reduced weight and cardiovascular disease risk factors in obese participants with type 2 diabetes at 1 year in an interim analysis. Evidence Based Medicine. 2007;12(6):180-.  7. Pi-Sunyer X. The Look AHEAD Trial: A Review and Discussion of Its Outcomes. Current Nutrition Reports. 2014;3(4):387-91. doi:http://dx.doi.org/10.1007/s13668-014-0099-x.  8. Pi-Sunyer X, Blackburn G, Brancati FL, Bray GA, Bright R, Clark JM et al. Reduction in weight and cardiovascular disease risk factors in individuals with type 2 diabetes: one-year results of the look AHEAD trial. Diabetes care 2007 doi:10.2337/dc07-0048.  9. Raynor HA, Jeffery RW, Ruggiero AM, Clark JM, Delahanty LM. Weight loss strategies associated with BMI in overweight adults with type 2 diabetes at entry into the Look AHEAD (Action for Health in Diabetes) trial. Diabetes care 2008 doi:10.2337/dc07-2295.  10. Ryan DH, Espeland MA, Foster GD, Haffner SM, Hubbard VS, Johnson KC et al. Look AHEAD (Action for Health in Diabetes): design and methods for a clinical trial of weight loss for the prevention of cardiovascular disease in type 2 diabetes. Control Clin Trials. 2003;24(5):610-28.  11. Unick JL, Beavers D, Jakicic JM, Kitabchi AE, Knowler WC, Wadden TA et al. Effectiveness of lifestyle interventions for individuals with severe obesity and type 2 diabetes: results from the Look AHEAD trial. Diabetes Care. 2011;34(10):2152-7. doi:10.2337/dc11-0874.  12. Unick JL, Hogan PE, Neiberg RH, Cheskin LJ, Dutton GR, Evans-Hudnall G et al. Evaluation of early weight loss thresholds for identifying nonresponders to an intensive lifestyle intervention. Obesity (Silver Spring, Md) 2014 doi:10.1002/oby.20777.  13. Unick JL, Neiberg RH, Hogan PE, Cheskin LJ, Dutton GR, Jeffery R et al. Weight change in the first 2 months of a lifestyle intervention predicts weight changes 8 years later. Obesity (Silver Spring, Md) 2015 doi:10.1002/oby.21112.  14. Waddell FM, Neiberg RH, Wing RR, Clark JM, Delahanty LM, Hill JO et al. Four-year weight losses in the Look AHEAD study: factors associated with long-term success. Obesity (19307381). 2011;19(10):1987-98. doi:10.1038/oby.2011.230.  15. Wadden TA. Eight-year weight losses with an intensive lifestyle intervention: The look AHEAD study. Obesity (Silver Spring). 2014;22(1):5-13. doi:10.1002/oby.20662.  16. Williamson DA, Rejeski J, Lang W, Van Dorsten B, Fabricatore AN, Toledo K. Impact of a weight management program on health-related quality of life in overweight adults with type 2 diabetes. Arch Intern Med. 2009;169(2):163-71. doi:10.1001/archinternmed.2008.544.  17. Wing RR, Bahnson JL, Bray GA, Clark JM, Coday M, Egan C et al. Long-term effects of a lifestyle intervention on weight and cardiovascular risk factors in individuals with type 2 diabetes mellitus: Four-year results of the look AHEAD trial. Arch Intern Med. 2010;170(17):1566-75. doi:http://dx.doi.org/10.1001/archinternmed.2010.334.  18. Gregg EW, Jakicic JM, Blackburn G, Bloomquist P, Bray GA, Clark JM et al. Association of the magnitude of weight loss and changes in physical fitness with long-term cardiovascular disease outcomes in overweight or obese people with type 2 diabetes: a post-hoc analysis of the Look AHEAD randomised clinical trial. The lancet Diabetes & endocrinology. 2016;4(11):913‐21. doi:10.1016/S2213-8587(16)30162-0.  19. Belalcazar, L.M., Reboussin, D.M., Haffner, S.M., Hoogeveen, R.C., Kriska, A.M., Schwenke, D.C., Tracy, R.P., Pi-Sunyer, F.X., Ballantyne, C.M. and Look AHEAD Research Group, 2010. A 1-year lifestyle intervention for weight loss in individuals with type 2 diabetes reduces high C-reactive protein levels and identifies metabolic predictors of change: from the Look AHEAD (Action for Health in Diabetes) study. Diabetes care, 33(11), pp.2297-2303.  20. Bray, G., Wagenknecht, L., Walkup, M., Heshka, S., Hill, J., Hubbard, V., Johnson, K., Kahn, S., Nathan, D. and Pownall, H., 2012. Effect of age on loss and regain of lean body mass in type 2 diabetes: four-year results from the look ahead dxa substudy: 156 accepted oral. Obesity facts, 5.  21. Chyun, D., 2008. An intensive lifestyle intervention reduced weight and cardiovascular disease risk factors in overweight and obese people with type 2 diabetesCommentary. Evidence-based nursing, 11(1), pp.16-16.  22. Espeland, M.A., Bray, G.A., Neiberg, R., Rejeski, W.J., Knowler, W.C., Lang, W., Cheskin, L.J., Williamson, D., Lewis, C.B., Wing, R. and Look Ahead Study Group, 2009. Describing patterns of weight changes using principal components analysis: results from the Action for Health in Diabetes (Look AHEAD) research group. Annals of epidemiology, 19(10), pp.701-710.  23. Faulconbridge, L.F., Wadden, T.A., Rubin, R.R., Wing, R.R., Walkup, M.P., Fabricatore, A.N., Coday, M., Van Dorsten, B., Mount, D.L., Ewing, L.J. and Look AHEAD Research Group, 2012. One‐year changes in symptoms of depression and weight in overweight/obese individuals with type 2 diabetes in the look AHEAD study. Obesity, 20(4), pp.783-793.  24. Gibbs, B.B., Brancati, F.L., Chen, H., Coday, M., Jakicic, J.M., Lewis, C.E., Stewart, K.J. and Clark, J.M., 2011, November. Combined Effects of Fitness and Physical Activity Beyond Weight Loss on Fasting Glucose and HbA1c in Adults with Type 2 Diabetes. In OBESITY (Vol. 19, pp. S122-S122). 75 VARICK ST, 9TH FLR, NEW YORK, NY 10013-1917 USA: NATURE PUBLISHING GROUP.  25. Johnston, C.A., Moreno, J.P. and Foreyt, J.P., 2014. Cardiovascular effects of intensive lifestyle intervention in type 2 diabetes. Current atherosclerosis reports, 16(12), p.457.  26. Pacanowski, C.R., Linde, J.A., Faulconbridge, L.F., Coday, M., Safford, M.M., Chen, H., Yanovski, S.Z., Ewing, L.J., Wing, R. and Jeffery, R.W., 2018. Psychological status and weight variability over eight years: Results from Look AHEAD. Health Psychology, 37(3), p.238.  27. Raynor, H.A., Anderson, A.M., Miller, G.D., Reeves, R., Delahanty, L.M., Vitolins, M.Z., Harper, P., Mobley, C., Konersman, K., Mayer-Davis, E. and Brancati, F.L., 2015. Partial meal replacement plan and quality of the diet at 1 year: Action for Health in Diabetes (Look AHEAD) Trial. Journal of the Academy of Nutrition and Dietetics, 115(5), pp.731-742.  28. Wadden, T.A., Neiberg, R.H., Wing, R.R., Clark, J.M., Delahanty, L.M., Hill, J.O., Krakoff, J., Otto, A., Ryan, D.H., Vitolins, M.Z. and Look AHEAD Research Group, 2011. Four‐year weight losses in the Look AHEAD study: factors associated with long‐term success. Obesity, 19(10), pp.1987-1998.  29. Rejeski, W.J., Ip, E.H., Bertoni, A.G., Bray, G.A., Evans, G., Gregg, E.W. and Zhang, Q., 2012. Lifestyle change and mobility in obese adults with type 2 diabetes. New England Journal of Medicine, 366(13), pp.1209-1217.  30. Unick, J.L., Beavers, D., Bond, D.S., Clark, J.M., Jakicic, J.M., Kitabchi, A.E., Knowler, W.C., Wadden, T.A., Wagenknecht, L.E., Wing, R.R. and Look AHEAD Research Group, 2013. The long-term effectiveness of a lifestyle intervention in severely obese individuals. The American journal of medicine, 126(3), pp.236-242.  31. Unick, J.L., Beavers, D., Jakicic, J.M., Kitabchi, A., Knowler, W.C., Wadden, T.A. and Wing, R.R., 2010, October. The Effectiveness of a Lifestyle Weight Loss Intervention in the Severely Obese: Results From the Look AHEAD Trial. In OBESITY (Vol. 18, pp. S68-S68). 75 VARICK ST, 9TH FLR, NEW YORK, NY 10013-1917 USA: NATURE PUBLISHING GROUP.  32. Westman, E.C., 2014. In overweight or obese patients with diabetes, a lifestyle intervention increased weight loss at 8 years. Annals of internal medicine, 160(12), pp.JC4-JC4. |
| **ICAN1 trial Papers** |
| 1. Gurka MJ, Wolf AM, Conaway MR, Crowther JQ, Nodler JL, Bovbjerg VE. Lifestyle intervention in obese patients with type 2 diabetes: Impact of the patient's educational background. Obesity (Silver Spring). 2006;14(6):1085-92.  2. Wolf AM, Siadaty M, Yaeger B, Conaway MR, Crowther JQ, Nadler JL et al. Effects of Lifestyle Intervention on Health Care Costs: Improving Control with Activity and Nutrition (ICAN). J Am Diet Assoc. 2007;107(8):1365-73. doi:10.1016/j.jada.2007.05.015. |
| **DiRECT trial** |
| 1. Lean, M.E., Leslie, W.S., Barnes, A.C., Brosnahan, N., Thom, G., McCombie, L., Peters, C., Zhyzhneuskaya, S., Al-Mrabeh, A., Hollingsworth, K.G. and Rodrigues, A.M., 2019. Durability of a primary care-led weight-management intervention for remission of type 2 diabetes: 2-year results of the DiRECT open-label, cluster-randomised trial. The Lancet Diabetes & Endocrinology, 7(5), pp.344-355. |
| **Tay et al** |
| 1. Tay, J., Luscombe-Marsh, N.D., Thompson, C.H., Noakes, M., Buckley, J.D., Wittert, G.A., Yancy Jr, W.S. and Brinkworth, G.D., 2015. Comparison of low-and high-carbohydrate diets for type 2 diabetes management: a randomized trial. The American journal of clinical nutrition, 102(4), pp.780-790. |
| **Living well with Diabetes** |
| 1. Eakin, E.G., Winkler, E.A., Dunstan, D.W., Healy, G.N., Owen, N., Marshall, A.M., Graves, N. and Reeves, M.M., 2014. Living well with diabetes: 24-month outcomes from a randomized trial of telephone-delivered weight loss and physical activity intervention to improve glycemic control. Diabetes Care, 37(8), pp.2177-2185.  2. Eakin, E.G., Reeves, M.M., Winkler, E., Healy, G.N., Dunstan, D.W., Owen, N., Marshal, A.M. and Wilkie, K.C., 2013. Six-month outcomes from living well with diabetes: a randomized trial of a telephone-delivered weight loss and physical activity intervention to improve glycemic control. Annals of Behavioral Medicine, 46(2), pp.193-203. |

**Table S3 Summary of Included Trials**

| **Trial/ author(s)/**  **Country** | **Participant Characteristics** | **Intervention** | **Outcome Measures & Results** |
| --- | --- | --- | --- |
| **Education alone** | | | |
| **Weight or BMI reduction <5% at end of intervention** | | | |
| Patient education in type 2 diabetes: a randomized controlled 1-year follow-up study  Adolfsson, E. T.et al 2007  Sweden[1] | **N= 101:** Intervention group (IG): 50, control group (CG):51  **Age** (years) mean (SD): IG: 62.4 (8.9), CG: 63.7 (9.0)  **BMI** (kg/m2) Mean (SD): IG: 30.4 (4.3), CG: 29.6 (3.3)  **Female** **sex** (%): IG: 43% CG: 39% | **Intervention length:** 1 year  **Intervention aim:** To determine the effect of empowerment group education on confidence relating to diabetes knowledge, self-efficacy, satisfaction with daily life, BMI and HBA1c when compared to patients receiving routine diabetes care.  **IG:** Empowerment group education  **CG:** Routine diabetes care  **Education delivery:** Group | **Outcomes measured at:** 1 year  **Outcome measures:**  **Primary:** Glycaemic control  **Secondary**: Participant’s confidence in diabetes knowledge, self-efficacy, satisfaction with daily life, BMI |
| Weight No More: a randomised controlled trial for people with type 2 diabetes on insulin therapy  Cheyette, C. et al 2007 UK[2] | **N=49**: IG:29, CG:20  **Age** (years) mean (SD): IG: 56.7 (9.7), CG: 58 (10.7)  **BMI** (kg/m2) Mean (SD): IG: 34.1 (4.7), CG: 31.7 (5.4)  **Weight** (kg) Mean (SD): IG: 97.2 (15.1), CG: 90.8 (14.3) | **Intervention length**: 4 months (8-month maintenance intervention)  **Intervention aim:** To investigate whether a dietician led weight management programme ‘Weight No More’ was effective at helping participants on insulin to lose weight in the short and long term.  **IG:** Weight No More programme  **CG:** Standard diabetes care  **Education delivery**: Group | **Outcomes measured at:** 4,6 and 12 months  **Outcome measures:**  **Primary:** Weight and weight change from baseline  **Secondary:** HbA1c, insulin dose and Audit of Diabetes- Dependent Quality of Life (ADDQoL) (2 specific questions in this tool measuring present QoL) |
| Improving Control with Activity and Nutrition (ICAN)  Wolf A.M. et al 2004 USA[3] | **N=147**: Intervention group (IG): 74, Control group (CG): 73  **Age** (years) mean (SD): IG: 53.3(8.6), CG: 53.4(8.0)  **Body mass index** (BMI) (kg/m^2^): Mean (SD): IG:37.6(7.7), CG: 37.5 (6.4)  **Female sex** (%): IG: 62% CG: 58%  **Ethnicity**: 74% of CG and 85% of IG were Caucasian. Other ethnicities not specified. | **Intervention length:** 12 months  **Intervention aim:** Assessing the effectiveness of a registered dietician (RD) led approach to lifestyle change compared to usual care. Aiming to achieve 5% -10% weight loss.  **IG**: Lifestyle case management  **CG:** Usual care  **Education delivery**: Group and individual | **Outcomes measured at:** 4, 6, 8 and 12 months  **Outcome measures**:  **Primary**: Weight and weight change from baseline and waist circumference  **Secondary**: Hba1c, lipid levels, use of prescription medications, and health-related quality of life (HRQOL) (Medical Outcomes Study Short Form-36 [SF-36]) |
| Family Partners in Lifestyle Support (PALS): Family-Based  Weight Loss for African American Adults with Type 2  Diabetes  Samuel-Hodge et al 2017 USA[4] | **N = 53:** IG: 36, CG:17  **Age** (years) Mean: IG: 55, CG: 53  **BMI** (kg/m2) Mean: IG: 38.1, CG: 37.1  **Female** **sex** (%): IG:75%, CG: 72%  **Ethnicity:** 100% African -American | **Intervention length:** 20 weeks  **Intervention aim:** To establish and determine whether a family based behavioural weight loss programme, where overweight and obese diabetics were partnered with family members without diabetes but who were also the same age, BMI with similar PA levels, was effective at achieving weight loss.  **IG**: Special intervention: intervention participants paired with their family member were asked to attend weekly education sessions, physical activity (PA), and try new recipes. Calorie intake was personalised with 180-300minutes/week of moderate intensity PA.  **CG:** Delayed intervention control group: received a single newsletter during intervention. At the end of the intervention, the control group were offered a 6-week programme of the special intervention.  **Education delivery:** Group | **Outcomes measured at:** Weekly weights, 20 weeks  **Outcome measures:**  **Primary**: Change in weight from baseline  **Secondary:** Change in weight in family partners from baseline, HBA1c and PA change from baseline, diet behaviours, change in BP |
| **Weight or BMI reduction >5% at end of intervention** | | | |
| The relative effectiveness of educational and behavioural instruction programs for patients with NIDDM: a randomized trial  Campbell, E. M. et al 1996  Australia[5] | **N= 241**: IG1: 57, IG2:66, IG3: 59, CG: 59  **Age** (years) Mean (SD): IG1: 56.8 (1.5), IG2: 58.4 (1.4), IG3: 60.9 (1.4), CG: 58.2 (1.3)  **BMI** (kg/m2) Mean (SD): IG1: 31.4 (0.9), IG2: 30.0 (0.6), IG3:29.7 (0.7), CG: 31.5 (0.9) | **Intervention length:** 12 months  **Intervention aim:** To assess the effectiveness of several different diabetes educational programs teaching self-care for non-insulin dependent diabetics (dietary advice, exercise, urine/blood glucose monitoring, medication, foot care)  **IG1**: Individual education  **IG2**: Group education  **IG3**: A behavioural program  **CG**: A minimal instruction program covering topics in less detail than in other groups  **Education delivery:** Group and individual | **Outcomes measured at:** 3,6 and 12 months  **Outcome measures: Primary not specified**. Blood glucose, hba1c, diabetes treatment, change in BMI from baseline, blood lipids, systolic and diastolic blood pressure, smoking, diabetes knowledge, patient satisfaction |
| Metabolic impact of education in NIDDM.  D'Eramo-Melkus, G.A.et al 1992 USA[6] | **N= 82**: IG1: 28, IG2: 26, CG: 28  **Age** (years) mean (SD): All: 55.6 (8.05)  **Weight** (pounds) mean (SD): All: 209.65 (28.00) | **Intervention length**: 12 weeks (12-week maintenance intervention)  **Intervention aim:** To assess effect of a model programme of diabetes education and weight loss on diabetes control and weight reduction.  Prior to group allocation all participants took part in a 1-week minimal skills educational intervention.  **IG 1:** Diabetes education and weight-reduction intervention  **IG 2:** Group education intervention with counselling  **CG**: Usual care  **Education delivery:** Group and individual | **Outcomes measured at:** 3 and 6 months  **Outcome measures:**  **Primary:** Weight, fasting blood glucose, and HbA1c  **Secondary:** Diabetes knowledge, skills, and attitudes were also obtained |
| Mechanistic studies of lifestyle interventions in type 2 diabetes.  Mitra, A. et al 2012 India[7] | **N=60**: IG: 30, CG: 30  **Age** group (years): N (%): 40-50: 15 (25), 50-60: 28 (46.66), > 60: 17 (28.33)  **Female** **sex**: All: 28%  **BMI**: All: 27 (0.92) | **Intervention length:**1 year  **Intervention aim:** To determine the effect of lifestyle interventions in type II DM  **IG**: Lifestyle counselling and education  **CG:** Usual care  **Education delivery:** Not clear | **Outcomes measured at:** 12 months  **Outcome measures: Primary not specified:** HbA1c, fasting plasma glucose level (FPG), postprandial plasma glucose Level (PPG), total cholesterol (TC), triglyceride (TG), high-density lipoproteins (HDL), low-density lipoproteins (LDL) levels and BMI |
| Active assistance technology reduces glycosylated hemoglobin and weight in individuals with type 2 diabetes: results of a theory-based randomized trial Orsama et al 2013 Finland[8] | **N = 48:** IG:24, CG:24  **Age** (years) Mean (SD): IG: 62.3 (6.5), CG: 61.5 (9.1)  **BMI** (kg/m2) Mean (SD): IG: 30.7 (4.5), CG: 33.5 (8.0)  **Female** **sex** (%): IG:46%, CG: 54% | **Intervention length:** 10 months.  **Intervention aim:** To determine if a mobile telephone based remote patient reporting system was effective at improving self-management and health status.  **IG**: Active assistance technology: individual targets were devised, and a personal care plan generated. Pedometers, in specific cases glucose meters, mobile phones with an app (‘monica’) were provided so participants could record their health parameters (BP, weight, PA and in specific cases blood glucose values) at home. Feedback was automatically generated by a decision support system directly integrated with participants health records to help self-care practice. Study nurses went through all the participant entered information weekly and made contact if required.  **CG:** Usual care  **Education delivery:** Individual | **Outcomes measured at:** 10 months  **Outcome measures:**  **Primary**: Change in HBA1c (% units) and systolic and diastolic blood pressure (mm Hg)  **Secondary:** Change in weight from baseline |
| Community Diabetes Education (CoDE) for uninsured Mexican Americans: A randomized controlled trial of a  culturally tailored diabetes education and management  program led by a community health worker  Prezio et al 2013 USA[9] | **N= 180**: IG: 90, CG: 90  **Age** (years) mean (SD): IG: 47.9 (10.00), CG: 45.7 (10.69)  **BMI** (kg/m2) Mean (SD): IG:32.7 (7.77) CG:33.9 (8.24)  **Female** **sex** (%): All 64%, IG:66.7%, CG:54.4%  **Ethnicity**: All: Mexican Americans (78%), African Americans (15%), Caucasians (6%), and Asians (1%). | **Intervention length:** 12- months  **Intervention aim:** To determine the effect of a diabetes education programme guided by a community health worker(CHW), culturally tailored for Mexican Americans without health insurance on HbA1c, BP, BMI and lipids.  **IG:** Participants took part in the CoDE programme, comprising 3 educational modules. Sessions covered areas advised by the American diabetes association including monitoring blood glucose, diet, PA and diabetic complications. Sessions were either in English or Spanish. Following this they were required to meet with the community health worker to reinforce knowledge and skills.  **CG:** Received usual medical care  **Education delivery**: Individual | **Outcomes measured at:** 3, 6, 9 and 12 months  **Outcome measures:**  **Primary:** HbA1c  **Secondary:** Blood pressure, BMI and lipid status |
| **Education and counselling** | | | |
| **Weight or BMI reduction <5% at end of intervention** | | | |
| Continuous glucose monitoring counselling improves physical activity behaviours of individuals with type 2 diabetes: A randomized clinical trial  Allen, N. A. et al 2008 USA[10] | **N = 52:** IG: 27, CG: 25  **Age** (years) mean (3SD): 57.0 (13.5)  **BMI** (kg/m2) means +/- 3SD: 35.0 (5.9)  **Female sex** (%): All: 52%  **Ethnicity:** All: 90% Caucasian | **Intervention length:** 8 weeks  **Intervention aim:** To assess the effects of a counselling intervention utilising continuous glucose monitoring system (CGMS) to change PA levels and behaviour.  **IG:** Counselling intervention using CGMS technology  **CG:** Individualized diabetes education based on the International Diabetes Centre curriculum  **Educational delivery:** Individual | **Outcomes measured at:** 8 weeks  **Outcome measures: Primary not specified:** Physical activity self-efficacy, physical activity levels, glycaemic control, blood pressure (BP), HbA1c, and BMI |
| Clinic-Based Support to Help Overweight Patients  With Type 2 Diabetes Increase Physical Activity  and Lose Weight  Christian, J.G.et al 2008 USA[11] | **N = 310:** IG: 155, CG: 155  **Age** (years) means (SD): IG: 53.0 (11.25), CG:53.4 (10.70)  **Weight** (pounds) mean (SD): IG:207.0 (47.3), CG: 200.2 (44.7)  **BMI** (kg/m2) means (SD): IG: 35.4 (6.62), CG: 34.8 (7.11)  **Ethnicity**: All: 100% Hispanic/Latino population | **Intervention length:** 12 months  **Intervention aim:** To assess effect of brief physician provided tailored healthy lifestyle counselling to patients with type 2 diabetes mellitus during usual care visits  **IG:** Brief health lifestyle counselling  **CG:** Usual care  **Educational delivery:** Individual | **Outcomes measured at:**12 months  **Outcomes:**  **Primary**: Change in weight from baseline,  **Secondary:** Change in physical activity, changes in lipid and HbA1c levels |
| Randomized controlled trial of a nationally available weight control program tailored for adults with type 2 diabetes  O'Neil, P. M. et al 2016 USA[12] | **N= 563:** IG: 279, CG: 284  **Age (years)**: 18 to 70  **Female** **sex** (%): IG: 72%, CG: 70%  **Ethnicity** %: African-American: IG: 36, CG: 38, Caucasian: IG: 46, CG: 44, Hispanic: IG: 10, CG:11, Other: IG: 8, CG: 7 | **Intervention length:** 12 months  **Aims:** To assess the effects of the commercially available weight watchers (WW) programme delivered by weight watchers staff combined with email and telephone contact with a certified diabetes educator compared against standard care on glycaemic control and weight.  **IG:** Weight Watchers program and counselling  **CG:** Standard care  **Education delivery**: Group and individual | **Outcomes measured at:** 3,6,9 and 12 months  **Outcome measures**:  **primary:** HbA1c,  **secondary:** Weight, waist circumference, BP |
| **Education and low-calorie diet** | | | |
| **Low carbohydrate** | | | |
| **Weight or BMI reduction <5% at end of intervention** | | | |
| One-year comparison of a high-monounsaturated fat diet with a high-carbohydrate diet in type 2 diabetes Brehm, B. J. et al 2009 USA[13] | **N = 95 :** IG: 52, CG: 43  **Age** (years) Mean (SEM): ALL: 56.5 (0.8)  **Weight** (Kg) Mean (SD): IG: 102.1 (2.0), CG: 103.7 (2.8)  **BMI**(kg/m2) Mean (SD): ALL: 35.9 (0.3)  **Female** **sex** (%): IG: 67%, CG: 65%  **Ethnicity**: ALL: Caucasian (74%), African Americans (26%) | **Intervention length:** 1 year  **Intervention aim:** To compare a high monounsaturated fatty acid (MUFA) diet to a high carbohydrate (CHO) diet on anthropometric and metabolic parameters.  Both groups received an individual diet plan containing a 200-300 calorie deficit. Prescriptions adjusted during study dependent on weight loss. Encouragement to exercise. Weekly meetings with dietician for 2 months, biweekly till month 4 followed by monthly till end of intervention, alternating individual and group sessions. Kept food diaries. Pedometers to monitor step counts.  **IG**: High MUFA diet - 45% carbohydrate, 15% protein, 40% fat (20% MUFA)  **CG:** High CHO diet - 60% CHO, 15% protein, 25% fat  **Education delivery:** Individual and group | **Outcomes measured at:** 4,8 12 and 28 months  **Outcome measures: Primary not specified:**  Weight loss, bodyfat, waist circumference, diastolic blood pressure, lipids, glycaemic control |
| Short-term effects of severe dietary carbohydrate-restriction advice in Type 2 diabetes--a randomized controlled trial  Daly, M. E. et al 2005 UK[14] | **N= 102**: IG: 51, CG:51  **Age** (years) mean (SD): IG: 58.2 (1.55), CG: 59.1 (1.48)  **BMI** (kg/m2) Mean (SD): IG: 35.4 (0.70), CG: 36.7(1.26)  **Weight** (kg) Mean(SD): IG: 101.6(1.84), CG: 102.3 (2.49) | **Intervention length:** 3 months  **Intervention aim:** To investigate the effects of a 3-month programme of dietary advice restricting carbohydrate intake versus reduced-portion, low-fat meals.  **IG:** Low carbohydrate group  **CG:** Healthy eating group  **Education delivery**: Group and individual | **Outcomes measured at:** 3 months  **Outcome measures:**  **Primary:** Weight change from baseline, HbA1c, total cholesterol: HDL ratio and triacylglycerol concentrations  **Secondary:** Change in blood pressure (BP) and dietary quality |
| Comparative Study of the Effects of a 1-Year Dietary Intervention of a Low- Carbohydrate Diet Versus a Low-fat Diet on Weight and Glycemic Control in Type 2 Diabetes Davis, N.J. et al 2009  USA[15] | **N= 105 :** IG: 55, CG: 50  **Age** (years) Mean (SD): IG: 54 (6), CG: 53 (7)  **Weight** (Kg) Mean (SD): IG: 93.6 (18), CG: 101 (1.4)  **BMI** (kg/m2) Mean (SD): IG: 35 (6), CG: 37 (6)  **Female** **sex** (%): IG: 82%, CG: 74%  **Ethnicity** : IG : Black 62%, Hispanic 15%, White 15%, Asian 4%, Other 4%  CG : Black 66%, Hispanic 18%, White 14%, Asain 2%, Other 0% | **Intervention length:** 12 months  **Aims:** To compare the effects of low fat or low carbohydrate diets on weight loss and HbA1c in type II diabetics.  Individuals entered into a 3-4 week pre-randomization protocol. Those that completed this period were then randomized into the study at which point each participant received a 45-minute counselling session from a dietician to help achieve weight loss of 1 pound/week. During intervention there were 6x30minute visits for dietary counselling. Participants in both groups received a booklet with the fat or carbohydrate content of common foods and instructions for self-monitoring  IG: Low carbohydrate  CG: Low fat  **Education delivery**: Group and individual | **Outcomes measured at:**3,6 and 12 months  **Outcome measures:**  **Primary:** Change in weight from baseline and HbA1C.  **Secondary:** Blood pressure, lipids |
| In type 2 diabetes, randomisation to advice to follow a low-carbohydrate diet transiently improves glycaemic control compared with advice to follow a low-fat diet producing a similar weight loss  Guldbrand, H. et al 2012 Sweden[16] | **N= 61**: IG: 30, CG:31  **Age** (years) Mean (SD): IG: 61.2(9.5), CG: 62.7(11)  **BMI** (kg/m2) Mean (SD): IG: 31.6(5.0), CG: 33.8(5.7) | **Intervention length:** 2 years.  **Intervention aim:** To compare the effects of dietary advice of a low carbohydrate diet (LCD) versus a low-fat diet (LFD).  **IG:** Low carbohydrate diet advice  **CG:** Low Fat diet  **Education delivery**: Group and individual | **Outcome measured at:** 6, 12 and 24 months  **Outcome measures**:  **Primary**: Weight, BMI and HbA1c  **Secondary:** HDL, LDL, cholesterol, systolic and diastolic BP |
| Effects of a Low-intensity Intervention That  Prescribed a Low-carbohydrate vs. a Low-fat  Diet in Obese, Diabetic Participants Iqbal, N.et al 2009 USA[17] | **N = 144 :** IG: 70 CG: 74  **Age** (years) Mean (SD): IG: 60.0 (8.9), CG: 60.0 (9.5) **BMI**(kg/m2) Mean (SD): IG: 38.1 (5.5), CG: 36.9 (5.3)  **Female** **sex** (%): IG: 15.7%, CG: 5.4 %  **Ethnicity**: IG: White (34.4%), African American (62.9%), Latino (1.4%), Other (1.4%) CG: White (51.4%), African American (44.6%), Latino (2.7%), Other (1.4%) | **Intervention length:** 24 months  **Intervention aim:** to determine whether outcomes achieved during short term intensive interventions could be achieved over a longer time, using a low intensity intervention.  All: Weekly x2hour group nutrition education sessions for first month followed by monthly sessions till end of intervention with dietician. 30minx5days/week moderate PA recommended with pedometers provided. Provision of educational guide to assist with dietary adherence.  **IG**: Low carbohydrate diet- 30g/day, no restrictions on fat or calorie intake.  **CG:** Low fat diet: ≤30% calories form fat with a calorie deficit of 500kcal/day.  **Education delivery:** Group | **Outcomes measured at:** 6,12 and 24 months  **Outcome measures:**  **Primary**: Weight loss  **Secondary:** HbA1c, serum lipids, dietary intake |
| Salba-chia (Salvia hispanica L.) in the treatment of overweight and obese patients with type 2 diabetes: a double-blind randomized controlled trial  Vuksan, V.et al 2017 Canada[18] | **N= 77**: IG: 39, CG: 38  **Age** (years) means (SD): IG: 60 (2), CG: 60 (2)  **Body weight** (kg) means (SD): IG: 84.1 (2.6), CG: 84.2 (2.7)  **BMI** (kg/m2) means (SD): IG: 31.0 (0.9), CG: 30.7 (0.7)  **Female** **sex** (%): IG:74%, CG: 65% | **Intervention length**: 6 months  **Intervention aim:** To determine whether incorporating saba chia seeds into diet will help with weight loss in comparison to an oat bran control when consumed with a calorie reduced diet.  Both IG and CG were on a calorie reduced diet for 6 months. All individuals were asked to consume 500kcal less per day.  **IG:** Saba-chia supplementation  **CG**: Oat-based bran supplementation  **Education delivery:**  Individual | **Outcomes measured at:** 6 months  **Outcome measures**:  **Primary:** Weight  **Secondary**: Waist circumference, body composition, glycaemic control, C-reactive protein, and obesity-related satiety hormones |
| **Weight or BMI reduction >5% at end of intervention** | | | |
| Targeting dietary fat or glycemic load in the treatment of obesity and type 2 diabetes: a randomized controlled trial  Fabricatore, A. N. et al 2011 USA[19] | **N = 79 :** IG: 40, CG: 39  **Age** (years) Mean(SD): IG: 52.8 (1.4), CG: 52.5 (1.3)  **BMI**(kg/m2) Mean (SD): IG: 36.7 (0.8), CG: 35.8 (0.7)  **Female** **sex** (%): IG: 80%, CG: 79.5 %  **Ethnicity**: IG: Caucasian (42.5%), African American (52.5%), Other (5%), CG: Caucasian (41.0%), African American (43.6%), Other (15.4%) | **Intervention length:** 40 weeks  **Intervention aim:** To compare a low glycaemic load diet with a low-fat diet for weight loss.  Both groups received an exercise prescription and group education sessions for 90minutes x weekly for 20 weeks followed by biweekly for 20 weeks. Calorie goal based on baseline weight. Gradual increase to 175minutes/week of moderate PA. Food diaries and recipe cards.  **IG**: Low glycaemic load prescription of ≤3 servings moderate glycaemic load and ≤ 1 serving high glycaemic load items/day.  **CG:** Low fat diet. Goal of ≤30% fat. Food diary. Low fat eating plan.  **Education delivery:** Group | **Outcomes measured at:** 20 and 40 weeks  **Outcome measures:**  **Primary**: Changes in weight and HbA1c  **Secondary:** Blood pressure, waist circumference, lipids |
| Weight loss in obese patients with type 2 diabetes: Effects of telemonitoring plus a diet combination  The Active Body control(ABC)Program  Luley, C.et al 2011 Germany[20] | **N= 70**: IG:35, CG: 35  **Age** (years) Mean (SD): IG: 57 (9), CG:58 (7)  **BMI** (kg/m2) Mean (SD): IG: 35.3 (5.7), CG:34.8 (5.9) kg/m2  **Female** **sex** (%): IG: 57%, CG: 46% | **Intervention length:** 6 months  **Intervention aim:** To evaluate the effectiveness of the ‘Active Body Control (ABC) Program’ for weight loss consisting of telemonitoring (weighing scales, accelerometer and a homebox which receives the data from the devices via Bluetooth and sends this by telephone link to a central server) of PA alongside a low- calorie diet  **IG:** The ABC program.  **CG:** Low fat diet and standard care.  **Education delivery:**  Group and individual | **Outcomes measured at:** 6 months  **Outcome measures:**  **Primary**: Change in BMI and weight from baseline  **Secondary:** Metabolic and CV risk markers and antidiabetic drug usage |
| Effects of an energy-restricted low-carbohydrate, high unsaturated fat/low saturated fat diet versus a high-carbohydrate, low-fat diet in type 2 diabetes: a 2-year randomized clinical trial Tay, J. et al 2018 Australia[21] | **N = 115:** IG:57, CG: 58  **Age** (years) Mean (95% CI): IG: 58 (56 to 60), CG: 58 (56 to 60)  **BMI** (kg/m2) Mean (95% CI): IG: 34.2 (33.1 to 35.3), CG: 35.1 (34.0 to 36.2)  **Female** **sex** (%): IG:36%, CG: 49 % | **Intervention length:** 2 years  **Intervention aim:** To determine whether a low carbohydrate high unsaturated fat/low saturated fat diet is better than a high carbohydrate low fat diet for glycaemic control and CV risk factor reduction.  All: isocaloric diets. Moderate energy restriction of 500-1000kcal/day deficit. Participants met dietician fortnightly for 12 weeks, then monthly. Daily food diaries. Supervised 60minute exercise classes 3days/week.  **IG**: Low carbohydrate - 14% carbohydrate (< 50 g/day), 28% protein and 58% total fat (35% monounsaturated fat and 13% polyunsaturated fat).  **CG:** High carbohydrate - 53% carbohydrate (low glycaemic foods) 17% protein and <30% total fat (15% monounsaturated fat and 9% polyunsaturated fat).  **Education delivery:** Individual | **Outcomes measured at:** Weight measured monthly. other outcomes at 24, and 52 weeks  **Outcome measures:**  **Primary**: HbA1c  **Secondary:** Weight, body composition, waist circumference, blood pressure, lipids, renal function, Glycaemic variability, antiglycaemic Medication Effect Score (MES) |
| **Low fat** | | | |
| **Weight or BMI reduction <5% at end of intervention** | | | |
| The Diabetes Excess Weight Loss (DEWL) Trial: a randomised controlled trial of high-protein versus high-carbohydrate diets over 2 years in type 2 diabetes  Krebs, J. D. et al 2012 New-Zealand[22] | **N = 419 :** IG: 207 CG: 212  **Age** (years) Mean (SD): IG: 57.7 (9.9), CG: 58.0 (9.2)  **BMI**(kg/m2) Mean (SD): IG: 36.6 (6.7), CG: 36.7 (6.4)  **Female** **sex** (%): IG1: 54%, CG: 66%  **Ethnicity**: IG: European (81%), Maori (22%), Pacific (8%), other (7%), **CG**: European (88%), Maori (9%), Pacific (3%), Other (3%) | **Intervention length:** 2 years  **Intervention aim:** To compare a low-fat high protein against a low-fat high carbohydrate diet on weight loss in overweight T2DM.  **IG**: low fat high protein (40% carbohydrate, 30% protein, 30% fat). Dietician led education sessions, 1 hour every 2 weeks for 6 months then monthly for 6 months which included goal setting. Weekly text/email to improve adherence. Individual dietary prescription based on energy requirement estimation. Sample diet plans and portion charts provided. Culturally appropriate recipes available.  **CG:** low fat high carbohydrate (55% carbohydrate, 15% protein, 30% fat). Individual dietary prescription based on energy requirement estimation. Sample diet plans and portion charts provided. Culturally appropriate recipes available.  **Education delivery: individual and** Group | **Outcomes measured at:** 6, 12 and 24 months  **Outcome measures:**  **Primary**: Change in weight and  waist circumference  **Secondary:** Body fatness, glycaemic control,  lipid profile, blood pressure, renal function, dietary adherence, Qol (SF36) |
| A portion-control plate was effective for weight loss in obese patients with type 2 diabetes mellitus  Pedersen, S.D. et al 2007 Canada [23] | **N = 130:** IG: 65, CG: 65  **Age** (years) Mean (SD): IG: 56.8 (9.2), CG: 55.3 (10.8)  **BMI** (kg/m2) Mean (SD): IG: 39.1 (6.5), CG: 38.8 (6.4)  F**emale** **sex** (%): IG: 67.7%, CG: 50.8% | **Intervention length:** 6 months  **Intervention aim:** To determine the effectiveness of a portion control tool aiding to control calorie intake for weight loss.  All participants had to have at least 6 months pre-study teaching and management by diabetes nurse educators and dieticians.  **IG**: Given a commercially available calibrated dinner plate (sex specific) and cereal bowel for portion control. Food diary. Individual counselling session with information booklet on how to use tools.  **CG:** Usual Care – dietary teaching.  **Education delivery:** Individual | **Outcomes measured at:** 6 months.  **Outcome measures:**  **Primary:** Weight change  **Secondary:** Blood pressure, HbA1C, lipids |
| **Weight or BMI reduction >5% at end of intervention** | | | |
| The maintenance of improved metabolic control after intensified diet therapy in recent type 2 diabetes  Uusitupaa M, et al 1993  Finland[24] | **N = 86:** IG: 40, CG: 46  **Age** (years): 40-64  **BMI** (kg/m2) Mean (SD): IG: 33.2 (5.5), CG: 32.7 (4.8)  **Female sex** (%): IG: 48%, CG: 39% | **Intervention length:** 12 months following 3 months basic education  **Intervention aim:** To determine if intensive dietary education results in better metabolic control and improvements in CV risk factors compared to usual care.  All participants received 3 months basic education  **IG:** Intensive diet education  **CG:** Usual care  **Education delivery:** Group and individual | **Outcomes measured at:** 6 weeks, 3, 9, 15 and 27 months  **Outcome measures: Primary not specified:** BMI, BP, HbA1c, lipids |
| **Low calorie, counselling and PA** | | | |
| **Weight or BMI reduction <5% at end of intervention** | | | |
| Living Well with Diabetes: a randomized controlled trial of a telephone-delivered  intervention for maintenance of weight loss, physical activity and glycaemic control in adults with type 2 diabetes  Eakin, E.G. et al 2014  Australia[25] | **N= 302 :** IG :151, CG : 151  **Age** (years) mean (SD): IG: 57.7 (8.1), CG: 58.3 (9.0)  **BMI** (kg/m2) Mean (SD): IG: 33.1 (6.3), CG: 33.2 (6.0)  **Weight** (Kg) Mean (SD): IG: 94.5 (18.7), CG: 95.3 (20.1)  **Female** **sex** (%)**:** All: 44% | **Intervention length:** 18 months  **Aims:** Assessing the effectiveness of a behavioural weight loss and PA intervention delivered by telephone in a primary care setting.  **IG:** Motivational interviewing targeting PA, reduced calorie intake (by approx. 500 kcal) and behaviour change. Participants received workbook and phone calls covering initiation and maintenance of weight loss periods. Phone calls helped identify benefits of weight loss, goal setting, self-monitoring, problem solving, using support available and focusing on targets achieved.  **CG:** Usual care.  **Education delivery:** Individual | **Outcomes measured at:** 6,18 and 24 months  **Outcomes measured:**  **Primary:** Difference between groups in weight change, physical activity accelerometer- measured moderate-to-vigorous physical activity, and glycaemic control (HbA1c)  **Secondary**: Change in self-reported dietary and energy intake, accelerometer-measured light intensity and sedentary  time, waist circumference, percent body fat, fasting plasma glucose and blood lipids, liver function, BP, medication use and  health-related quality of life |
| Effects of Aerobic Exercise Based upon Heart Rate at Aerobic Threshold in Obese Elderly Subjects with Type 2 Diabetes  Emerenziani, G. P. et al 2015  Italy[26] | **N=30**: IG: 15, CG:15  **Age** (years) mean (SD): IG: 66.7 (4.9) CG:66.9 (4.2)  **BMI** (kg/m2) Mean (SD) All: 34.6 (3.2) | **Intervention length:** 3 months  **Intervention aim:** To assess effects of a 3 month period of aerobic exercise, personalised to heart rate at AerTge (aerobic gas exchange threshold) and its effects on glycaemic control, body weight, and fitness.  **IG** Aerobic exercise group  **CG:** Usual care  **Education delivery**: Individual | **Outcomes measured at:** 3 months.  **Outcome measures:**  **Primary**: Glycaemic control, weight, BMI and fitness (peak oxygen uptake (VO2peak). The individual aerobic gas exchange threshold (AerTge)  **Secondary:** Abdominal circumference (cm), total cholesterol, high density lipoprotein cholesterol (HDLC), and low-density lipoprotein cholesterol (LDLC) |
| Pounds Off With Empowerment (POWER): A Clinical Trial of  Weight Management Strategies for Black and White Adults With  Diabetes Who Live in Medically Underserved Rural Communities  Mayer-Davis, E. J. et al 2004 USA[27] | **N = 152**: IG1: 49, IG2: 47, CG: 56  **Age** (years) mean (SD): IG1: 59.7 (8.6), IG2: 58.9 (7.8) CG: 62.4 (9.5)  **BMI** (kg/m2) Mean (SD): IG1: 37.6 (6.5), IG2: 37.5 (6.7) CG: 35.2 (7.5)  **Female** **sex** (%): IG1: 78%, IG2: 85%, CG: 79%  **Ethnicity**: African-American, IG1: 83.7%, IG2: 89.4%, CG: 73.2% | **Intervention length**: 12 months  **Intervention aim:** Achieving and maintaining 10% weight loss over a period of 12 months.  All participants had to complete a 3 day ‘run in’ period to show commitment to participation and the ability to self-monitor diet and PA.  **IG1:** Intensive lifestyle: derived from the lifestyle intervention of the diabetes prevention program (DPP). Modifications to the DPP included use of group as well as individual sessions, PA at low to moderate intensity for those who were previously sedentary with identification of PA e.g. places for walks, and regionally appropriate foods. intervention retained a clear focus on diet and physical activity.  **IG2:** The reimbursable-lifestyle: a shortened version of the intensive-lifestyle intervention  **CG:** Usual care  **Education delivery:** Individual and group | **Outcomes measured at:** 6 and 12 (weight/BMI only) months  **Outcome measures:**  **primary outcome:** Change in BMI and weight from baseline  **Secondary outcomes:** HbA1c, lipid profile, and blood pressure |
| **Modified fasting** | | | |
| **Weight or BMI reduction <5% at end of intervention** | | | |
| Effects of A One-week Fasting Therapy in Patients with Type-2 Diabetes Mellitus and Metabolic Syndrome – A Randomized Controlled  Explorative Study  Li, C. et al 2017 Germany[28] | **N = 46**: IG: 23, CG: 23  **Age** (years) Mean (SD): IG: 64.7(7.0), CG: 65.4 (5.7) ± 5.7  **BMI** (kg/m2) Mean (SD): IG: 30.7 (2.9), CG: 34.0 (6.7) | **Intervention length:** 4 months  **Intervention aim:** To determine the effects of a one-week fasting programme on metabolic and clinical factors.  **IG:** Fasting program and dietary advice  **CG**: Usual care and Mediterranean dietary advice.  **Education delivery:**  Group | **Outcomes measured at:** 16 +/- 2 weeks.  **Outcome measures:**  **Primary:** HbA1C  **Secondary:** Change in BMI and weight from baseline, serum glucose, lipids, blood pressure, quality of life and safety outcomes |
| **Weight or BMI reduction >5% at end of intervention** | | | |
| Weight loss technology for people with treated type 2 diabetes: a randomized controlled trial  Oshakbayev, K. et al 2017 Kazakhstan[29] | **N = 272 :** IG:208, CG: 64  **Age** (years) Mean(SD): IG: 47.7(1.8), CG: 46.5 (2.1)  **BMI**(kg/m2) Mean (SD): IG: 29.6 (0.4), CG: 29.3 (0.9)  **Female** **sex** (%): IG: 52%, CG: 53% | **Intervention length:** 24 weeks.  **Intervention aim:** To determine the effects of a weight loss method in individuals with T2DM on physiological parameters.  **IG**: Calorie restricted diet “Analimentary  detoxication” (ANADET) and PA. 100-150 kcal/day + fat free vegetables, salt limitation (5-6g/day), minimum of 8,000steps/day and sexual self-restraint. ANADET method lasted 14-28 days X2 periods, with 2 week break in-between. Followed by a diet of one meal a day of any foods.  **CG:** Conventional drug treatment with weight loss medication (orlistat 360 mg/day)  **Education delivery:** Individual | **Outcomes measured at:** 24 and 48 weeks  **Outcome measures:**  **Primary**: Weight loss,  fasting glucose and 2-hour oral glucose tolerance test (OGTT)  **Secondary:** Blood pressure,  lipid and insulin blood levels |
| **Meal replacements, low calorie** | | | |
| **low fat** | | | |
| **Weight or BMI reduction >5% at end of intervention** | | | |
| Effect of intensive dietetic interventions on weight and glycaemic control in overweight men with Type II diabetes: a randomised trial Ash, S. et al 2003 Australia[30] | **N = 51:** IG1: 14, IG2: 20, CG: 17  **Age** (years) Mean (SD): IG1: 54.3 (9.4), IG2: 54.2 (7.4), CG: 54.9 ( 9.3)  **BMI**(kg/m2) Mean (SD): IG1: 31.2 (3.4), IG2: 31.1 (3.7), CG: 32.7 (2.4)  **Female** **sex** (%): ALL: 0% | **Intervention length:** 12 weeks  **Intervention aim:** To determine the effectiveness of an innovative intensive dietary method on weight loss and glycaemic control.  Before randomisation, participants underwent 2 weeks of counselling on adjusting to a 1400-1700 kcal/day diet. During intervention contact with dietician and physician every 2 weeks with phone contact with dietician in-between contacts on alternate weeks.  **IG1**: Intermittent energy restriction (IER) – Participants supplied liquid meal replacements (Modifasts, Novartis Ltd) of 1000kcal/day for 4 days /week and eating when hungry on remaining 3 days of the week sticking to calorie limit.  **IG2:** Pre-portioned meals (PPM) – Pre-packaged meals and snacks supplied. 1650kcal/day.  **CG:** Self-selected meals (SSM) - usual dietary intervention  **Education delivery:** Individual and group | **Outcomes measured at:** 3 and 18 months  **Outcome measures: Primary not specified:** Weight, body composition, waist circumference, glycaemic control (HbA1c) and blood lipids |
| Efficacy of Meal Replacements Versus a Standard Food-Based Diet for Weight Loss in Type 2 Diabetes  Cheskin, L. J. et al 2008 USA[31] | **N= 112**: IG: 54, CG: 58  **Age** (years) Mean (SD): IG 54.6 (7.0), CG 55.48 (7.2)  **BMI** (kg/m2) Mean (SD): IG: 35.3 (3.5), CG: 35.7 (3.8)  **Female** **sex** (%): All: 56%  **Ethnicity**: IG: 79.6% Caucasian, CG: 74% Caucasian | **Intervention length**: 34 weeks (12-month maintenance intervention)  **Intervention aim:** To compare the effectiveness of a portion-controlled meal replacement diet (PCD) to a standard diet (SD) based on American Diabetes Association (ADA) recommendations on achieving and maintaining weight loss.  **IG:** The PCD group consumed 50% to 60% of prescribed calories from meal replacements.  **CG:** The SD group consumed their prescribed calories from whole foods from choices on the ADA exchange lists.  **Education delivery:** Individual and group | **Outcomes measured at:** 34, 60 (not presented) and 86 weeks  **Outcomes:**  **Primary**: Weight and BMI  **Secondary:** Glucose, insulin, HBA1c, cholesterol, triglycerides, blood pressure, waist and hip measurements |
| Improving Diabetes Outcomes Through Lifestyle Change – A Randomized Controlled Trial  Delahanty, L. M. et al 2015  USA[32] | **N= 57:** IG: 28, CG: 29  **Age** (Years) Mean (SD): IG: 62 (9.6), CG: 61 (11.4)  **BMI** (kg/m2) Mean (SD): IG: 36.3 (12.4), CG: 33.8 (5.0)  **Female** **sex** (%): IG: 39, CG: 41  **Ethnicity**: All: 32% non-white | **Intervention length**-19 weeks  **Intervention aim:** Comparison of a diabetes group lifestyle intervention versus dietitian referral for medical nutrition therapy (RD) for weight loss in the usual care setting.  Participants were selected after a run-in phase  **IG:** Group lifestyle intervention (GLI). Sessions covered nutrition, PA and behavioural strategies to lose weight. Used look ahead study publicly available group session material, meal replacements week 3 onwards (shakes, bars and pre-packaged meals), food and exercise diaries with review and feedback by dieticians.  **CG**: Dietitian-referral medical nutrition therapy (RD) – current standard care.  **Education delivery**: Group | **Outcomes measured at: baseline,** 6 months (and 1 year for weight loss)  **Outcome measures:**  **Primary:** Change in weight from baseline  **Secondary**: Mean weight loss, Hba1c, bp, HbA1c and fasting lipid levels |
| A randomized comparison of a commercially available portion-controlled weight-loss intervention with a diabetes self-management education program  Foster, G. D. et al 2013 USA[33] | **N= 100**: IG:50, CG: 50  **Age** (Years) Mean (SD): All: 55.6 (10.6)  **BMI** (kg/m2) Mean (SD): All: 35.8 (5.3), IG :35.3 (4.6), CG: 36.2 (5.8)  **Weight** (kg) mean (SD): All: 102.9 (18.4)  **Ethnicity**: All: 60% African Americans, 37% Caucasians and 3% Asian Americans | **Intervention length**:3 months  **Intervention aim:** To determine the effectiveness of a commercially available, portion-controlled diet (PCD) on body weight and glycaemic control.  Participants of both groups were prescribed the same calorie (1250–1550 kcal per day) and PA (>=200 min per week) goals. They both had 9 group sessions. Participants self-monitored glucose twice a day. The readings were reviewed at each session by staff.  **IG**: PCD - Women’s meal plans provided approx. 1250 and men’s meal plans 1550 kcal per day. The pre-packaged meals typically consisted of (three entrees and one snack daily) and these could be supplemented from a list (e.g. fruits, vegetables, dairy items and lean protein). Approximately 55% of total energy was made up from the packaged foods and 45% from supplemental grocery items. Each group session covered progress since last meeting followed by a new topic related to weight loss.  **CG:** Diabetes self-management education (DSME).  **Education delivery**: Group | **Outcomes measured at:** 3(not reported in paper) and 6 months  **Outcome measures:**  **Primary:** Change in BMI and weight from baseline  **Secondary**: Change in HbA1c at 6 months |
| Primary care-led weight management for remission of type 2 diabetes (DiRECT): an open-label, cluster-randomised trial  Lean M.E.J. et al 2017 UK[34]  **Cluster-randomised trial** | **N= 49:** IG: 149, CG: 149 participants (26 practices)  **Age** (years) mean (SD): IG: 52.9 (7.6), CG: 55.9 (7.3)  **BMI** (kg/m2) mean (SD): IG: 35.1 (4.5), CG: 34.2 (4.3)  **Weight** (kg) mean (SD): IG: 101·0 (16.7), CG: 98·8 (16.1)  **Ethnicity:** IG: 146 (98%) Caucasian | **Intervention length:** 6-7 months (18-month maintenance)  **Aims:** Determining whether an intensive weight loss intervention delivered in primary care, aiming at achieving and maintaining a 15kg weight loss in individuals with a diagnosis of T2DM within the previous 6 years results in diabetes remission.  **IG:** weight management programme (Counterweight-Plus)  Total Diet Replacement (TDR) 0-12 (can extend to 20 weeks if weight loss target not achieved) weeks with aim to lose 15kg. A commercial liquid diet (soups and shakes) provided 825-853 kcal/day+ fluids 2.25L.  Food reintroduction stage: 12- 18 weeks, progressive reintroduction of food.  Maintenance phase: 19-104 weeks, full food-based diet. Individualised prescription supporting weight stabilisation and preventing regain. Option of one sachet of formula diet /day available. Monthly reviews.  If capable advice to increase PA up to 15,000 steps/day (step counters provided). If needed ‘rescue plans’ to reverse weight gain available.  **Control:** standard care according to NICE guidelines.  **Education delivery:** Individual | **Outcomes measured at:** 12,24 months  **Outcome measures:**  **Primary:** Weight and Change in weight from baseline, HBA1c  **Secondary:** Quality of life(EQ-5D) , PA, lipids, Bp, LFT’s, U&E’s, plasma glucose, programme acceptability |
| **The Look AHEAD Trial**  **The Look AHEAD Research Group 2014 USA[35]**  Cluster randomised trial | **N= 5145**: IG: 2,570, CG: 2,575  **Age** (years) mean (SD): IG:58.6 (6.8), CG:58.9 (6.9)  **BMI** (kg/m2) Mean (SD): IG: 35.9 (6.0), CG: 36.0 (5.8)  **Female** **sex** (%): IG: 59.3%, CG: 59.6%  **Ethnicity**: 63.13% Caucasian, 37% racial or ethnic minorities | **Intervention length:** 12 months (7-year maintenance intervention)  **Intervention aim:** To determine if there is a reduction in cardiovascular morbidity and mortality in individuals with type II DM by sustained weight loss achieved through an intensive lifestyle intervention consisting of PA, diet and behaviour change.  Aim is to achieve a 7% weight loss in intervention participants.  All Participants were required to take part in a behavioural run in period over 2 weeks, where they self-monitored diet and PA and were required to pass an exercise test. They also received diabetes education prior to randomisation.  **IG:** Intensive Lifestyle Intervention**:** multicomponent, encouraging loss of individuals to lose 10% body weight, consisting of modifications to diet, PA and behavioural changes. Diet – portion-controlled calorie restricted diet (calorie recommendation 30% fat; >15%  Protein) consisting of structured dietary plan of liquid/meal bar replacement products for 2 meals and one portion-controlled snack from weeks 3-20, and one meal replacement till end of intervention.  **Year 1-** counselling for diet and PA. diet – portion-controlled calorie restricted diet. For first year participants were asked to record food and calorie intake daily, feedback was provided by counsellors at each visit. PA – tailored advice based on baseline PA tests, goal of 175min/week of moderate intensity walking or home-based exercise.  **CG:** Diabetes support and education  **Education delivery:** Individual and group | **Outcomes measured at: baseline,** 1,4 and 8 years.  **Outcomes:**  **Primary:** CV morbidity and mortality.  **Secondary:** Change in weight from baseline, medication use, HbA1C level, systolic BP, lipids. Health-related quality of life was assessed with the SF-36 and the Beck Depression Inventory II (BDI-II) |
| A randomized trial of improved weight loss with a prepared meal plan in overweight and obese patients: impact on cardiovascular risk reduction Metz, J. A. et al 2000 USA[36] | **N = 119,**  IG:56, CG: 63  **Age** (years) Mean (SD): IG: 54.6 (9.0), CG: 54.0 (9.9)  **BMI** (kg/m2) Mean (SD): IG: 33.0 (4.4), CG: 34.5 (4.5)  **Female** **sex** (%): IG: 31%, CG: 38 %  **Ethnicity**: IG: White 84%, African American 7%, other 9%, CG: White 76%, African American 14%, Other 10% | **Intervention length:** 13 months  **Intervention aim:** To determine the effects of a pre-packaged meal plan on weight and cardiovascular risk factors.  All participants underwent a 4-week monitoring period before the start of the intervention with 3 site visits for data collection and food diary reviews. Both diets consisted of 22% fat, 58% carbohydrate and 20% protein.  **IG**: Pre-prepared meal plans – 1 breakfast, lunch and dinner with 1 serving of fruit, diary product and vegetable daily.  **CG:** Usual care diet – based on American Diabetes Associations recommendations.  **Education delivery:** Individual | **Outcomes measured at:** 10,12, 24, 26, 50 and 52 weeks  **Outcome measures:**  **Primary**: Weight change  **Secondary:** Blood pressure, Lipids, HbA1C, QOL, nutrient intake and dietary compliance |
| **low carbohydrate** | | | |
| **Weight or BMI reduction >5% at end of intervention** | | | |
| Short-term safety, tolerability and efficacy of a very low-calorie-ketogenic diet interventional weight loss program versus hypocaloric diet in patients with type 2 diabetes mellitus  Goday, A., et al 2016 Spain[37]  Cluster randomised trial | **N = 89:** IG: 45, CG: 44  **Age** (years) Mean (SD): IG: 54.89 (8.81), CG: 54.17 (7.97)  **BMI** (kg/m2) Mean (SD): IG: 33.25 (1.52), CG: 32.88 (1.60)  **Weight** (Kg) Mean (SD): IG: 91.47 (11.43), CG: 89.54 (11.37) | **Intervention length:** 4 months  **Aims:** Determining the tolerability and short-term safety of VLCK (50grams/daily of carbohydrates) in a weight loss program including lifestyle and behavioural support (Diaprokal Method). Secondary aims included comparing weight loss and changes in metabolic parameters in participants taking part in intervention compared to low-fat low-calorie diet in conjunction with lifestyle and behaviour change.  **IG**: Commercial weight loss programme (DiaproKal Method) following a very low-calorie diet consisting of high protein preparations (meal replacements) and natural foods. The very low-calorie phase was maintained for variable lengths (typically 4-6 weeks) of time until each individual loses at least 90% of the weight loss target.  **CG:** Low calorie diet (500 – 1000kcal) based on each participant’s basal metabolic rate and recommendations from American Diabetes Association. Diet consisting of approx. 30% fat, 10-20% protein and 45-60% carbohydrates.  **Education delivery:** Group and individual | **Outcomes measured at:** 4 months  **Outcome measures:**  **Primary:** Safety and tolerability assessment: renal function, liver function, plasma uric acid, sodium and potassium  **Secondary:** Weight, BMI, waist circumference, HbA1c, lipids |
| Efficacy of the Telemedical  Lifestyle intervention Program  TeLiPro in Advanced Stages of  Type 2 Diabetes: A Randomized  Controlled Trial  Kempf (2) et al Germany  2017[38] | **N = 202:** IG:102, CG: 100  **Age** (years) Mean (SD): IG:59 (9), CG: 60 (8)  **BMI** (kg/m2) Mean (SD): IG: 35.3(5.9), CG: 37 (6.7)  **Female** **sex** (%): IG:45%, CG:47% | **Intervention length:** 12 weeks  **Intervention aim:** To determine the effectiveness of the telemedical lifestyle intervention programme for reductions in blood glucose, body weight and cardiovascular risk factors in individuals with established T2DM.  **IG**: The TeLiPro group received weighing scales, a step counter, meal replacements, glucose monitors in addition to usual care. The data was automatically sent to an online portal where participants could view the data. Received weekly calls providing information on T2DM, anti-diabetic medication, PA and diet.  **CG:** received routine care in addition to weighing scales and a step counter.  **Education delivery:** individual | **Outcomes measured at:** 12, 26 and 52 weeks  **Outcome measures:**  **Primary**: Change from baseline in HbA1c  **Secondary:** Weight change, antidiabetic medication, CVD risk factors, QOL |
| Weight Loss, Glycemic Control, and Cardiovascular Disease Risk Factors in Response to Differential Diet Composition in a Weight Loss  Program in Type 2 Diabetes:  A Randomized Controlled Trial  Rock, C. et al 2014 USA[39] | **N= 227:** IG1: 74, IG2: 77, CG: 76  **Age** (years) Mean: All: 56  **BMI** (kg/m2): n (%):  25-29.99: IG1: 4 (5.4), IG2: 6 (7.8), CG: 6 (7.9)  30-34.99: IG1: 28 (37.8), IG2: 27 (35.1), CG: 26 (34.2)  35-39.99: IG1: 24 (32.4), IG2: 26 (33.8) CG: 26 (34.2)  40-45: IG1: 18 (24.3), IG2: 18 (23.4), CG: 18 (23.7)  **Weight** (kg) n (%): All:105.5 (17.6)  **Ethnicity n** (%)  Non-Hispanic white: IG1: 59 (79.7), IG2: 63 (81.8),CG:59 (77.6)  Hispanic: IG1: 4 (5.4), IG2: 6 (7.8), CG: 7 (9.2)  African American: IG1: 7 (9.5) IG2: 2 (2.6), CG: 4 (5.3)  Asian: IG1: 4 (5.4), IG2: 4 (5.1), CG:2 (2.6)  Other: IG1: 0 (0), IG2: 2 (2.6), CG: 5 (5.3) | **Intervention length:** 12 months  **Intervention aim:** To determine whether participation in a commercial weight loss programme promotes greater 1-year weight losses, glycaemic control, and improved CV risk factors and maintenance.  **IGs:** Two intervention arms consisted of a commercial weight loss program consisting of face to face individual weight loss counselling focusing on behaviour change with telephone and website or message board follow up, low energy density diet pre-packed meals (3 entrees and 2 snacks for 7 days/week over months 1-6 and 5days/week during 7-9months with 1 entrée and 1 snack daily months 10-12) and increases to PA  **IG1:** Low fat diet  **IG2:** Low carbohydrate diet  **UC:** Usual care program  **Education delivery:** individual | **Outcomes measured at:** 6 and 12 months  **Outcome measures:**  **Primary:** Weight, BMI, change in weight and BMI from baseline  **Secondary:** Waist circumference, blood pressure, SF-36, fasting 6 hour Glucose, cholesterol, triglyceride, HDL cholesterol, and HbA1c |
| **Education and physical activity** | | | |
| **Weight or BMI reduction <5% at end of intervention** | | | |
| Effects of Nordic walking on cardiovascular risk factors in overweight individuals with type 2 diabetes, impaired or normal glucose tolerance  Fritz, T. et al 2013 Sweden[40] | **N = 50:** IG: 20, CG:30  **Age** (years) mean (SD): IG: 61.4 (-4.6), CG: 61.0 (-4.7)  **BMI** (kg/m2) Mean (SD): IG: 31.7 (-5.2), CG: 31.1 (3.9) | **Intervention length**:4 months  **Intervention aim:** To assess the effects of Nordic walking on CV risk factors.  **IG:** Nordic Walking  **CG:** Usual care  **Education delivery:** Individual | **Outcomes measured at:** 4 months  **Outcome measures: Primary not specified.** Change in BMI and weight from baseline, waist circumference, blood pressure, glucose tolerance, clinical chemistry, maximal oxygen uptake (peak VO2) and self-reported physical activity – estimate whether the physical activity was of low, medium or high intensity (not a validated questionnaire) |
| Autonomous exercise game use improves metabolic control and quality of life in type 2 diabetes patients - a randomized controlled trial  Kempf, K. et al 2013  Germany[41] | **N = 220**: IG: 120, CG:100  **Age** (years) Mean (SD): IG: 62 (11), CG: 60 (9)  **BMI** (kg/m2) Mean (SD): IG: 34.1 (6.5), CG: 33.2 (6.3) | **Intervention length:** 3 months  **Intervention aim:** To test the hypothesis autonomous use of the Wii Fit plus interactive exercise game over a period of 12 weeks results in improved metabolic control.  **IG:** Wii fit plus use  **CG:** Routine care  **Education delivery:** Individual | **Outcomes measured at:** 3 months  **Outcome measures:**  **Primary**: HbA1c reduction.  **Secondary:** Change in BMI and weight from baseline, reduction of cardiometabolic risk factors, physical activity and quality of life. |
| Does home-based exercise improve body mass index in patients with type 2 diabetes? Results of a feasibility trial  Krousel-Wood, M. A. et al 2008 US[42] | **N= 94**: IG: 45, CG: 49  **Age** (years) Mean (SD):56.6 (9.6)  **BMI** (kg/m2) Mean (SD): IG: 38.2 (8.2), CG: 37.0 (7.1)  **Female** **sex** (%): All:68%  **Ethnicity**: All: 47% black | **Intervention length**: 3 months  **Intervention aim:** Evaluation of the use, safety, and short-term benefits of a homebased exercise programme to increase PA.  All participants were given diabetes self-management education.  **IG:** Home based exercise video intervention  **CG:** Usual care  **Education delivery:** Individual and group | **Outcome measured at:** 3 months  **Outcome measures: Primary not specified:** BMI, quality of life, HbA1C, and blood pressure |
| The relationship between changes in steps/day and health outcomes after a pedometer-based physical  activity intervention with telephone support in type 2 diabetes patients Belgium  Dyck, D. et al 2013[43] | **N=92:** IG:60, CG:32  **Age** (years) Mean (SD): All: 62 (9)  **BMI** Mean (SD): All 30.0 (2.5) kg/m2, IG: 30.24 (2.62), CG: 29.74 (2.95)  **Weight** (kg) Mean (SD): IG: 89.22 (12.63), CG: 84.50 (12.38)  **Female** **sex** (%) All: 31% | **Intervention length:** 24 weeks  **Intervention aim:** To investigate health effects of a pedometer based behavioural modification intervention, assessing changes in steps/day related to health outcomes.  **IG**: Pedometer-based behavioural intervention. Participants were provided with a pedometer and diary to record steps, motivational interviewing, an individualized plan on how and where the behaviour changes would take place.  **CG:** Usual care  **Education delivery:** Individual | **Outcomes measured at:** 24-weeks and 1 year  **Outcome measures:**  **Primary:** PA and sedentary behaviour  **Secondary:** Systolic blood pressure, waist circumference, BMI, weight, glucose control (HbA1c and fasting glucose), triglycerides, total, HDL and LDL cholesterol |
| **Motivational interviewing** | | | |
| **Weight or BMI reduction <5% at end of intervention** | | | |
| Motivational Interviewing improves weight loss in women with type 2 DM  West, D.S. et al, 2007 USA[44] | **N = 217**: IG: 109, CG:108  **Age** (years) means (SD): All: 53(10), IG: 54 (10), CG: 52+/-10  **BMI** (kg/m2) mean (SD): All: 36.5 (5.5), IG 36.5 (5.5), CG: 36.5 (5.4)  **Weight** (kg) Means (SD): All: 97 (16), IG: 97 (17), CG: 97 (15)  **Ethnicity**: All: 38% African American  **Female** **sex** (%)**:** All: 100% | **Intervention length:** 6 months (12 months maintenance intervention)  **Intervention aim:** To identify if the addition of motivational interviewing to a behavioural weight control programme results in improved weight loss and glycaemic control.  **IG:** Motivational interviewing  **CG:** Individual health education sessions. Topics focussed on women’s health (e.g., breast self-care, skin care, sleep habits, etc.).  **Education delivery:**  Group and individual | **Outcomes measured at:** 6, 12 and 18 months  **Outcome measures:**  **Primary:** Weight, BMI, and HbA1C  No secondary outcomes specified |
| **Mindfulness** | | | |
| **Weight or BMI reduction <5% at end of intervention** | | | |
| Comparative Effectiveness of a Mindful Eating Intervention to a Diabetes Self-Management Intervention among Adults with Type 2 Diabetes: A Pilot Study  Miller, C. K. et al 2012 USA[45] | **N= 52:** IG: 27, CG: 25  **Age** (years) mean (SD): IG:53.9 (8.2), CG:54.0 (7.0)  **BMI** of 27 or more for inclusion  **Female** **sex** (%): IG: 63%, CG: 64%  **Ethnicity**: IG:81.5% white, CG:72.0% white | **Intervention length:** 3 months (3-month maintenance intervention)  **Intervention aim:** Mindful eating as an approach for weight and glycaemic control in comparison to Diabetes self-management education (DSME).  **IG:** Mindfulness-Based Eating Awareness Training (MB-EAT)  **CG:** The Smart Choices (SC) group based DSME.  **Education delivery:** Group | **Outcomes measured at:** 1,3, 6 months  **Outcome measures: Primary not specified:** Dietary intake, physical activity, Change in BMI and weight from baseline, HbA1c and fasting plasma glucose, and fasting insulin |
| Effects of a Multicomponent Life-Style Intervention on Weight, Glycemic Control, Depressive Symptoms, and Renal Function in Low-Income, Minority Patients With Type 2 Diabetes: results of the Community Approach to Lifestyle Modification for Diabetes Randomized Controlled Trial, Moncrieft, A. E. et al USA 2016[46] | **N = 111:** IG:57, CG: 54  **Age** (years) Mean (SD): IG: 54.84 (8.27), CG: 54.78 (6.34)  **BMI**(kg/m2) Mean (SD): IG: 32.3 (3.7), CG: 32.9 (5.5)  **Female** **sex** (%): IG: 64.9%, CG: 77.8 %  **Ethnicity**: IG: Hispanic (91.2%), Black (8.8%), White (0%), **CG**: Hispanic (77.78%), Black (13%), White (9.1%) | **Intervention length:** 1 year  **Intervention aim:** To review the effect of a multicomponent behavioural intervention in individuals from low income with T2DM who are overweight or obese with depressive symptoms.  **IG**: Community Approach to Lifestyle Modification for Diabetes (CALM-D). Lifestyle intervention based on the Diabetes Prevention Program. 17 sessions. Initial two were individual followed by two weekly, four biweekly and then monthly group sessions. 7% weight loss goal. 150minutes/week of aerobic exercise. Calorie goal based on body weight. Monitoring equipment including scales, food diary and activity monitor. Behavioural strategies to address depressive symptoms e.g. problem solving, addressing negative thoughts. Monetary incentive for completing assessment at baseline, 6 and 12 months.  **CG:** Usual Care – participants received educational booklet covering diabetes management related topics.  **Education delivery:** Individual and group | **Outcomes measured at:** 6 and 1 2 months  **Outcome measures:**  **Primary**: Weight, glycaemic control, and depressive  symptoms  **Secondary:** Renal function |
| **Coaching** | | | |
| **Weight or BMI reduction <5% at end of intervention** | | | |
| Peer Coaches to Improve Diabetes Outcomes in Rural  Alabama: A Cluster Randomized Trial  Safford, M. M.et al 2015 USA[47]  Primary care Practice cluster RCT | **N= 424**: IG: 198, CG: 226  **Age** (years) mean: All: 60.2  **BMI** (kg/m2) Mean (SD): All: 36.3 (8.5) , IG: 36.5 (7.7) , CG: 36.0 (9.1)  **Female** **sex** (%): All: 75.3%  **Ethnicity**: All: 87.4% African American | **Intervention length:** 10 months  **Intervention aim:** To assess the effects of an innovative peer coaching scheme and brief education for minority populations with DM residing in hard to reach areas (rural south) on diabetes health related outcomes.  **IG:** Peer coaches  CG: Usual care  **Education delivery:** Individual and group | **Outcomes measured at:** 6 and 12-15months  **Outcome measures:**  **Primary:** Change in HbA1c, systolic BP, LDL, Change in BMI from baseline, and quality of life.  **Secondary**: Diabetes distress scale and patient activation measure. |
| Remote Lifestyle Coaching Plus a Connected Glucose  Meter with Certified Diabetes Educator Support Improves  Glucose and Weight Loss for People with Type 2 Diabetes  Bollyky et al 2018 USA[48] | **N = 221:** IG1:64, IG2:67, CG:90  **Age** (years) Mean(SD): IG1: 49.9 (9.74), IG2: 51.3 (9.8), IG3: 49.8 (9.5), CG: 52.8 (11.2)  **BMI** not provided  Weight (lb) Mean (SD): IG1:244 (55), IG2: 246 (49), IG3: 224 (52) CG: not provided  **Female** **sex** (%): IG1:65.7%, IG2: 52.8%, IG3: 49.6% CG: 60%  **Ethnicity**: IG1: Caucasian (65.7%), Hispanic/Latino/Mexican: (1.5%), African American: (9%), unknown: (7.5%);  IG2: Caucasian (68.5%), Hispanic/Latino/Mexican: (0%), African American: (13.7%), unknown: (2.7%);  IG3: Caucasian (63.5%), Hispanic/Latino/Mexican: (1.7%), African American: (9.6) %, unknown: (15.7%);  CG Caucasian (60%), Hispanic/Latino/Mexican: (0%), African American:(12%), unknown: (18.7%) | **Intervention length:** 12 weeks  **Intervention aim:** To determine the intensity of coaching required in conjunction with monitoring health devices to improve weight and glycaemic outcomes.  All intervention group participants received a glucose monitor that stores data centrally with appropriate feedback messages, glucose strips. IG1 and IG2 had access to a certified diabetes educator to help with glucose readings, goal setting, individualized feedback and education. No calorie restriction.  **IG1**: ‘Connected weighing scale plus intense coaching’ + usual care: 60-minute initial call, personalized education and daily texts and PA recommendations.  **IG2:** ‘Connected weighing scale plus lightweight coaching’ + usual care: 20-minute initial call, standardised lessons and texts.  **CG**: Connected weighing scale + usual care: glucose monitor with messages, no access to coaches.  **Education delivery:** Individual | **Outcomes measured at:** 12 weeks  **Outcome measures:**  **Primary**: Change in weight from baseline, mean blood glucose, HbA1C  **Secondary:** Patient characteristics related to increased engagement with coaching programme |
| Optimizing diabetes control in people with Type 2  diabetes through nurse-led telecoaching  Odnoletkova, I. et al 2016  Belgium[49] | **N = 574:** IG: 287, CG: 287  **Age** (years) mean (S.D): IG: 63.8 (8.7), CG: 62.4 (8.9)  **BMI** (kg/m2) mean (SD): IG: 30 (5), CG: 31 (5)  **Female** **sex** (%): IG:40%, CG:37% | **Intervention length:** 6 months  **Intervention aim:** To assess the effects of a tele coaching intervention which is target driven on risk factors which are modifiable.  **IG:** “The COACH Program” is an already established intervention delivered by nurses or dieticians via telephone, designed to empower individuals to take responsibility to achieve and maintain targets related to diabetes risk factors e.g. related to lifestyle. The coaching helps to tackle knowledge and treatment gaps and motivate individuals.  CG: GP led usual care  **Education delivery:** Individual | **Outcome measured at:** 6 and 18 months  **Outcome measures:**  **Primary:** HbA1c  **Secondary:** HbA1c; total cholesterol, LDL, HDL, triglycerides, blood pressure, BMI, weight |

**Table S4. Risk of bias assessment for included trials**

| **Study** | **Domain**  **1** | **2** | **3** | **4** | **5** |  |
| --- | --- | --- | --- | --- | --- | --- |
|  | **Randomization process** | **deviations from the intended interventions** | **Missing outcome data** | **Measurement of the outcome** | **Selection of reported result** | **Overall** |
| **Adolfsson et al[1]** | **Some Concern** | **High Risk** | **High Risk** | **Low Risk** | **Some Concern** | **High Risk** |
| **Cheyette et al[2]** | **High Risk** | **High Risk** | **High Risk** | **Low Risk** | **Some Concern** | **High Risk** |
| **ICAN[3]** | **Low Risk** | **High Risk** | **Low Risk** | **Low Risk** | **Some Concern** | **High Risk** |
| **Samuel-Hodge et al[4]** | **Some Concern** | **High Risk** | **Low Risk** | **Low Risk** | **Some Concern** | **High Risk** |
| **Campbell et al[5]** | **Some Concern** | **High Risk** | **High Risk** | **Low Risk** | **High Risk** | **High Risk** |
| **D’Eramo-Melkus et al[6]** | **Some Concern** | **High Risk** | **Low Risk** | **High Risk** | **Some Concern** | **High Risk** |
| **Mitra et al[7]** | **Some Concern** | **Low Risk** | **Low Risk** | **Low Risk** | **Some Concern** | **Some Concern** |
| **Orsama et al[8]** | **Some Concern** | **High Risk** | **Low Risk** | **High Risk** | **Some Concern** | **High Risk** |
| **Prezio et al[9]** | **High Risk** | **High Risk** | **Low Risk** | **Low Risk** | **Some Concern** | **High Risk** |
| **Allen et al[10]** | **Low Risk** | **High Risk** | **Low Risk** | **Low Risk** | **Some Concern** | **High Risk** |
| **Christian et al[11]** | **Low Risk** | **High Risk** | **High Risk** | **Low Risk** | **Some Concern** | **High Risk** |
| **O’Neil et al[12]** | **Some Concern** | **High Risk** | **High Risk** | **Low Risk** | **Some Concern** | **High Risk** |
| **Brehm et al[13]** | **Some Concern** | **Low Risk** | **Low Risk** | **Low Risk** | **Some Concern** | **Some Concern** |
| **Daly et al[14]** | **High Risk** | **High Risk** | **High Risk** | **Low Risk** | **Some Concern** | **High Risk** |
| **Davis et al[15]** | **Some Concern** | **Low Risk** | **Low Risk** | **Low Risk** | **Some Concern** | **High Risk** |
| **Guldbrand et al[16]** | **Low Risk** | **Low Risk** | **Low Risk** | **Low Risk** | **Some Concern** | **High Risk** |
| **Iqbal et al[17]** | **High Risk** | **High Risk** | **High Risk** | **Low Risk** | **Some Concern** | **High Risk** |
| **Vuksan et al[18]** | **Some Concern** | **Low Risk** | **Low Risk** | **Low Risk** | **Some Concern** | **Some Concern** |
| **Fabricatore et al[19]** | **Some Concern** | **Low Risk** | **High Risk** | **Low Risk** | **Some Concern** | **High Risk** |
| **Luley et al[20]** | **Low Risk** | **High Risk** | **Low Risk** | **High Risk** | **Low Risk** | **High Risk** |
| **Tay et al[21]** | **Low Risk** | **Low Risk** | **High Risk** | **Low Risk** | **Low Risk** | **High Risk** |
| **Krebs et al[22]** | **Low Risk** | **High Risk** | **High Risk** | **Low Risk** | **Some Concern** | **High Risk** |
| **Pedersen et al[23]** | **Low Risk** | **High Risk** | **Low Risk** | **Low Risk** | **Some Concern** | **High Risk** |
| **Uusitupaa et al[24]** | **High Risk** | **High Risk** | **High Risk** | **Low Risk** | **Low Risk** | **High Risk** |
| **Eakin et al[25]** | **Some Concern** | **High Risk** | **High Risk** | **Low Risk** | **Low Risk** | **High Risk** |
| **Emerenziani et al[26]** | **High Risk** | **High Risk** | **Low Risk** | **Low Risk** | **Some Concern** | **High Risk** |
| **Mayer D[27]avis et al** | **Low Risk** | **High Risk** | **High Risk** | **Low Risk** | **Low Risk** | **High Risk** |
| **Li et al[28]** | **Low Risk** | **High Risk** | **Low Risk** | **Low Risk** | **Low Risk** | **High Risk** |
| **Oshakbavev et al[29]** | **Some Concern** | **Low Risk** | **High Risk** | **Low Risk** | **Some Concern** | **High Risk** |
| **Ash et al[30]** | **Some Concern** | **Low Risk** | **Low Risk** | **Low Risk** | **Some Concern** | **Some Concern** |
| **Cheskin et al[31]** | **Some Concern** | **High Risk** | **High Risk** | **Low Risk** | **Some Concern** | **High Risk** |
| **Delahanty et al[32]** | **Some Concern** | **High Risk** | **Low Risk** | **Low Risk** | **Some Concern** | **High Risk** |
| **Foster et al[33]** | **Some Concern** | **High Risk** | **Low Risk** | **Low Risk** | **Some Concern** | **High Risk** |
| **Lean et al[34]** | **Some Concern** | **High Risk** | **High Risk** | **Low Risk** | **Low Risk** | **High Risk** |
| **Look Ahead[35]** | **Low Risk** | **High Risk** | **Low Risk** | **Low Risk** | **Low Risk** | **High Risk** |
| **Metz et al[36]** | **High Risk** | **High Risk** | **High Risk** | **Low Risk** | **Some Concern** | **High Risk** |
| **Goday et al[37]** | **Some Concern** | **Low Risk** | **Low Risk** | **Low Risk** | **Some Concern** | **High Risk** |
| **Kempf 2 et al[38]** | **Low Risk** | **Some Concern** | **Low Risk** | **Low Risk** | **Some Concern** | **Some Concern** |
| **Rock et al[39]** | **Some Concern** | **High Risk** | **Low Risk** | **Low Risk** | **Some Concern** | **High Risk** |
| **Fritz et al[40]** | **Some Concern** | **High Risk** | **Low Risk** | **High Risk** | **Low Risk** | **High Risk** |
| **Kempf et al[41]** | **Low Risk** | **Low Risk** | **Low Risk** | **Low Risk** | **Some Concern** | **Some Concern** |
| **Krousel wood et al[42]** | **Some Concern** | **High Risk** | **High Risk** | **Low Risk** | **Some Concern** | **High Risk** |
| **Van Dyck et al[43]** | **Some Concern** | **High Risk** | **Low Risk** | **Low Risk** | **Low Risk** | **High Risk** |
| **West et al[44]** | **Some Concern** | **High Risk** | **Low Risk** | **Low Risk** | **Some Concern** | **High Risk** |
| **Miller et al[45]** | **Low Risk** | **Low Risk** | **Low Risk** | **Low Risk** | **Some Concern** | **Some Concern** |
| **Moncrieft et al[46]** | **Low Risk** | **High Risk** | **High Risk** | **Low Risk** | **Some Concern** | **High Risk** |
| **Safford et al[47]** | **Low Risk** | **High Risk** | **Low Risk** | **High Risk** | **Some Concern** | **High Risk** |
| **Bollyky et al[48]** | **High Risk** | **High Risk** | **Low Risk** | **Low Risk** | **Some Concern** | **High Risk** |
| **Odnoletkova et al[49]** | **Low Risk** | **High Risk** | **Low Risk** | **Low Risk** | **Some Concern** | **High Risk** |

| **Table S5: Intervention and control group weight and BMI outcomes of all included trials** | | | | | | | |
| --- | --- | --- | --- | --- | --- | --- | --- |
|  | | | |  | | | |
| **WEIGHT** | | | | **BMI** | | | |
| **Education alone** | | | | | | | |
| **Weight or BMI reduction <5% at end of intervention** | | | | | | | |
| **Adolfsson et al[1]** | **(IG) n=50** | **(CG) n=51** | **Between group difference** |  | **(IG) n=50** | **(CG) n=51** | **Between group difference** |
|  | **Mean (SD) Kg** | **Mean (SD) Kg** | **Kg [95% CI]** |  | **Mean (SD) Kg/m^2^** | **Mean (SD) Kg/m^2^** | **Kg/m^2^ [95% CI]** |
| Baseline | 86.9 (13.7) | 86.7 (12.6) |  | Baseline | 30.4 (4.3) | 29.6 (3.3) |  |
|  |  |  |  | 12 months | 30.0 (4.0) | 29.4 (3.3) | -0.1 [-0.6 to 0.4]  ^P value not reported^ |
| **Cheyette et al[2]** | **(IG) n=29** | **(CG) n=20** | **Between group difference** |  |  |  |  |
|  | **Mean (SD) Kg** | **Mean (SD) Kg** |  |  |  |  |  |
| Baseline | 96.7 (15.7) | 91.6 (14.8) |  |  |  |  |  |
| 4 months  (change from baseline) | 94.5 (15.8)  -2.2 (2.7)  P <0.01^b^ | 91.3 (14.8)  -0.3 (not reported)  P=0.37^b^ | Not reported |  |  |  |  |
| 6 months | 92.8 (13.2)  P=NS^b^ | 92.5 (15.6)  P=NS^b^ | Not reported |  |  |  |  |
| 12 months | 93.4 (14.2)  P=NS^b^ | 92.9 (16.1)  P=NS^b^ | Not reported |  |  |  |  |
| **ICAN study[3]** | **Intervention group (IG)**  **n=74** | **Control group (CG)**  **n=73** | **Between group difference** |  |  |  |  |
|  | **Mean (SD) Kg [95% CI]** | **Mean (SD) Kg**  **[95% CI]** | **Difference between means [95% CI]** |  |  |  |  |
| Baseline | 107.1 (25.5) | 106.7 (24.3) |  |  |  |  |  |
| 8 months (change from baseline) | – 4.0  [- 5.6 to - 2.5] | Not reported | - 5.0  [- 7.2 to - 2.9]  P<0.001^a^ |  |  |  |  |
| 12 months (change from baseline) | -2.4  [-4.1 to-0.6] | 0.6  [-1.0 to 2.2] | -3.0  [- 5.4 to - 0.6]  P=0.05^a^ |  |  |  |  |
| **Samuel-Hodge CD et al[4]** | **(IG) n=36** | **(CG) n=17** |  |  | **(IG) n=36** | **(CG) n=17** |  |
|  | **Mean (SE) Kg** | **Mean (SE) Kg** | Between Group Difference **Mean (SE) Kg** |  | **Mean (SE) Kg/m^2^** | **Mean (SE) Kg/m^2^** | Between Group Difference **Mean (SE) Kg/m^2^** |
| Baseline | 105.4 | 107.1 |  | Baseline | 38.1 | 37.1 |  |
| 20 weeks ( Adjusted Change from baseline) | -4.3 (0.74) | 1.4 (0.87) | -5.7 (1.1)  P<0.00001 | 20 weeks (Adjusted Change from baseline) | -1.6 (0.25) | 0.44 (0.30) | -2.0 (0.38)  P<0.0001 |
| *Adjusted for baseline value, diastolic blood pressure, and weekly frequency of eating breakfast.* | | | | | | | |
| **Weight or BMI reduction >5% at end of intervention** | | | | | | | |
|  |  |  | **BMI** | | | | |
| **Campbell et al[5]** |  |  |  | **IG1 n=57** | **IG2 n=66** | **IG3 n=59** | **CG n=59** |
|  |  |  |  | **Mean (SE) Kg/m^2^** | **Mean (SE) Kg/m^2^** | **Mean (SE) Kg/ m^2^** | **Mean (SE) Kg/m^2^** |
|  |  |  | Baseline | 31.4 (0.9) | 30.0 (0.6) | 29.7 (0.7) | 31.5 (0.9) |
|  |  |  | 3 Months | -1.9 (0.2) | -1.6 (0.2) | -2.1 (0.2) | -1.8 (0.3) |
|  |  |  | 6 Months | -2.2 (0.3) | -2.2 (0.3) | -2.5 (0.4) | -1.4 (0.4) |
|  |  |  | 12 Months | -2.0 (0.4)  P=NS^b^ | -1.4 (0.5)  P=NS^b^ | -2.6 (0.5)  P=NS^b^ | Not reported |
| **WEIGHT** |  |  |  | **BMI** |  |  |  |
| **D’Eramo-Melkus et al[6]** | **(IG) n=28** | **(IG2) n=26** | **(CG) n=28** |  |  |  |  |
|  | **Mean (SD) Lbs** | **Mean (SD) Lbs** | **Mean (SD) Lbs** |  |  |  |  |
| Baseline | 211.84 (27.78) | 200.65 (30.70) | 215.25 (25.47) |  |  |  |  |
| 3 Months | 199.96 (30.13)  P<0.01^b^ | 192.42 (32.09)  P<0.01^b^ | 209.46 (25.14)  P<0.01^b^ |  |  |  |  |
| 6 Months | 200.72 (30.44)  P=NS^b^ | 191.80 (31.73)  P=NS^b^ | 205.14 (25.59) |  |  |  |  |
| **Mitra et al[7]** |  |  |  |  | **(IG + CG) n=60** |  |  |
|  |  |  |  |  | **Mean (SD) Kg/m^2^** | **Mean (SD) Kg/m^2^** | Between Group Difference **Kg/m^2^** |
|  |  |  |  | Baseline | 27 (0.92) | Not reported |  |
|  |  |  |  | 12 months | 25 (0.42) | 26 (0.65) | P=”significant”^d^ |
| **Orsama et al[8]** | **(IG) n=24** | **(CG) n=24** |  |  | **(IG) n=24** | **(CG) n=24** |  |
|  | **Mean (CI) Kg** | **Mean (CI) Kg** | **Between Group Difference** |  | **Mean (SD) Kg/m^2^** | **Mean (SD) Kg/m^2^** |  |
| Baseline | Not reported | Not reported |  | Baseline | 30.7 | 33.5 |  |
| 10 months (change from baseline) | -2.1 [-3.6 to -0.6] | 0.4 [-1.1 to 1.9] | P=0.021^a^ |  |  |  |  |
| **Prezio et al[9]** |  |  |  |  | **(IG) n=90** | **(CG) n=90** |  |
|  |  |  |  | **Mean [95% CI] Kg/m^2^** | **Mean [95% CI] Kg/m^2^** | **Mean [95% CI] Kg/m^2^** |  |
|  |  |  |  | Baseline | 32.2 [30.5 to 33.9] | 34.4 [32.8 to 36.1] | Not reported |
|  |  |  |  | 12 months  (adjusted change from baseline) | 32.6 [31 to 31.3]  0.4 [-0.3 to 1.1]  *1.2%* | 35.0 [32.8 to 36.7]  0.6 [-0.1 to 1.3]  *1.7%* | -0.02 [-1.1 to 0.8]  P=0.78^a^ |
| *BMI: body mass index; adjusted for gender, HbA1c change over 12 months, total number of changes to diabetes medication over 12 months, smoking status and activity level at baseline. LDL: low density lipoprotein; HDL: high density lipoprotein; Trig: triglycerides.* | | | | | | | |
| **Education and Counselling** | | | | | | | |
| **Weight or BMI reduction <5% at end of intervention** | | | | | | | |
|  |  |  |  |  | **(IG) n=35** | **(CG) n=35** |  |
| **Allen et al[10]** |  |  |  |  | **Mean (SD) Kg/m^2^** | **Mean (SD) Kg/m^2^** | **Difference between means (SD) Kg/m^2^** |
|  |  |  |  | Baseline | 37.11 (6.68) | 33.8 (4.86) |  |
|  |  |  |  | 2 months | 36.58 (6.61)  P<0.05^b^  *-1.4%* | 33.93 (5.34)  P=ns  *0.03%* | -0.53 (0.75)  P<0.05^a^ |
| **Christian et al[11]** | **(IG) n=155** | **(CG) n=155** |  |  |  |  |  |
|  | **Mean (SD) Lbs**  **[95% CI]** | **Mean (SD) Lbs**  **[95% CI]** |  |  |  |  |  |
| Baseline | 207.0 (47.3) | 200.2 (44.7) |  |  |  |  |  |
| 12 months (change from baseline) | -0.18 (10.92)  [-2.00 to 1.65] | 1.39 (10.60)  [–0.43 to 3.22] | P=0.23^a^ |  |  |  |  |
| **O’Neil et al[12]** | **(IG) n=279** | **(CG) n=284** |  |  |  |  |  |
|  | **Mean (SD) Kg** | **Mean (SD) Kg** |  |  |  |  |  |
| Baseline | 104.0 (19.4) | 106.2 (19.9) | Not reported |  |  |  |  |
| 3 months | 99.9 (19.2) | 104.6 (19.7) | Not reported |  |  |  |  |
| 6 months | 99.7 (20.1) | 104.6 (19.7) | Not reported |  |  |  |  |
| 9 months | 99.8 (20.1) | 103.7 (19.9) | Not reported |  |  |  |  |
| 12 months | 99.6 (19.3) | 104.4 (20.1) | Not reported |  |  |  |  |
|  |  |  | P<0.001^c^ |  |  |  |  |
| **Education and low-calorie diet** | | | | | | | |
| **Low carbohydrate** | | | | | | | |
| **Weight or BMI reduction <5% at end of intervention** | | | | | | | |
| **Brehm et al[13]** | (IG) = 52 | (CG) = 43 |  |  |  |  |  |
|  | Mean (SE) Kg | Mean (SE) Kg |  |  |  |  |  |
| Baseline | 102.1 (2.0) | 103.7 (2.8) |  |  |  |  |  |
| 4 months | 98.2 (2.0) | 99.2 (2.8) |  |  |  |  |  |
| 8 months | 98.3 (2.1) | 99.3 (2.9) |  |  |  |  |  |
| 12 months | 98.3 (2.0) | 99.7 (3.0) |  |  |  |  |  |
|  | -4.0 (0.8) | -3.8 (0.6) | P=0.867 |  | -1.3 | -1.4 | P=0.720 |
| **Daly et al[14]** | **(IG) n=51** | **(CG) n=51** | **Between group difference** |  |  |  |  |
|  | **Mean (SE) Kg** | **Mean (SE) Kg** | **Difference between means [95% CI]** |  |  |  |  |
| Baseline | 101.6 (1.84) | 102.3 (2.49) |  |  |  |  |  |
| 3 months (change from baseline) | -3.55 (0.63) | -0.92 (0.40) | -2.63  [1.16 to 4.09]  P= 0.001^a^ |  |  |  |  |
| **Davis et al[15]** | **(IG) n=55** | **(CG) n=50** |  |  |  |  |  |
|  | **Mean (SD) Kg** | **Mean (SD) Kg** | Between group difference over time |  |  |  |  |
| Baseline | 93.6 (18) | 101 (19) | Not reported |  |  |  |  |
| 3 months | -5.2 (2.8) | -3.2 (3.7) | Not reported |  |  |  |  |
| 6 months | -4.8 (3.5) | -4.4 (5.3) | Not reported |  |  |  |  |
| 12 months | -3.1 (4.8)  -3.3% | -3.1 (5.8)  -3.1% | Not reported |  |  |  |  |
|  |  |  | P=0.005^c^ |  |  |  |  |
| **Guldbrand et al[16]** | **(IG) n=30** | **(CG) n=31** | **Between group difference** |  | **(IG) n=30** | **(CG) n=31** | **Between group difference** |
|  | **Mean (SD) Kg** | **Mean (SD) Kg** |  |  | **Mean (SD) Kg/m^2^** | **Mean (SD) Kg/m^2^** |  |
| Baseline | 98.8 (21) | 91.4 (19) |  | Baseline | 33.8 (5.7) | 31.6 (5.0) |  |
| 6 months | 94.2 (21)  P<0.001^b^ | 87.5 (19)  P<0.001^b^ | Not reported | 6 months | 32.3 (5.5)  P<0.001^b^ | 30.1 (5.1)  P<0.001^b^ | Not reported |
| 12 months | 94.9 (21)  P<0.001^b^ | 89.5 (19)  P<0.001^b^ | Not reported | 12 months | 32.6 (5.3)  P<0.001^b^ | 30.7 (5.3)  P<0.001^b^ | Not reported |
| 24 months | 95.9 (21)  P = 0.002^b^ | 89.4 (22)  P = 0.020^b^ | Not reported | 24 months | 32.8 (5.5)  P=0.002^b^ | 30.8 (5.8)  P=0.011^b^ | Not reported |
|  | P<0.001^c^ | P<0.001^c^ | P = 0.33^a^  ^(For change over all time points between groups)^ |  | P<0.001^c^ | P<0.001^c^  ^(For change over all time points between groups)^ | P=0.20^a^ |
| **Iqbal et al[17]** | (IG) = 70 | (CG) = 74 | Difference in Change over time |  | (IG) = 70 | (CG) = 74 |  |
|  | Mean (SD) Kg | Mean (SD) Kg |  |  | Mean (SD) Kg/m2 | Mean (SD) Kg/m2 |  |
| Baseline | 118.3 (21.3) | 115.5 (16.7) |  |  | 38.1 (5.5) | 36.9 (5.3) |  |
| 6 months (change from baseline) | -2.8 | -2.0 |  |  |  |  |  |
| 12 months (change from baseline) | -1.3 | -1.2 |  |  |  |  |  |
| 24 months (change from baseline) | -1.5 | -0.2 | P=0.15 |  |  |  |  |
|  |  |  |  |  |  |  |  |
| **Vuksan et al[18]** | **(IG) n=39** | **(CG) n=38** | **Between group difference** |  |  |  |  |
|  | **Mean (SD) Kg** | **Mean (SD) Kg** |  |  |  |  |  |
| Baseline | 84.1 (2.8) | 83.8 (2.6) |  |  |  |  |  |
| 6 months | 82.2 (0.5)  P<0.05^b^ | 83.5 (0.5)  ^P value not reported^ | P=0.02^a^ |  |  |  |  |
| **Weight or BMI reduction >5% at end of intervention** | | | | | | | |
| **Fabricatore et al[19]** | Mean kg (SE) | Mean kg (SE) |  |  | Mean (SE) Kg/m2 | Mean (SE) Kg/m2 |  |
| Baseline | 102.3 (2.7) | 99.1 (2.3) | P=0.37 |  | 36.7 (0.8) | 35.8 (0.7) | P=0.44 |
| 20 weeks (% Change from baseline) | -6.7 (0.7) | -5.7 (0.6) | P=0.26 |  |  |  |  |
| 40 weeks (% change from baseline) | -6.4 (1.3) | -4.5 (1.2) | P=0.28 |  |  |  |  |
| **Luley et al[20]** | **(IG) n=35** | **(CG) n=35** |  |  | **(IG) n=27** | **(CG) n=25** |  |
|  | **Mean (SD) Kg** | **Mean (SD) Kg** |  |  | **Mean (SD) Kg/m^2^** | **Mean (SD) Kg/m^2^** |  |
| Baseline | 102.1 (20) | 101.4 (17) |  | Baseline | 35.3 (5.7) | 34.8 (5.9) |  |
| 6 months (change from baseline) | -11.8 (7.6)  P=0.000^b^ | -0.3 (2.9)  P=ns | P=0.000^a^ | 6 months (change from baseline) | -4.1 (2.6)  P=0.000^b^ | -0.1 (1.0)  P=ns | P=0.000^a^ |
|  | (IG) = 58 | (IG2) = 57 | Mean difference in change between groups [95% CI] |  | (IG) = 58 | (IG2) = 57 | Mean difference in change between groups [95% CI] |
| **Tay et al[21]** | Mean kg [95% CI] | Mean kg [95% CI] |  |  | Mean kg/2 [95% CI] | Mean kg/m2 [95% CI] |  |
| Baseline | 101.7 [97.8 to 105.7] | 101.6 [97.6 to105.6] |  |  | 31.2 [33.1 to 35.3] | 35.1 [34.0 to 36.2] |  |
| 24 months (change from baseline) | -6.8 [-8.8 to -4.7] | -6.6 [-8.8 to -4.5] | -0.1 [-3.1 to 2.8]  P0.26 |  | -2.1 [-2.8 to -1.5] | -2.3 [-3.0 to -1.6] | 0.1 [-0.8 to 1.1]  P0.33 |
| **Low fat** | | | | | | | |
| **Weight or BMI reduction <5% at end of intervention** | | | | | | | |
| **Krebs et al[22]** | (IG) = 207 | (CG) = 212 |  |  | (IG) = 207 | (CG) = 212 |  |
|  | Mean (SD) Kg | Mean (SD) Kg | Difference in Change over time [95%CI] |  | Mean (SD) Kg/m2 | Mean (SD) Kg/m2 |  |
| Baseline | 103.4 (19.7) | 101.9 (20.1) |  | Baseline | 36.6 (6.7) | 36.7 (6.4) |  |
| 6 months | 100.2 (18.8) | 98.7 (19.3) |  |  |  |  |  |
| 12 months | 100.2 (17.8) | 99.5 (19.1) |  |  |  |  |  |
| 24 months** | 99.5 (17.2) | 95.9 (17.1) | 0.00 [-1.20, 1.21]  P0.73 |  |  |  |  |
| **Pedersen et al[23]** | (IG) = 65 | (CG) = 65 |  |  | (IG) = 65 | (CG) = 65 |  |
|  | Mean (SD) Kg | Mean (SD) Kg |  |  | Mean (SD) Kg/m2 | Mean (SD) Kg/m2 |  |
| Baseline | 105.2 (20.4) | 106.0 (16.8) |  | Baseline | 39.1 (6.5) | 38.8 (6.4) |  |
| 6 months (change from baseline) | -2.1 (4.9) | -0.1 (3.5) | P0.01 |  |  |  |  |
| 6 months (% change from baseline) | -1.8 (3.9) | -0.1 (3.0) | P0.006 |  |  |  |  |
| **Weight or BMI reduction >5% at end of intervention** | | | | | | | |
| **Uusitupaa et al[24]** |  |  |  |  | **(IG) n=40** | **(CG) n=46** | **Between group difference** |
|  |  |  |  | Baseline | 33.2 (5.5) | 32.7 (4.8) |  |
|  |  |  |  | 3 months | 32.0 (5.2) | 31.6 (4.8) | Not reported |
|  |  |  |  | 9 months | 31.8 (5.3) | 31.4 (5.0) | Not reported |
|  |  |  |  | 15 months | 31.4 (5.0)  P = 0.005  ^(3 versus 15 months)^ | 31.9 (4.6)  P value not reported | Not reported |
|  |  |  |  | 27 months** | 31.9 (5.0)  P = NS^b^ | 32.2 (4.5) | Not reported |
| **Education, low-calorie diet, counselling and PA** | | | | | | | |
| **WEIGHT** | | | | |  |  |  |
| **Weight or BMI reduction <5% at end of intervention** | | | | | | | |
| **Eakin et al[25]** | **(IG) n=151** | **(CG) n=151** |  |  |  |  |  |
|  | **Multiple imputation**  **Difference Between groups** | **Between group difference** | **Completers**  **Difference Between groups** | **Between group difference** |  |  |  |
|  | **Mean % reduction [95% CI]** |  | **Mean % reduction [95% CI]** |  |  |  |  |
| 6 months (change from baseline) | -1.31 [-2.40 to -0.22] | P=0.019^a^ | -1.29 [-2.13 to -0.46] | P=0.002^a^ |  |  |  |
| 18 months (change from baseline) | -1.42 [-2.54 to -0.30] | P=0.013^a^ | -1.37 [-2.56 to -0.18] | P=0.024^a^ |  |  |  |
| 24 months (change from baseline)** | -0.72 [-1.85 to 0.41] | P=0.212^a^ | -0.61 [-1.95 to 0.73] | P=0.371 |  |  |  |
|  | **Mean Kg [95% CI]** |  | **Mean Kg [95% CI]** |  |  |  |  |
| 6 months (Change from baseline) | -1.31 [-2.40 to -0.22)] | P=0.019 | -1.30 [-2.14 to -0.46] | P=0.003^a^ |  |  |  |
| 18 months (Change from baseline) | -1.52 [-2.64 to -0.39] | P=0.008 | -1.45 [-2.63 to -0.26] | P=0.017^a^ |  |  |  |
| 24 months (Change from baseline)** | -0.80 [-1.95 to 0.36] | P=0.177 | -0.67 [-2.00 to 0.67] | P=0.327^a^ |  |  |  |
| **WEIGHT** | | | | **BMI** | | | |
| **Emerenziani et al[26]** | **(IG) n=15** | **(CG) n=15** | **Between group difference** |  | **(IG) n=15** | **(CG) n=15** | **Between group difference** |
|  | **Mean (SD) Kg** | **Mean (SD) Kg** |  |  | **Mean (SD) Kg/m^2^** | **Mean (SD) Kg/m^2^** |  |
| Baseline | 87.6 (19.5) | 87.0 (22.6) |  | Baseline | 33.6 (7.7) | 32.6 (7.1) |  |
| 3 months | 85.0 (17.8)  P<0.05^b^ | 87.0 (22.1) | Not reported | 3 months | 32.6 (7.1)  P<0.05^b^ | 32.9 (5.3) | Not reported |
| **WEIGHT** | | | | |  |  |  |
| **Mayer-Davis et al[27]** | **IG1 n=49** | **IG2 n=47** | **CG n=56** | **Between group difference** |  |  |  |
|  | **Mean (SD) Kg** | **Mean (SD) Kg** | **Mean (SD) Kg** |  |  |  |  |
| Baseline | 99.5 (17.1) | 100.0 (19.8) | 93.0 (23.0) |  |  |  |  |
| 3 Months (change from baseline) | Not reported | Not reported | Not reported |  |  |  |  |
| 6 Months (change from baseline) | Value not reported  P<0.0001 | Value not reported  P=NS | Value not reported  P=NS | P<0.01 (IG1 vs CG)  P=NS (IG2 vs CG) |  |  |  |
| 12 Months (change from baseline) | -2.2kg  P<0.003 | Value not reported | -0.3kg | *-1.9* P=0.55 (IG1 vs CG)  P=NS (IG2 vs CG) |  |  |  |
|  |  |  | **BMI** | | | | |
|  |  |  |  | **IG 1 n=49** | **IG 2 n=47** | **CG n=56** | **Between group difference** |
|  |  |  |  | **Mean (SD) Kg/m2** | **Mean (SD) Kg/m2** | **Mean (SD) Kg/m2** |  |
|  |  |  | Baseline | 37.6 (6.5) | 37.5 (6.7) | 35.2 (7.5) |  |
|  |  |  | 6 Months (change from baseline) | -0.974  P=<0.001^b^ | -0.296  P=NS^b^ | -0.161  P=NS^b^ | IG1 vs CG P<0.01 |
|  |  |  | 12 Months | Not reported | Not reported | Not reported | Not reported |
| **WEIGHT** | | | | **BMI** | | | |
| **Modified fasting** | | | | | | | |
| **Weight or BMI reduction <5% at end of intervention** | | | | | | | |
| **Li et al[28]** | **(IG) n=23** | **(CG) n=23** | **Between group difference** |  | **(IG) n=23** | **(CG) n=23** | **Between group difference** |
|  | **Mean (SD) Kg** | **Mean (SD) Kg** | **[95% CI] Kg** |  | **Mean (SD) Kg/m^2^** | **Mean (SD) Kg/m^2^** | **Kg/m^2^ [95% CI]** |
| Baseline | 89.3 (12.6) | 95.3 (17.9) |  | Baseline | 30.7 (2.9) | 34.0 (6.7) |  |
| 4 months (change from baseline) | -3.5 (4.5)  P=0.01^b^ | -2.0 (4.8)  P=0.83^b^ | -3.0 [-6.0 to -0.4]  P=0.03^a^ | 4 months (change from baseline) | -1.2 (1.7)  P=0.16^b^ | -0.6 (2.6)  P=0.08^b^ | -1.0 [-2.0 to -0.1]  P=0.03^a^ |
| **Weight or BMI reduction >5% at end of intervention** | | | | | | | |
| **Oshakbayev et al[29]** | (IG) = 208 | (CG) = 64 |  |  | (IG) = 208 | (CG) = 64 |  |
|  | Mean (SE) Kg | Mean (SE) Kg |  |  | Mean (SE) Kg/m2 | Mean (SE) Kg/m2 |  |
| Baseline | 84.52 (0.75) | 84.49 (0.81) |  | Baseline | 29.60 (0.39) | 29.30 (0.47) |  |
| 6 months | 72.44 (1.02)  P<0.0001 | 81.35 (1.23)  P<0.05 |  | 6 months | 25.40 (0.37)  P<0.0001 | 28.04 (0.55) |  |
|  | Mean (SD) % | Mean (SD) % |  |  | Mean (SD) % | Mean (SD) % |  |
| 6 months (Change versus baseline) | -14.29 | -3.72 |  | 6 months (Change versus baseline) | -14.19 | -4.30 |  |
| **Low-calorie Meal replacements** | | | | | | | |
| **Low fat** | | | | | | | |
| **Weight or BMI reduction >5% at end of intervention** | | | | | | | |
| **Ash et al[30]** | (IG) = 14 | (IG2) = 20 | (IG3) = 17 |  |  |  |  |
|  | Mean (SD) Kg | Mean (SD) Kg | Mean (SD) Kg |  |  |  |  |
| Baseline | 96.7 (11.4) | 97.2 (13.5) | 101.4 (11.9) |  |  |  |  |
|  | All men |  |  |  |  |  |  |
| Baseline | 98.5 (12.3 |  |  |  |  |  |  |
| 12 weeks | 92.1 (11.4)  P<0.001  -6.4 (4.6)  -6.5% (6.0) |  |  |  |  |  |  |
| 18 month** | 96.7 (12.1)  P0.195 |  |  |  |  |  |  |
| **Cheskin et al[31]** | **(IG) n=54** | **(CG) n=58** | **Between group difference** |  | **(IG) n=54** | **(CG) n=58** | **Between group difference** |
|  | **Mean (SD) Kg** | **Mean (SD) Kg** |  |  | **Mean (SD) Kg/m^2^** | **Mean (SD) Kg/m^2^** |  |
| Baseline | 103.45 (14.18) | 101.3 (13.9) |  | Baseline | 35.7 (3.2) | 35.6 (3.0) |  |
| 34 weeks | 96.1 (13.0)  P<0.001^b^ | 97.6 (14.0)  P<0.001^b^ | *-3.65*  P = 0.039 | 34 weeks | 33.2 (3.4)  P<0.001^b^ | 34.3 (3.2)  P<0.001^b^ | *-1.2*  P =0.015 |
| Baseline (for those completing to 86 weeks) | 101.4 (16.0) | 100.2 (15.2) |  | Baseline (for those completing to 86 weeks) | 35.7 (3.9) | 35.0 (3.7) |  |
| 86 weeks | 95.8 (14.2)  P=0.006^b^ | 95.5 (14.7)  P=0.09^b^ | P=NS | 86 weeks | 33.8 (3.5)  P=0.008^b^ | 33.3 (3.3)  P=0.098^b^ | Not reported |
| **Delahanty et al[32]** | **(IG) n=28** | **(CG) n=29** |  |  |  |  |  |
|  | **Mean (SD) Kg** | **Mean (SD) Kg** |  |  |  |  |  |
| Baseline | 98.8 (15.5) | 96.3 (18.1) |  |  |  |  |  |
| 6 months (change from baseline) | -6.6 (7.0)  -6.58% (6.18) | -2.1 (3.5)  -2.22% (3.73) | P=0.004^a^  P=0.003^a^ |  |  |  |  |
| 12 months (change from baseline)** | -5.6% | -1.7% | P=0.008^a^ |  |  |  |  |
| **Foster et al[33]** | **(IG) n=50** | **(CG) n=50** |  |  | **(IG) n=50** | **(CG) n=50** |  |
|  | **Mean (SD) Kg**  **[95% CI]** | **Mean (SD) Kg**  **[95% CI]** |  |  | **Mean (SD) Kg/m^2^**  **[95% CI]** | **Mean (SD) kg/m^2^**  **[95% CI]** |  |
| Baseline | 101.8 (16.7) | 104.0 (20.1) |  | Baseline | 35.3 (4.6) | 36.2 (5.8) |  |
| 6 months | 93.9 (14.7) | 101.8 (19.4) | Not reported | 6 months | 32.6 (4.2) | 35.5 (5.8) | Not reported |
| Adjusted change (change from baseline) | -7.3 [-8.8 to -5.8] | -2.2 [-3.7 to -0.7] | P<0.0001^a^ | Adjusted Change (Change from baseline) | -2.5 [-3.0 to -2.0] | -0.7 [-1.2 to -0.2] | P<0.0001^a^ |
| *Foster - Adjusted change is reported as means (95% confidence intervals) and was obtained from linear mixed-effects models with time, treatment, and a time by treatment interaction included as explanatory variables* | | | | | | | |
| **Lean et al[34]** | **(IG) n=129** | **(CG) n=143** |  |  |  |  |  |
|  | **Mean (SD) Kg** | **Mean (SD) Kg** | **[95% CI]** |  |  |  |  |
| Baseline | 100.4 (16.5) | 98.7 (16.1) |  |  |  |  |  |
| 12 months  (Change from baseline) | 90.4 (16.4)  -10.0 (8.0) | 97.7 (16.4)  -1.0 (3.7) | -8.8 [-10.3 to -7.3]  P<0.0001^a^ |  |  |  |  |
| 24 months | 93.2 (17.2) | 96.4 (16.3) | -5.43 [-6.87 to -3.99]  P<0.0001 |  |  |  |  |
| **Look Ahead Study[35]** | **(IG) n=2570** | **(CG) n=2575** | **Between group difference** |  |  |  |  |
|  | **Mean (SD) Kg and/or Mean (SD) % change** | **Mean (SD) Kg**  **and/or Mean (SD) % change** | **% weight change [95% CI]** |  |  |  |  |
| Baseline | 101 (20) | 101 (19) |  |  |  |  |  |
| 12 months (change from baseline) | -8.6 (8.2)  -8.6% (6.9) | -0.7 (5.0)  -0.7% (4.8) | *-7.9*  -7.9% [-8.2 to -7.6]  P<0.001^a^ |  |  |  |  |
| 48 months (change from baseline) | -4.4% (0.2) | -0.7% (0.2) | P<0.0001^a^ |  |  |  |  |
| 96 months (change from baseline) | -4.7% (0.2) | -2.1% (0.2) | P<0.001^a^ |  |  |  |  |
| **Weight or BMI reduction not calculable** | | | | | | | |
| **Metz et al[36]** |  |  |  |  | (IG) = 56 | (CG) = 63 |  |
|  | (IG) = 56 | (CG) = 63 |  |  | Mean (SD) Kg/2 | Mean (SD) Kg/2 |  |
|  | Mean (SD) Kg | Mean (SD) Kg |  | Baseline | 33.0 (4.4) | 34.5 (4.5) |  |
| 3 months (change from baseline) | -4.7 (4.0) | -1.3 (2.5) |  |  |  |  |  |
| 6 months (change from baseline) | -5.5 (6.4) | -1.5 (3.2) |  |  |  |  |  |
| 12 months (change from baseline) | -3.0 (5.4) | -1.0 (3.8) |  |  |  |  |  |
| Between groups |  |  | P<0.001 |  |  |  |  |
| **Low carbohydrate** | | | | | | | |
| **Weight or BMI reduction >5% at end of intervention** | | | | | | | |
| **Goday et al[37]** | **(IG) n=45** | **(CG) n=44** | **Between group difference** |  | **(IG) n=45** | **(CG) n=446** | **Between group difference** |
|  | **Mean (SD) Kg** | **Mean (SD) Kg** |  |  | **Mean (SD) Kg/m^2^** | **Mean (SD) Kg/m^2^** |  |
| Baseline | 91.5 (11.4) | 90.0 (11.3) |  | Baseline | 33.3 (1.5) | 32.9 (1.60) |  |
| 4 months | 76.8 (9.1)  P<0.0001^b^ | 84.95 (13.96)  P=0.60^b^ | Not reported | 4 months | 27.9 (1.8)  P<0.0001^b^ | 31.0 (2.2)  P=<0.0001^b^ | Not reported |
| **Kempf 2 et al[38]** | **(IG) n=102** | **(CG) n=100** |  |  | **(IG) n=102** | **(CG) n=100** |  |
|  | **Mean (SD) Kg** | **Mean (SD) Kg** |  |  | **Mean (SD) Kg/m^2^** | **Mean (SD) Kg/m^2^** |  |
| Baseline | 104.3 (19.4) | 110.8 (21.1) |  | Baseline | 35.3 (5.9) | 37.0 (6.7) |  |
| 12 weeks | 98.1 (19.1)  -6.1 (4.6)  P<0.0001 | 109.8 (20.7)  -1.0 (3.4)  P=not reported | P<0.0001 | 12 weeks | 33.3 (6.0)  -2.1 (1.5)  P<0.0001 | 36.7 (6.6)  -0.3 (1.1)  P=not reported | P<0.0001 |
| 26 weeks** | 97.6 (19.2)  -6.7 (6.1)  P<0.0001 | 109.7 (20.1)  -1.1 (4.2)  P=not reported | P<0.0001 | 26 weeks** | 33.1 (6.1)  -2.2 (2.0)  P<0.0001 | 36.6 (6.5)  -0.4 (1.4)  P=not reported | P<0.0001 |
| 52 weeks** | 97.8 (19.2)  -6.5 (6.8)  P<0.0001 | 109.4 (20.3)  -1.4 (5.0)  P<0.05 | P<0.0001 | 52 weeks** | 33.2 (6.1)  -2.2 (2.2)  P<0.0001 | 36.5 (6.5)  -0.5 (1.6)  P<0.05 | P<0.0001 |
| **WEIGHT** | | | | |  |  |  |
| **Rock et al[39]** | **(IG) n=74** | **(IG2) n=77** | **(CG) n=76** | **Between group difference Kg** |  |  |  |
|  | **Mean (SD) Kg [95% CI] and/or Mean (SD) % change** | **Mean (SD) Kg [95% CI]**  **and/or Mean (SD) % change** | **Mean (SD) Kg**  **and/or Mean (SD) % change** |  |  |  |  |
| Baseline | 105.4 (17.8) | 106.4 (18.3) | 104.6 (16.9) |  |  |  |  |
| 6 months  (Change from baseline) | 96.5 (17.5)  -8.6% (5.9)  [7.2 to 10.0] | 95.0 (17.9)  -10.4% (6.9)  [8.9 to 12.0] | 102 (17.3)  -2.3% (4.2) | P<0.001^a^  P<0.001^a^ |  |  |  |
| 12 months  (Change from baseline) | 97.7 (18.0)  -7.4% (7.6) | 96.7 (19.7)  -9.0% (8.4) | 101.9 (17.4)  -2.5% (5.5) | P<0.001^a^  P<0.005^a^ |  |  |  |
|  |  |  | **BMI** | | | | |
|  |  |  |  | **(IG) n=74** | **(IG2) n=77** | **(CG) n=76** |  |
|  |  |  |  | **Mean (SD) Kg/m^2^** | **Mean (SD) Kg/m^2^** | **Mean (SD) Kg/m^2^** | **Between group difference** |
|  |  |  | Baseline | 36.2 (4.3) | 36.2 (4.7) | 36.3 (4.4) |  |
|  |  |  | 6 months | 33.2 (4.4) | 32.4 (4.8) | 35.5 (4.7) | P<0.001^a^ |
|  |  |  | 12 months | 33.5 (4.7) | 33.0 (5.5) | 35.4 (4.6) | P=0.001^a^ |
| **WEIGHT** | | | | **BMI** | | | |
| **Education and Physical activity** | | | | | | | |
| **Weight or BMI reduction <5% at end of intervention** | | | | | | | |
| **Fritz et al[40]** | **(IG) n=20** | **(CG) n=30** | **Between group difference** |  | **(IG) n=20** | **(CG) n=30** | **Between group difference** |
|  | **Mean (SD) Kg** | **Mean (SD) Kg** |  |  | **Mean (SD) Kg/m^2^** | **Mean (SD) Kg/m^2^** |  |
| Baseline | 91.9 (13.1) | 93.1 (15.7) |  | Baseline | 31.7 (5.2) | 31.1 (3.9) |  |
| 4 months (change from baseline) | -1.0 (2.1)  P <0.05^b^ | - 0.6 (2.2)  ^P value not reported^ | P=0.472^a^ | 4 months (change from baseline) | -0.4 (0.8)  P <0.05^b^ | -0.1 (1.0)  ^P value not reported^ | P=0.281^a^ |
| **Kempf et al[41]** | **(IG) n=120** | **(CG) n=100** |  |  | **(IG) n=120** | **(CG) n=100** | **Between group difference** |
|  | **Mean (SD) Kg** | **Mean (SD) Kg** |  |  | **Mean (SD) Kg/m^2^** | **Mean (SD) Kg/m^2^** |  |
| Baseline | 98 (19) | 96 (19) |  | Baseline | 34.1 (6.5) | 33.2 (6.3) |  |
| 3 months  (change from baseline) | 96 (19)  −1.2 (4.7)  P=0.0001^b^ | 96 (24)  −0.7 (2.6)  P=0.014^b^ | Not reported | 3 months  (change from baseline) | 33.5 (6.5)  −0.4 (1.6)  P=0.0001^b^ | 33.2 (6.5)  −0.3 (0.9)  P=0.007^b^ | Not reported |
| **Krousel-Wood et al[42]** |  |  |  |  | **(IG) n=45** | **(CG) n=49** | **Between group difference** |
|  |  |  |  |  | **Mean (SD) Kg/m^2^** | **Mean (SD) Kg/m^2^** |  |
|  |  |  |  | Baseline | 38.2 (8.2) | 37.0 (7.1) |  |
|  |  |  |  | 3 months | 37.8 (8.2) | 37.1 (7.2) | P = 0.06^a^ |
| **Van Dyck et al[43]** | **(IG) n=60** | **(CG) n=32** |  |  | **(IG) n=60** | **(CG) n=32** |  |
|  | **Mean (SD) Kg** | **Mean (SD) Kg** | **Between Group Difference** |  | **Mean (SD) Kg/m^2^** | **Mean (SD) Kg/m^2^** | **Between Group Difference** |
| Baseline | 89 (12.63) | 84.50 (12.68) |  | Baseline | 30.24 (2.62) | 29.74 (2.95) |  |
| 6 months | 89.20 (13.01) | 85.15 (13.03) | P=NS | 6 months | 30.23 (2.73) | 29.82 (3.23) | P=NS |
| 12 months** | 89.65 (13.47) | 84.82 (12.43) | P=NS | 12 months** | 30.39 (2.98) | 29.71 (3.39) | P=NS |
| **Motivational interviewing** | | | | | | | |
| **Weight or BMI reduction <5% at end of intervention** | | | | | | | |
| **West el al[44]** | **(IG) n=109** | **(CG) n=108** |  |  | **(IG) n=109** | **(CG) n=108** |  |
|  | **Mean (SE) Kg** | **Mean (SE) Kg** | **Between group difference** |  | **Mean (SE) Kg/m^2^** | **Mean (SE) Kg/m^2^** | **Between group difference** |
| Baseline | 97 (17) | 97 (15) |  | Baseline | 36.5 (5.5) | 36.5 (5.4) |  |
| 6 months (change from baseline) | -4.7 (0.45)  P=0.01^b^ | -3.1 (0.47)  P=0.03^b^ | P≥0.01^a^ |  |  |  |  |
| 12 months (change from baseline) | -4.8 (0.59) | -2.7 (0.62) | P≥0.02^a^ |  |  |  |  |
| 18 months (change from baseline) | -3.5 (0.62)  P=0.04^b^ | -1.7 (0.63)  P=0.04^b^ | P≥0.04^a^ |  |  |  |  |
| **Mindfulness** | | | | | | | |
| **Weight or BMI reduction <5% at end of intervention** | | | | | | | |
| **Miller et al[45]** | **(IG) n=27** | **(CG) n=25** |  |  | **(IG) n=27** | **(CG) n=25** |  |
|  | **Mean (SD) Kg** | **Mean (SD) Kg** |  |  | **Mean (SD) Kg/m^2^** | **Mean (SD) Kg/m^2^** |  |
| Baseline | 106.04 (3.66) | 103.38 (3.80) |  | Baseline | 36.19 (1.18) | 36.08 (1.22) |  |
| 3 months (change from baseline) | -1.78 (0.54)  P<0.0012^b^ | -3.25 (0.57)  P<0.0001^b^ | Not reported | 3 months (change from baseline) | -0.62 (0.19)  P=0.0014^b^ | -1.13 (0.20)  P<0.0001^b^ |  |
| 6 months (change from baseline) | -1.53 (0.54)  P=0.005^b^ | -2.92 (0.54)  P<0.001^b^ | P=0.0728^a^ | 6 months (change from baseline) | -0.53 (0.19)  P=0.0058^b^ | -1.03 (0.19)  P<0.0001^b^ | P=0.0678^a^ |
| **Moncrieft et al[46]** | **(IG) = 57** | **(CG) = 54** |  |  | **(IG) = 54** | **(CG) = 57** |  |
|  | Mean (SD) Kg | Mean (SD) Kg |  |  | Mean (SD) Kg/m2 | Mean (SD) Kg/m2 |  |
| Baseline | 85.04 (12.22) | 85.57 (16.20) |  |  | 32.3 (3.7) | 32.9 (5.5) |  |
| 6 months | 81.78 (12.56) | 85.21 (16.05) |  |  |  |  |  |
| 12 months | 82.03 (12.58) | 84.19 (15.48) |  |  |  |  |  |
| **Coaching** | | | | | | | |
| **Weight or BMI reduction <5% at end of intervention** | | | | | | | |
| **Weight** | | | | | |  |  |
| **Bollyky et al[48]** | **(IG1) n=64** | **(IG2) n=67** | **(CG) n=90** |  |  |  |  |
|  | **Mean (SD) Llbs** | **Mean (SD) Llbs** | **Mean (SD) Llbs** | Between group **difference** |  |  |  |
| Baseline | 244 (55) | 246 (49) | 224 (52) | P=0.02 |  |  |  |
| 12 weeks | 238 (53)  -6.4 (9.7) | 242 (49)  -4.1 (9.4) | 223 (50)  -1.1 (13.7) | P=0.06  P=0.02 |  |  |  |
| **Weight** | | | | **BMI** | | | |
| **Odnoletkova et al[49]** | **(IG) n=287** | **(CG) n=287** |  |  | **(IG) n=287** | **(CG) n=287** |  |
|  | **Mean (SD) Kg** | **Mean (SD) Kg** | **Between group difference Mean [95% CI]** |  | **Mean (SD) Kg/m^2^** | **Mean (SD) Kg/m^2^** | **Between group difference Mean [95% CI]** |
| Baseline | 86.1(16.9) | 88.3(16.6) |  | Baseline | 30.2(4.9) | 30.6(5.2) |  |
| 6 months | 84.8(16.4) | 87.0(15.9) | -1.1[-1.9 to -0.4]  P=0.004^a^ | 6 months | 29.6(4.9) | 30.4(5.1) | -0.4 [-0.6 to -0.1]  P=0.003^a^ |
| 18 months** | 85.9(16.6) | 87.3(15.4) | -0.2[-1.2 to 0.8]  P=0.690^a^ | 18 months** | 29.9(5.0) | 30.4(5.1) | -0.1[-0.4 to 0.2]  P=0.602^a^ |
| **Safford et al[47]** |  |  |  |  | **(IG) n=198** | **(CG) n=226** |  |
|  |  |  |  |  | **Mean (SD) Kg/m^2^** | **Mean (SD) Kg/m^2^** |  |
|  |  |  |  | Baseline | 36.5 (7.7) | 36.0 (9.1) |  |
|  |  |  |  | 15 months (change from baseline) | -0.23 (2.4) | -0.49 (2.8) | P=0.44 |
| **Key:**  **a: p value for between group difference; b: p value for within group change from baseline; c: p value for difference between groups over all time points; d: p value not reported**  **95% CI: 95% Confidence interval, SD: Standard deviation, SE: Standard error**  **Shaded cells represent values recorded during maintenance period, **: Extended follow up period post intervention (not maintenance intervention)**  **P=NS: actual value not provided** | | | | | | | |

1. Adolfsson ET, Walker-Engström M-L, Smide B, Wikblad K. Patient education in type 2 diabetes—a randomized controlled 1-year follow-up study. Diabetes research and clinical practice. 2007;76(3):341-50.

2. Cheyette C. Weight No More: a randomised controlled trial for people with type 2 diabetes on insulin therapy. Practical Diabetes. 2007;24(9):450-6.

3. Wolf A, Conaway M, Crowther J, Hazen K, Nadler J, Oneida B et al. Improving Control with Activity and Nutrition (ICAN) Study. Translating lifestyle intervention to practice in obese patients with type 2 diabetes: Improving Control with Activity and Nutrition (ICAN) study. Diabetes Care. 2004;27(7):1570-6.

4. Samuel-Hodge CD, Holder-Cooper JC, Gizlice Z, Davis G, Steele SP, Keyserling TC et al. Family PArtners in Lifestyle Support (PALS): Family-based weight loss for African American adults with type 2 diabetes. Obesity. 2017;25(1):45-55. doi:<https://dx.doi.org/10.1002/oby.21700>.

5. Campbell EM, Redman S, Moffitt P, Sanson-Fisher RW. The relative effectiveness of educational and behavioral instruction programs for patients with NIDDM: a randomized trial. The Diabetes Educator. 1996;22(4):379-86.

6. D'Eramo-Melkus GA, Wylie-Rosett J, Hagan JA. Metabolic impact of education in NIDDM. Diabetes Care. 1992;15(7):864-9.

7. Mitra A, Dewanjee D, Dey B. Mechanistic studies of lifestyle interventions in type 2 diabetes. World journal of diabetes. 2012;3(12):201.

8. Orsama A-L, Lähteenmäki J, Harno K, Kulju M, Wintergerst E, Schachner H et al. Active assistance technology reduces glycosylated hemoglobin and weight in individuals with type 2 diabetes: results of a theory-based randomized trial. Diabetes technology & therapeutics. 2013;15(8):662-9.

9. Prezio EA, Cheng D, Balasubramanian BA, Shuval K, Kendzor DE, Culica D. Community Diabetes Education (CoDE) for uninsured Mexican Americans: a randomized controlled trial of a culturally tailored diabetes education and management program led by a community health worker. Diabetes research and clinical practice. 2013;100(1):19-28.

10. Allen NA, Fain JA, Braun B, Chipkin SR. Continuous glucose monitoring counseling improves physical activity behaviors of individuals with type 2 diabetes: a randomized clinical trial. Diabetes research and clinical practice. 2008;80(3):371-9.

11. Christian JG, Bessesen DH, Byers TE, Christian KK, Goldstein MG, Bock BC. Clinic-based support to help overweight patients with type 2 diabetes increase physical activity and lose weight. Archives of Internal Medicine. 2008;168(2):141-6.

12. O'Neil PM, Miller‐Kovach K, Tuerk PW, Becker LE, Wadden TA, Fujioka K et al. Randomized controlled trial of a nationally available weight control program tailored for adults with type 2 diabetes. Obesity. 2016;24(11):2269-77.

13. Brehm BJ, Lattin BL, Summer SS, Boback JA, Gilchrist GM, Jandacek RJ et al. One-year comparison of a high-monounsaturated fat diet with a high-carbohydrate diet in type 2 diabetes. Diabetes care. 2009;32(2):215‐20. doi:10.2337/dc08-0687.

14. Daly ME, Paisey R, Millward B, Eccles C, Williams K, Hammersley S et al. Short‐term effects of severe dietary carbohydrate‐restriction advice in Type 2 diabetes—a randomized controlled trial. Diabetic Medicine. 2006;23(1):15-20.

15. Davis NJ, Tomuta N, Schechter C, Isasi CR, Segal-Isaacson C, Stein D et al. Comparative study of the effects of a 1-year dietary intervention of a low-carbohydrate diet versus a low-fat diet on weight and glycemic control in type 2 diabetes. Diabetes care. 2009;32(7):1147-52.

16. Guldbrand H, Dizdar B, Bunjaku B, Lindström T, Bachrach-Lindström M, Fredrikson M et al. In type 2 diabetes, randomisation to advice to follow a low-carbohydrate diet transiently improves glycaemic control compared with advice to follow a low-fat diet producing a similar weight loss. Diabetologia. 2012;55(8):2118-27.

17. Iqbal N, Vetter ML, Moore RH, Chittams JL, Dalton-Bakes CV, Dowd M et al. Effects of a low-intensity intervention that prescribed a low-carbohydrate vs. a low-fat diet in obese, diabetic participants. Obesity (silver spring, md). 2010;18(9):1733‐8. doi:10.1038/oby.2009.460.

18. Vuksan V, Jenkins A, Brissette C, Choleva L, Jovanovski E, Gibbs A et al. Salba-chia (Salvia hispanica L.) in the treatment of overweight and obese patients with type 2 diabetes: A double-blind randomized controlled trial. Nutrition, Metabolism and Cardiovascular Diseases. 2017;27(2):138-46.

19. Fabricatore AN, Wadden TA, Ebbeling CB, Thomas JG, Stallings VA, Schwartz S et al. Targeting dietary fat or glycemic load in the treatment of obesity and type 2 diabetes: a randomized controlled trial. Diabetes research and clinical practice. 2011;92(1):37‐45. doi:10.1016/j.diabres.2010.12.016.

20. Luley C, Blaik A, Reschke K, Klose S, Westphal S. Weight loss in obese patients with type 2 diabetes: Effects of telemonitoring plus a diet combination–The Active Body Control (ABC) Program. Diabetes research and clinical practice. 2011;91(3):286-92.

21. Tay J, Thompson CH, Luscombe-Marsh ND, Wycherley TP, Noakes M, Buckley JD et al. Effects of an energy-restricted low-carbohydrate, high unsaturated fat/low saturated fat diet versus a high-carbohydrate, low-fat diet in type 2 diabetes: a 2-year randomized clinical trial. Diabetes, obesity & metabolism. 2018;20(4):858‐71. doi:10.1111/dom.13164.

22. Krebs JD, Elley CR, Parry-Strong A, Lunt H, Drury PL, Bell DA et al. The Diabetes Excess Weight Loss (DEWL) Trial: a randomised controlled trial of high-protein versus high-carbohydrate diets over 2 years in type 2 diabetes. Diabetologia. 2012;55(4):905‐14. doi:10.1007/s00125-012-2461-0.

23. Pedersen et al. A portion-control plate was effective for weight loss in obese patients with type 2 diabetes mellitus. ACP Journal Club. 2007;147(3):68-.

24. Uusitupa M, Laitinen J, Siitonen O, Vanninen E, Pyörälä K. The maintenance of improved metabolic control after intensified diet therapy in recent type 2 diabetes. Diabetes research and clinical practice. 1993;19(3):227-38.

25. Eakin EG, Reeves MM, Marshall AL, Dunstan DW, Graves N, Healy GN et al. Living Well with Diabetes: a randomized controlled trial of a telephone-delivered intervention for maintenance of weight loss, physical activity and glycaemic control in adults with type 2 diabetes. BMC public health. 2010;10(1):452.

26. Emerenziani GP, Gallotta MC, Meucci M, Di Luigi L, Migliaccio S, Donini LM et al. Effects of aerobic exercise based upon heart rate at aerobic threshold in obese elderly subjects with type 2 diabetes. International journal of endocrinology. 2015;2015.

27. Mayer-Davis EJ, D'antonio AM, Smith SM, Kirkner G, Levin Martin S, Parra-Medina D et al. Pounds off with empowerment (POWER): a clinical trial of weight management strategies for black and white adults with diabetes who live in medically underserved rural communities. American journal of public health. 2004;94(10):1736-42.

28. Li C, Sadraie B, Steckhan N, Kessler C, Stange R, Jeitler M et al. Effects of a one-week fasting therapy in patients with type-2 diabetes mellitus and metabolic syndrome–A randomized controlled explorative study. Experimental and Clinical Endocrinology & Diabetes. 2017;125(09):618-24.

29. Oshakbayev K, Dukenbayeva B, Togizbayeva G, Durmanova A, Gazaliyeva M, Sabir A et al. Weight loss technology for people with treated type 2 diabetes: a randomized controlled trial. Nutrition & metabolism. 2017;14(1):1‐9. doi:10.1186/s12986-017-0163-9.

30. Ash S, Reeves MM, Yeo S, Morrison G, Carey D, Capra S. Effect of intensive dietetic interventions on weight and glycaemic control in overweight men with Type II diabetes: a randomised trial. International journal of obesity and related metabolic disorders. 2003;27(7):797‐802. doi:10.1038/sj.ijo.0802295.

31. Cheskin LJ, Mitchell AM, Jhaveri AD, Mitola AH, Davis LM, Lewis RA et al. Efficacy of meal replacements versus a standard food-based diet for weight loss in type 2 diabetes a controlled clinical trial. The Diabetes Educator. 2008;34(1):118-27.

32. Delahanty LM, Dalton KM, Porneala B, Chang Y, Goldman VM, Levy D et al. Improving diabetes outcomes through lifestyle change–A randomized controlled trial. Obesity. 2015;23(9):1792-9.

33. Foster G, Wadden T, Lagrotte C, Vander Veur S, Hesson L, Homko C et al. A randomized comparison of a commercially available portion-controlled weight-loss intervention with a diabetes self-management education program. Nutrition & diabetes. 2013;3(3):e63.

34. Lean ME, Leslie WS, Barnes AC, Brosnahan N, Thom G, McCombie L et al. Primary care-led weight management for remission of type 2 diabetes (DiRECT): an open-label, cluster-randomised trial. The Lancet. 2017.

35. Pi-Sunyer X. The Look AHEAD Trial: A Review and Discussion of Its Outcomes. Current Nutrition Reports. 2014;3(4):387-91. doi:<http://dx.doi.org/10.1007/s13668-014-0099-x>.

36. Metz JA, Stern JS, Kris-Etherton P, Reusser ME, Morris CD, Hatton DC et al. A randomized trial of improved weight loss with a prepared meal plan in overweight and obese patients: impact on cardiovascular risk reduction. Archives of internal medicine. 2000;160(14):2150-8.

37. Goday A, Bellido D, Sajoux I, Crujeiras A, Burguera B, García-Luna PP et al. Short-term safety, tolerability and efficacy of a very low-calorie-ketogenic diet interventional weight loss program versus hypocaloric diet in patients with type 2 diabetes mellitus. Nutrition & diabetes. 2016;6(9):e230.

38. Kempf K, Altpeter B, Berger J, Reus O, Fuchs M, Schneider M et al. Efficacy of the Telemedical Lifestyle intervention Program TeLiPro in Advanced Stages of Type 2 Diabetes: A Randomized Controlled Trial. Diabetes Care. 2017;40(7):863-71. doi:<https://dx.doi.org/10.2337/dc17-0303>.

39. Rock CL, Flatt SW, Pakiz B, Taylor KS, Leone AF, Brelje K et al. Weight loss, glycemic control, and cardiovascular disease risk factors in response to differential diet composition in a weight loss program in type 2 diabetes: a randomized controlled trial. Diabetes care. 2014;37(6):1573-80.

40. Fritz T, Caidahl K, Krook A, Lundström P, Mashili F, Osler M et al. Effects of Nordic walking on cardiovascular risk factors in overweight individuals with type 2 diabetes, impaired or normal glucose tolerance. Diabetes/metabolism research and reviews. 2013;29(1):25-32.

41. Kempf K, Martin S. Autonomous exercise game use improves metabolic control and quality of life in type 2 diabetes patients-a randomized controlled trial. BMC endocrine disorders. 2013;13(1):57.

42. Krousel-Wood M, Berger L, Jiang X, Blonde L, Myers L, Webber L. Does home-based exercise improve body mass index in patients with type 2 diabetes?: Results of a feasibility trial. Diabetes research and clinical practice. 2008;79(2):230-6.

43. Van Dyck D, De Greef K, Deforche B, Ruige J, Bouckaert J, Tudor-Locke CE et al. The relationship between changes in steps/day and health outcomes after a pedometer-based physical activity intervention with telephone support in type 2 diabetes patients. Health education research. 2013;28(3):539-45.

44. West DS, DiLillo V, Bursac Z, Gore SA, Greene PG. Motivational interviewing improves weight loss in women with type 2 diabetes. Diabetes care. 2007;30(5):1081-7.

45. Miller CK, Kristeller JL, Headings A, Nagaraja H, Miser WF. Comparative effectiveness of a mindful eating intervention to a diabetes self-management intervention among adults with type 2 diabetes: a pilot study. Journal of the Academy of Nutrition and Dietetics. 2012;112(11):1835-42.

46. Moncrieft AE, Llabre MM, McCalla JR, Gutt M, Mendez AJ, Gellman MD et al. Effects of a Multicomponent Life-Style Intervention on Weight, Glycemic Control, Depressive Symptoms, and Renal Function in Low-Income, Minority Patients With Type 2 Diabetes: results of the Community Approach to Lifestyle Modification for Diabetes Randomized Controlled Trial. Psychosomatic medicine. 2016;78(7):851‐60. doi:10.1097/PSY.0000000000000348.

47. Safford MM, Andreae S, Cherrington AL, Martin MY, Halanych J, Lewis M et al. Peer coaches to improve diabetes outcomes in rural Alabama: a cluster randomized trial. The Annals of Family Medicine. 2015;13(Suppl 1):S18-S26.

48. Bollyky JB, Bravata D, Yang J, Williamson M, Schneider J. Remote Lifestyle Coaching Plus a Connected Glucose Meter with Certified Diabetes Educator Support Improves Glucose and Weight Loss for People with Type 2 Diabetes. Journal of diabetes research. 2018;2018:3961730. doi:10.1155/2018/3961730.

49. Odnoletkova I, Goderis G, Nobels F, Fieuws S, Aertgeerts B, Annemans L et al. Optimizing diabetes control in people with Type 2 diabetes through nurse‐led telecoaching. Diabetic Medicine. 2016;33(6):777-85.
